# Taxonomic revision of the genus *Copelatus* of Madagascar (Coleoptera, Dytiscidae, Copelatinae): the non- *erichsonii* group species

**DOI:** 10.3897/zookeys.869.33997

**Published:** 2019-08-05

**Authors:** Tolotra Ranarilalatiana, Lala Harivelo Raveloson Ravaomanarivo, Johannes Bergsten

**Affiliations:** 1 Department of Entomology, Faculty of Sciences, Box 906, Antananarivo University, 101 Antananarivo, Madagascar Antananarivo University Antananarivo Madagascar; 2 Department of Zoology, Swedish Museum of Natural History, Box 50007, SE-10405 Stockholm, Sweden Swedish Museum of Natural History Stockholm Sweden

**Keywords:** Conservation, distribution, diving beetles, freshwater, gene tree, GMYC, new combination, new species, new synonymy, phylogeny, species delimitation

## Abstract

The genus *Copelatus* Erichson, 1832 (Coleoptera, Dytiscidae, Copelatinae) of Madagascar is revised in two parts. This review is restricted to the *Copelatus* species that have fewer than ten elytral + one submarginal stria, including all species except those of the *erichsonii* species group. Both morphological and molecular (mitochondrial COI) data are used in an integrative taxonomic approach. Thirteen species are recognised, of which five are described as new: *Copelatus ankaratra***sp. nov.**, *Copelatus kely***sp. nov.**, *Copelatus pseudostriatus***sp. nov.**, *Copelatus safiotra***sp. nov.** and *Copelatus vokoka***sp. nov.***Copelatus unguicularis* Régimbart, 1903 and *Copelatus apicalis* Fairmaire, 1898 are both transferred to the genus *Madaglymbus* Shaverdo & Balke, 2008 (**comb. nov.**). *Copelatus mimetes*Guignot 1957 is a junior synonym of the widespread Afrotropical–Arabian *Copelatus pulchellus* (Klug, 1834) (**syn. nov.**). *Copelatus marginipennis* (Laporte, 1835) is reinstated (**stat. nov.**) as a valid species with *Copelatus aldabricus* Balfour-Browne, 1950 and Copelatus aldabricus var. simplex Guignot, 1952 as junior synonyms (**syn. nov.**). We designate lectotypes for *Colymbetes marginipennis* Laporte, 1835 and *Copelatus obtusus* Boheman, 1848. *Copelatus peridinus* Guignot, 1955 is recorded for Madagascar for the first time and *Copelatus nodieri* Régimbart, 1895 is rejected as a species present in Madagascar.

## Introduction

The subfamily Copelatinae of diving beetles is a diverse group of aquatic beetles represented by eight genera: *Agaporomorphus* Zimmermann, 1921, *Aglymbus* Sharp, 1880, *Capelatus* Turner & Bilton, 2015, *Copelatus* Erichson, 1832, *Exocelina* Broun, 1886, *Lacconectus* Motschulsky, 1855, *Liopterus* Dejean, 1833, and *Madaglymbus* Shaverdo & Balke, 2008. This subfamily is relatively homogeneous morphologically, and only the single tribe Copelatini is recognised (Miller 2001; Miller and Bergsten 2014, 2016). They are one of the most commonly encountered water beetles in many wet tropical and subtropical forests (Balke et al. 2004). After the revised circumscription of *Aglymbus* and the erection of the new genus *Madaglymbus* (Shaverdo et al. 2008; Miller and Bergsten 2016), this subfamily is represented by two genera on Madagascar: *Copelatus* and *Madaglymbus*. *Copelatus* is the most species-rich genus of Dytiscidae in the world, distributed throughout all zoogeographical regions and currently comprises 442 recognised species (Nilsson and Hájek 2018). Rocchi (1991) listed 22 species of *Copelatus* from Madagascar, 18 as endemic, after which only one new endemic species has been added by Pederzani & Hájek (2005) and one originally mislabelled species removed from the fauna by Balke et al. (2014).

To be able to deal with this diversity, *Copelatus* species have traditionally been organised in a number of species groups based on the number of elytral and submarginal striae following Sharp (1882; Zimmermann 1919, 1934; Balfour-Browne 1939; Guignot 1961; Guéorguiev 1968; Nilsson et al. 1997; Nilsson 2001). This is today a practical and not always a phylogenetically sound division (Balke et al. 2004). In fact Balke et al. (2004), reconstructing a phylogeny of Copelatinae with mitochondrial CO1 and 16S, found the number of elytral striae to be a highly variable and homplastic character. High variability in number of striae, both intraspecifically and between closely related species, was recently documented in Antillean species (Manuel et al. 2018).

The species that occur in Madagascar fall into five of these species groups: the *hydroporoides* (= formerly *haemorrhoidalis*, no elytral striae), *longicornis* (3, 4 or 5 elytral striae), *irinus* (6 elytral + 1 submarginal stria), *consors* (10 elytral striae) and *erichsonii* (10 elytral + 1 submarginal stria) groups. About half of the described species are members of the *erichsonii* species group, and the other half of the remaining species groups with fewer striae. In this paper, we will treat the *Copelatus* species of Madagascar with fewer than 10 elytral and one submarginal stria, i.e., all the non-*erichsonii* group species. Again, this is a practical rather than a phylogenetic division and in fact *Copelatus safiotra* sp. nov., described below, we believe is most closely related to the species of the *erichsonii* group due to the shape of male genitalia.

This study is motivated by recent collecting efforts of aquatic beetles in Madagascar 2009–2018 in the Water Beetles of Madagascar Project. The project is a collaboration between the Swedish Museum of Natural History and the University of Antananarivo. The current study is based on this rich new material containing five new species of the *irinus* species group, together with type material and some additional museum accessions. We use both morphological and molecular data with a species delimitation analysis, in an integrative taxonomic approach to the revision. We provide an identification key and for each species a diagnosis, description, known distribution and habitat and ecology notes. Each species is also illustrated with a dorsal habitus photograph, and ventral and lateral views of male penis and parameres.

## Materials and methods

### Fieldwork

**Figure 1.**
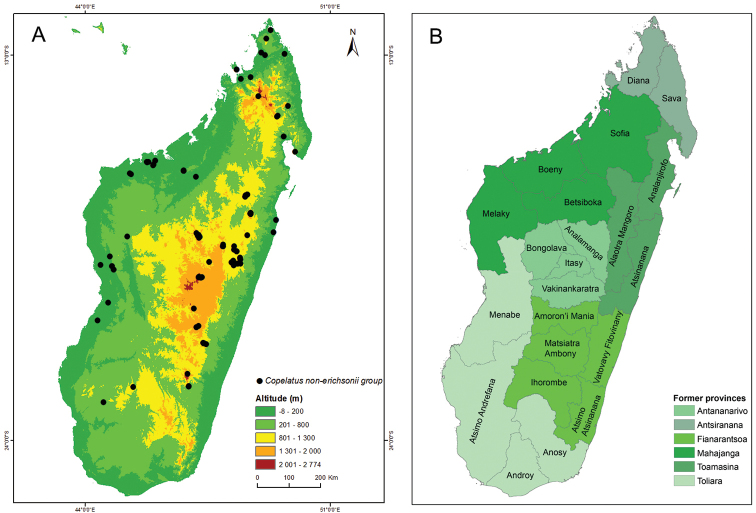
Maps of Madagascar with records and first level administrative divisions. **A** All localities of examined *Copelatus* specimens of non-*erichsonii* group species **B** The 22 current regions and six former provinces of Madagascar.

New collecting efforts of aquatic beetles were conducted mainly in National Parks, reserves and natural forests but also in degraded forests, open areas and along main roads from all parts of Madagascar, except scant from the very south (Fig. 1A). The fieldwork was supported by permits from the “Ministère de l’Environnement, de l’Ecologie et des Forêts”. We collected in various habitats ranging from large rivers to small streams, cascades and hygropetric rocks, forest pools, rock pools, ditches and canals, ponds, marshes, and lakes. *Copelatus* can be found in diverse aquatic habitats, both running (lotic) and standing (lentic) waters. Along forest streams they are best sought for in stagnant parts such as side-pools and rock pools with dead leaves or vegetation or in residual pools in a partly dried out streambed. Leaf-choked forest pools, vegetation-rich edges of ponds and lakes and marshes are also very good habitats for *Copelatus*. Some species are very good fliers and readily come to light, but the proportion of light catches (using a 22W black-ringlight) in the studied material is relatively small. A surprising number can be found even in minute seeps especially if visited with head lamp at night when they are more active and swim around. *Copelatus* was sampled mainly using white pans and GB water nets and sieves with various sizes depending on waterbody type. GB water net is often best for larger habitats while sieves were used for sampling smaller habitats like rockpools. Specimens were collected into plastic tubes with 95% ethanol for conservation.

Each locality was given a collecting event code and associated metadata included geographic name(s), forest type, waterbody type, habitat description, eventual disturbance, collecting date and collectors. Altitude, latitude and longitude were recorded with a handheld GPS (Garmin). Each locality was also documented with photographs using compact Canon and Olympus digital cameras.

### Madagascar administrative divisions

Madagascar is since the 2007 revision of the constitution divided into 22 regions as a first level administrative division, followed by districts as a second level, a recent change from the former six provinces as first level (Fig. 1B). Studied material is listed in sections preceeded by geographical divisions (former province. region: district1, district2.) to give records by both current and, the faunistically much longer used, former first level divisions.

### Depositories

Most of the studied material came from the new fieldwork and is shared between the Swedish Museum of Natural History (**NHRS**), Parc Botanique et Zoologique de Tsimbazaza/ Madagascar Biodiversity Center (**PBZT/MBC**) and Department of Entomology, Antananarivo University (**DEUA**). Some material originated from efforts organised by the Natural History Museum in London (**NHMUK**) 2004–2007.

Additional studied material came from earlier expeditions housed at museum collections in Museum National d’Histoire Naturelle, Paris (**MNHN**), Museum of Natural History, London (NHMUK), California Academy of Sciences (**CAS**), Naturhistorisches Museum, Vienna (**NMW**), and National Museum (Natural History), Prague (**NMPC**). Studied older types are housed in Paris (MNHN), London (NHMUK), Stockholm (NHRS) and Prague (NMPC), while holotypes of newly decribed species are housed in Stockholm (NHRS).

Depositories of studied specimens are referred to by above abbreviations (see Suppl. material 1). Long paratype series for new species with “NHRS, DEUA & PBZT/MBC” given as depository will also be shared with other central institution collections.

### Morphology

Specimens were examined under dissection microscopes from Leica (M165C and MZ12.5). Genitalia were extracted with a fine forceps or pin from the tip of the abdomen and glued onto cards on the same pin as the specimen. Dry-preserved specimens were first relaxed in warm water for 5–20 minutes before genitalia were carefully extracted. Photos of habitus were taken with a Canon EOS 5D Mark II DSLR camera equipped with a Canon MP-E 65mm 1–5X super macrolens and mounted on a motorised rail (Stackshot) from Cognisys. The system was operated using Canon EOS Utility and Zerene Stacker (Zerene Systems) softwares, the latter also used for stacking the Z-stack of captured images with the PMax or DMap algorithms. Photographs of dry-mounted genitalia were taken with a Canon EOS 7D DSLR camera mounted on a BALPRO 1 Universal bellow from Novoflex with a long working distance 10X Plano apochromatic microscope objective from Mitutoyo. The bellow was mounted on a motorised rail (Stackshot) from Cognisys and operated with the same software given above.

In describing the male penis we use the terminology suggested by Miller and Nilsson (2003).

Label data are given as written and separated by “//” if on separate labels and “|” if on different rows on the same label. Text within square brackets “[]” are our comments, explications or interpretations. Most examined specimens (individual mounted specimens, or single alcohol tubes with multiple specimens) have been given unique catalogue numbers and these are listed first, starting with “NHRS”, “BMNH” (=NHMUK) or “CAS”, followed by a number. A series of specimens with consecutive catalogue numbers are given as a range. The following additional abbreviations are used:

**Alc.** in alcohol tube,

**Ex.** exemplars (number of individuals),

**GP** (Genital Preparation) male genitalia have been examined,

**HT** Holotype,

**LT** Lectotype,

**PLT** Paralectotype,

**PT** Paratype,

**ST** Syntype,

**TL** Type locality,

### DNA extraction and PCR

DNA was extracted from one mesoleg or from soft abdominal tissue retrieved in association with dissection of male genitalia. The leg or soft tissue was incubated in lysis buffer at 56 °C overnight. Post-incubation protocol followed the cell and tissue DNA kit on a KingFisher Duo Prime. This system provides automated nucleic acid purification at a running time of approx. 25 minutes.

We used ready-to-go beads to prepare 25 μl PCR reactions consisting of 21 μl of water, 1 μl of each primer and 2 μl of DNA template. We used the primers Jerry (F, 5’-CAA CAT TTA TTT TGA TTT TTT GG-3’) and PatDyt (R, 5’-TCA TTG CAC TAA TCT GCC ATA TTA G-3’) to amplify an 825 bp fragment of mitochondrial cytochrome c-oxidase subunit 1 gene (COI or *cox1*) (Simon et al. 1994; Isambert et al. 2011). For two dry-mounted specimens, *Copelatus* sp. female (Bemaraha) (CASENT-8135000) and *Copelatus* sp. female (Andasibe) (NHRS-JLKB000065698), we used the primers Jerry (F, 5’-CAA CAT TTA TTT TGA TTT TTT GG-3’) – Hal 1450rw (R, 5’-GGA AAT CAT TGA ATA AAT CCA GCT-3’); and Hal 1450fw (F, 5’-AGC TGG ATT TAT TCA ATG ATT TCC-3’) – PatDyt (R, 5’-TCA TTG CAC TAA TCT GCC ATA TTA G-3’) to amplify the same segment but in two shorter sections (Wallin et al. 2017). The PCR programme started with 95 °C for 5 min, followed by 40 cycles of 95 °C for 30 s, 50 °C for 30 s, and 72 °C for 50 s. A final extension step of 72 °C for 8 min followed the 40 cycles, and reactions were then stored in the block at 12 °C. Agarose gel electrophoresis was used to verify amplifications, including a negative control.

Successful PCR products were purified using EXOSAP Clean-up mix of two enzymes (Exonuclease and Shrimp Alkaline Phosphatase) and run in a PCR machine with the programme 37 °C for 30 min followed by 80 °C for 15 min and finally 12 °C (∞). PCR products were sent to Macrogen for sequencing.

Gene regions were sequenced in both directions and sequence chromatograms were edited with SEQUENCHER version 4.10.1 (Gene Codes Corporation). The contigs were assembled from forward and reverse reads, and primer regions trimmed. New sequence data were then exported in fasta format and aligned together with GenBank sequences of *Copelatus* from Isambert et al. (2011) in CLUSTALX using default settings. All new sequences are available in Genbank under accession numbers MK878825-MK878877 (Table 1).

**Table 1.** Details of material used for DNA analysis and GenBank accession numbers for mitochondrial COI. New sequences submitted to GenBank have accession numbers starting with “MK”. For samples without a separate extract number, the extract is identified by the ID Cat. No.

Species	ID Cat. No.	Extract	Field ID	Place	Lat/Long	Alt	Date	Accession numbers
* C. marginipennis *	BMNH-797876	294:A4	P57BI31	Marojejy NP	14.4573S, 49.7908E	162	10/12/07	HQ382912
	BMNH-797894	294:B10	P61BI15	Andasibe NP	18.9375S, 48.4167E	940	06/01/07	HQ382926
	BMNH-797906	294:C10	P58BI14	Masoala NP, E. of Marofototra	15.7587S, 49.9932E	10	17/12/06	HQ382937
	BMNH-797907	294:C11	P58BI14	Masoala NP, E. of Marofototra	15.7587S, 49.9932E	10	17/12/06	HQ382938
	BMNH-797908	294:C12	P58BI14	Masoala NP, E. of Marofototra	15.7587S, 49.9932E	10	17/12/06	HQ382939
	BMNH-797909	294:D01	P58BI14	Masoala NP, E. of Marofototra	15.7587S, 49.9932E	10	17/12/06	HQ382940
	BMNH-797910	294:D02	P58BI14	Masoala NP, E. of Marofototra	15.7587S, 49.9932E	10	17/12/06	HQ382941
	NHRS-JLKB000010729	JB196	MAD09-07	Ankarafantsika NP, Ampijoroa	16.3034S, 46.8107E	74	29/11/09	MK878864
	NHRS-JLKB000010730	JB197	MAD09-46	Kirindy Res.	20.0743S, 4.6631E	52	12/12/09	MK878871
	NHRS-JLKB000010731	JB198	MAD09-13	Ankarafantsika NP, Ampijoroa	16.3027S, 46.8100E	75	30/11/09	MK878865
	NHRS-JLKB000010732	JB199	MAD09-07	Ankarafantsika NP, Ampijoroa	16.3034S, 46.8107E	74	29/11/09	MK878869
	NHRS-JLKB000010733	JB200	MAD09-59	Tsingy de Bemaraha NP, Bekopaka	19.0342S, 44.7750E	41	15/12/09	MK878872
	NHRS-JLKB000010734	JB201	MAD09-03	Ankarafantsika NP, Ampijoroa	16.3035S, 46.8107E	87	29/11/09	MK878870
	NHRS-JLKB000010735	JB202	MAD09-29	Mahavavy Kinkony Res., Mitsinjo	16.0665S, 45.7767E	24	05/12/09	MK878866
	NHRS-JLKB000010736	JB203	MAD09-65	Tsingy de Bemaraha NP, Antsalova	18.7564S, 44.7140E	119	17/12/09	MK878873
	NHRS-JLKB000010737	JB191	MAD09-14	Ankarafantsika NP, Ampijoroa	16.3142S, 6.8173E	77	30/11/09	MK878862
	NHRS-JLKB000010738	JB192	MAD09-24	Mahavavy Kinkony Res., Makary village	16.1465S, 45.9493E	9	04/12/09	MK878863
	NHRS-JLKB000010739	JB193	MAD09-25	Mahavavy Kinkony Res., Makary village	16.1334S, 45.9578E	19	04/12/09	MK878874
	NHRS-JLKB000010740	JB194	MAD09-28	Mahavavy Kinkony Res., Mitsinjo	16.0578S, 5.8059E	22	05/12/09	MK878868
	NHRS-JLKB000010741	JB189	MAD09-30	Mahavavy Kinkony RS, Mitsinjo	16.0565S, 45.7637E	55	05/12/09	MK878867
	NHRS-JLKB000065749	JB809	MAD09-24	Mahavavy Kinkony RS, Makary village	16.1465S, 45.9493E	9	04/12/09	MK878861
*C. kely* sp. nov.	NHRS-JLKB000010890		TR18L14	Ambohidray Res., Andriambe	18.6132S, 8.3259E	1044	23/05/18	MK878839
	NHRS-JLKB000065738		TR18L04	Ambohidray Res., Andriambe	18.6132S, 48.3262E	1044	07/04/18	MK878842
	NHRS-JLKB000065739		TR18L04	Ambohidray Res., Andriambe	18.6132S, 48.3262E	1044	07/04/18	MK878843
	NHRS-JLKB000065740		TR18L07	Ambohidray Res., Andriambe	18.6131S, 48.3257E	1046	07/04/18	MK878844
* C. befasicus *	NHRS-JLKB000010860	JB204	MAD09-74	btw Morafenobe-Ambohijanahary Res.	18.1909S, 45.1999E	290	19/12/09	MK878825
*Copelatus* sp. ♀ (Bemaraha)	CASENT-8135000		BLF4432	Tsingy de Bemaraha NP	19.1323S, 4.8147E	150	16/11/01	MK878860
* C. distinguendus *	BMNH-670601	007:E07	P27MD31	Ranomafana NP	21.2359S, 47.3963E	1123	06/12/04	HQ381662
	BMNH-729896		P30MD33	Sahatsiho Ambohimanjaka	20.2388S, 47.1002E	1442	08/12/04	HQ381870
	BMNH-792954		P39EM08	Andringitra NP	22.1043S, 6.9207E	1420	09/05/06	HQ382583
	BMNH-792955		P39EM08	Andringitra NP	22.1043S, 46.9207E	1420	09/05/06	HQ382584
	BMNH-792956		P39EM08	Andringitra NP	22.1043S, 46.9207E	1420	09/05/06	HQ382585
	BMNH-792962		P36C	RN7, Col de Tapias	20.7729S, 47.1792E	1717	06/05/06	HQ382591
	BMNH-792963		P36C	RN7, Col de Tapias	20.7729S, 47.1792E	1717	06/05/06	HQ382592
	BMNH-792964		P36C	RN7, Col de Tapias	20.7729S, 7.1792E	1717	06/05/06	HQ382593
	BMNH-792976		P30MD33	Sahatsiho Ambohimanjaka	20.2388S, 47.1002E	1442	08/12/04	HQ382604
	BMNH-792977		P30MD33	Sahatsiho Ambohimanjaka	20.2388S, 47.1002E	1442	08/12/04	HQ382605
	BMNH-792978		P30MD33	Sahatsiho Ambohimanjaka	20.2388S, 47.1002E	1442	08/12/04	HQ382606
	BMNH-792979		P30MD33	Sahatsiho Ambohimanjaka	20.2388S, 47.1002E	1442	08/12/04	HQ382607
	BMNH-792980		P30MD33	Sahatsiho Ambohimanjaka	20.2388S, 7.1002E	1442	08/12/04	HQ382608
	NHRS-JLKB000010627		MAD14-81	RN2, Betsabora river	18.9247S, 48.1828E	900	24/11/14	MK878834
	NHRS-JLKB000010670		MAD16-47	Manjakatompo Ankaratra Res., Ankafotra Mtn.	19.3375S, 47.2453E	2466	18/09/16	MK878835
* C. insuetus *	NHRS-JLKB000010609		MAD14-81	RN2, Betsabora river	18.9247S, 8.1828E	900	24/11/14	MK878836
	NHRS-JLKB000010875		MAD18-91	Zahamena NP, Sect. Antanandava	17.5225S, 48.7227E	1040	08/03/18	MK878841
	NHRS-JLKB000065745		MAD11-26	Analamazaotra NP	18.9357S, 48.4174E	930	08/11/11	MK878840
	NHRS-JLKB000065702		MAD14-18	Analamazaotra NP	18.9357S, 48.4174E	930	27/11/14	MK878837
	BMNH-797895		P60BI15	Zahamena NP, Sect. Antanandava	17.52S, 48.721E	1075	31/11/06	HQ382927
*C. safiotra* sp. nov.	NHRS-JLKB000010589		MAD11-37	Mantadia NP	18.8340S, 8.4378E	1000	11/11/11	MK878829
	NHRS-JLKB000010595		MAD14-70	Anjanaharibe Sud res.	14.7414S, 49.4975E	910	16/11/14	MK878833
	NHRS-JLKB000010846		MAD14-04	Ranomafana NP	21.2395S, 47.3947E	1130	02/11/14	MK878830
	NHRS-JLKB000010847		MAD12-03	Isalo NP, Canyon des Makis	22.4866S, 45.3797E	700	13/11/12	MK878831
	NHRS-JLKB000010848		MAD12-03	Isalo NP, Canyon des Makis	22.4866S, 45.3797E	700	13/11/12	MK878832
	NHRS-JLKB000065735		MAD13-55	Ivohibe RS	22.4567S, 46.9563E	874	09/12/13	MK878845
	NHRS-JLKB000065736		MAD13-55	Ivohibe RS	22.4567S, 46.9563E	874	09/12/13	MK878846
* C. mahajanga *	NHRS-JLKB000010554		MAD14-81	RN2, Betsabora river	18.9247S, 48.1828E	900	24/11/14	MK878826
	NHRS-JLKB000010596		MAD14-81	RN2, Betsabora river	18.9247S, 48.1828E	900	24/11/14	MK878827
	NHRS-JLKB000010723	JB190	MAD09-58	Tsingy de Bemaraha NP, Bekopaka	19.0357S, 44.7751E	66	15/12/09	MK878875
	NHRS-JLKB000010724	JB195	MAD09-33	Mahavavy Kinkony Res., Anjohibe	16.0133S, 46.0038E	24	06/12/09	MK878876
	NHRS-JLKB000065747	JB802	MAD09-25	Mahavavy Kinkony Res., Makary village	16.1334S, 45.9578E	19	04/12/09	MK878828
*C. ankaratra* sp. nov.	NHRS-JLKB000010614		MJK12-13	Manjakatompo Ankaratra Res., Anosiarivo	19.3449S, 47.3041E	2073	24/01/12	MK878851
	NHRS-JLKB000010652		MAD16-11	Manjakatompo Ankaratra Res., Tsiafajavona Mtn.	19.3516S, 47.2428E	2597	07/02/16	MK878847
	NHRS-JLKB000010864		MAD16-11	Manjakatompo Ankaratra Res., Tsiafajavona Mtn.	19.3516S, 47.2428E	2597	07/02/16	MK878848
	NHRS-JLKB000010866		MAD16-11	Manjakatompo Ankaratra Res., Tsiafajavona Mtn.	19.3516S, 47.2428E	2597	07/02/16	MK878849
	NHRS-JLKB000065704		MAD16-11	Manjakatompo Ankaratra Res., Tsiafajavona Mtn.	19.3516S, 47.2428E	2597	07/02/16	MK878850
* C. pulchellus *	NHRS-JLKB000065733	JB808	MAD09-25	Mahavavy Kinkony RS, Makary village	16.1334S, 45.9578E	19	04/12/09	MK878859
	NHRS-JLKB000065703		MAD14-14	Analamazaotra NP	18.9355S, 48.4166E	930	27/11/14	MK878856
	NHRS-JLKB000065737		MAD14-14	Analamazaotra NP	18.9355S, 48.4166E	930	27/11/14	MK878857
*Copelatus* sp. ♀ (Andasibe)	NHRS-JLKB000065698			Analamazaotra NP, Andasibe	18.94S, 48.43E	938	17/01/15	MK878858
*Copelatus ? insuetus* ♀ (Ankaraf.)	NHRS-JLKB000010694	JB206	MAD09-07	Ankarafantsika NP, Ampijoroa	16.3034S, 46.8107E	74	29/11/09	MK878877
	NHRS-JLKB000010781	JB205	MAD09-03	Ankarafantsika NP, Ampijoroa	16.3035S, 46.8107E	87	29/11/09	MK878838
*Copelatus* sp. ♀ (Ivohibe)	NHRS-JLKB000010856		MAD13-61	Ivohibe RS, Andranovory	22.4751S, 46.9559E	1106	10/12/13	MK878854
	NHRS-JLKB000065734		MAD13-61	Ivohibe RS, Andranovory	22.4751S, 46.9559E	1106	10/12/13	MK878855
*Copelatus* sp. ♀ (N Toam.)	NHRS-JLKB000010779		MAD11-52	RN5, Ivoloina	18.0649S, 49.3786E	0	15/11/15	MK878852
	NHRS-JLKB000010811		MAD17-12	Analalava Res., Analalava forest	17.7106S, 49.4500E	39	09/03/17	MK878853
*C. pseudostriatus* sp. nov.	BMNH-672727	027:A05	P32	Tsaratanana massif, Mangindrano	14.1824N, 48.9448E	1700	20/12/04	HQ381767
	BMNH-672728	027:A06	P32	Tsaratanana massif, Mangindrano	14.1824N, 48.9448E	1700	20/12/04	HQ381768
	BMNH-672729	027:A07	P32	Tsaratanana massif, Mangindrano	14.1824N, 48.9448E	1700	20/12/04	HQ381769

### Phylogenetic analysis

We first performed a Bayesian phylogenetic analysis to produce a CO1-genetree. As the taxon selection here is not aimed at producing a phylogeny, but to interprete genetic variation in light of morphological delimitations of a diagnosable set of species in a certain geographic region (Madagascar), the genetree was artificially rooted using *Copelatus befasicus*. We used PARTITIONFINDER Ver. 2.1.1 (Lanfear et al. 2016) to infer a suitable partitioning scheme (three codon-position specific partitions defined as input) and for each partition, a suitable model. The choice of models was limited to those available in MrBAYES, and selection was based on AICc scores. We ran 10M generations in MrBAYES Ver. 3.2.6 (Ronquist et al. 2012) to infer a non-clock CO1 genetree under selected partitioning scheme and models. Two runs, each with one cold and three heated chains were sampled every 1000^th^ generation. A majority-rule consensus tree was calculated from both runs after removal of 25% as burn-in from each.

To explicitly compare our morphological delimitations with a single-locus species delimitation method we implemented the GMYC-method (Fujisawa and Barraclough 2013). An ultrametric strict clock tree was calculated with BEAST Ver. 1.8.4 (Drummond et al. 2012) using the same partitioning and model scheme selected by PARTITIONFINDER above. Rooting was here done with the strict clock model. 2×50M generations were sampled every 1000^th^ generation, and treeannotator was used to calculate a maximum clade credibility tree with median node heights, removing 10% as burn-in from each run. The GMYC analysis was carried out in R using the SPLITS package (Ezard et al. 2009) on this tree, under the single-threshold method. Convergence and mixing of MCMC runs from MrBAYES and BEAST were checked by the statistics provided by respective programmes and with TRACER Ver. 1.7.1 (Rambaut et al. 2018).

## Results

### Molecular results

Our amplification of CO1 was successful for 53 samples which, together with sequences downloaded from GenBank, gave 77 terminals (Table 1). Alignment length was 825 bp and was gap-free but some sequences were unreadable near primer regions and therefore slightly shorter. The amplification of two shorter fragments from the degraded DNA originating from the two dry-preserved specimens was partially successful and gave 311 bp (CASENT-8135000) and 447 bp (NHRS-JLKB000065698) respectively and enabled inclusion in the phylogenetic analyses.

**Figure 2.**
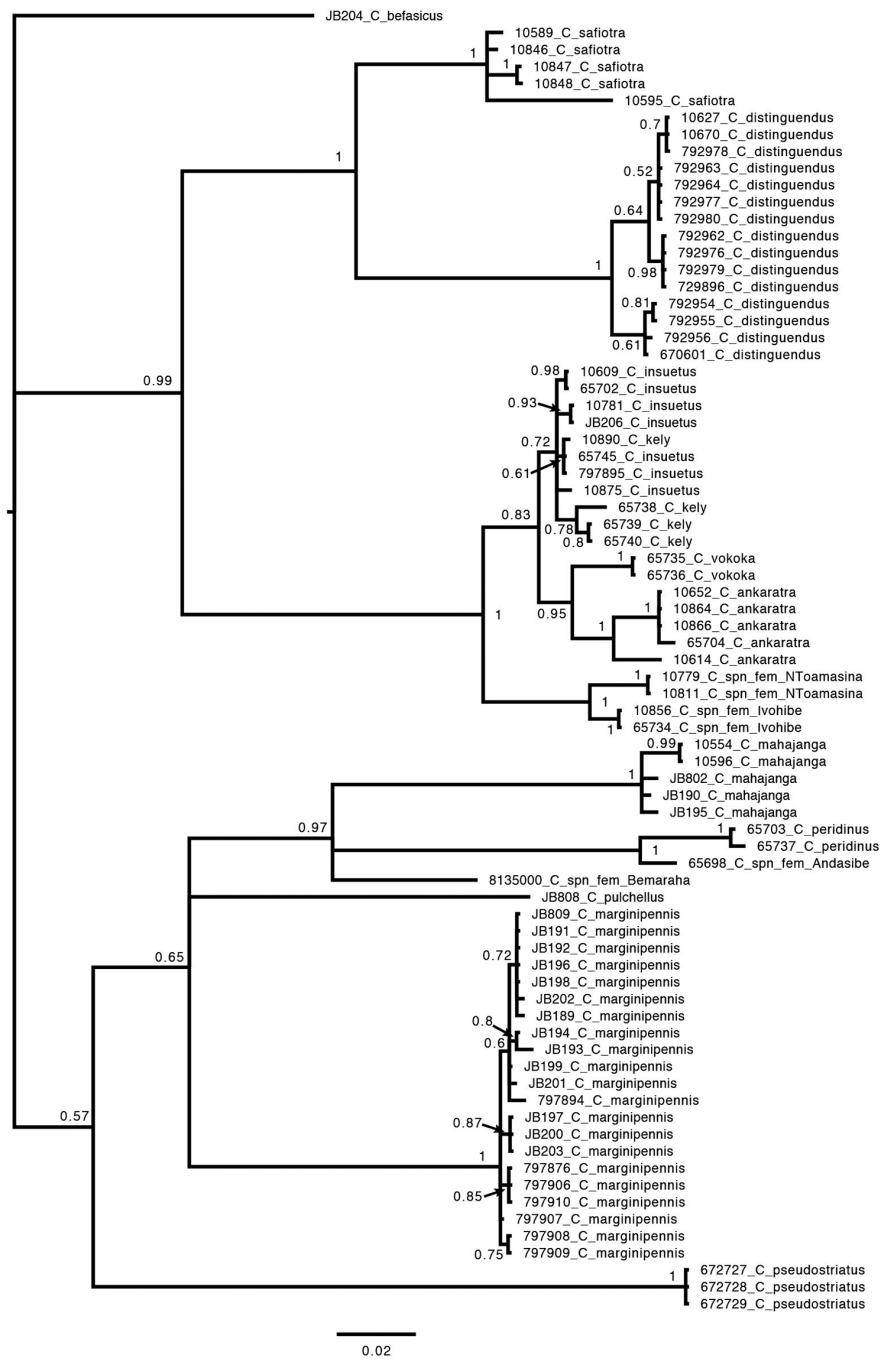
Majority-rule consensus tree from Bayesian analysis of CO1. Values next to nodes indicate posterior probabilities.

Specimens of morphologically identified species all clustered as monophyletic in the Bayesian analysis except *C. insuetus* and *C. kely* sp. nov. (Fig. 2). These are part of a closely related group of species we will refer to as the *Copelatus insuetus* complex with interspecific genetic divergences ranging between 0.1–7.1%, but several not more than 2–3% (Table 2). Morphological analysis was inconclusive for some female specimens or populations in this group and these are discussed under respective species.

**Table 2.** Uncorrected P genetic distances between closely related species or populations in the group near *Copelatus insuetus*. Numbers in parenthesis are genetic distances calculated under a Kimura-2-parameter model. NA = Not Applicable because of single sample in category.

	* C. insuetus *	*C. insuetus*? (Ankaraf.)	* C. kely *	*C. ankaratra* (peak)	* C. ankaratra *	* C. vokoka *	*C.* sp. female (Ivohibe)	*C.* sp. female (N. Toam.)
*** C. insuetus ***	0–0.007							
	(0–0.007)							
***C. insuetus***?	0.005–0.009	0						
(Ankaraf.)	(0.005–0.009)	(0)						
*** C. kely ***	0.001–0.017	0.006–0.011	0–0.017					
	(0.001–0.017)	(0.006–0.011)	(0–0.017)					
*** C. ankaratra ***	0.032–0.036	0.028–0.031	0.028–0.038	0–0.004				
(peak)	(0.033–0.038)	(0.029–0.032)	(0.029–0.039)	(0–0.004)				
*** C. ankaratra ***	0.030–0.039	0.029–0.031	0.028–0.038	0.023–0.025	NA			
	(0.031–0.040)	(0.030–0.032)	(0.029–0.040)	(0.023–0.025)	NA			
*** C. vokoka ***	0.026–0.029	0.028	0.024–0.029	0.033–0.035	0.033	0		
	(0.026–0.030)	(0.028–0.029)	(0.024–0.030)	(0.034–0.036)	(0.034)	(0)		
***C.* sp. female**	0.046–0.048	0.044–0.045	0.048–0.050	0.059–0.060	0,061	0.051–0.052	0	
(Ivohibe)	(0.048–0.049)	(0.045–0.047)	(0.050–0.053)	(0.062–0.064)	(0.065)	(0.054)	(0)	
***C.* sp. female**	0.051–0.054	0.047–0.049	0.052–0.053	0.063–0.066	0.066	0.058–0.059	0.023–0.024	0
(N. Toam.)	(0.053–0.057)	(0.049–0.051)	(0.054–0.056)	(0.067–0.071)	(0.070–0.071)	(0.061–0.062)	(0.023–0.024)	(0)

**Figure 3.**
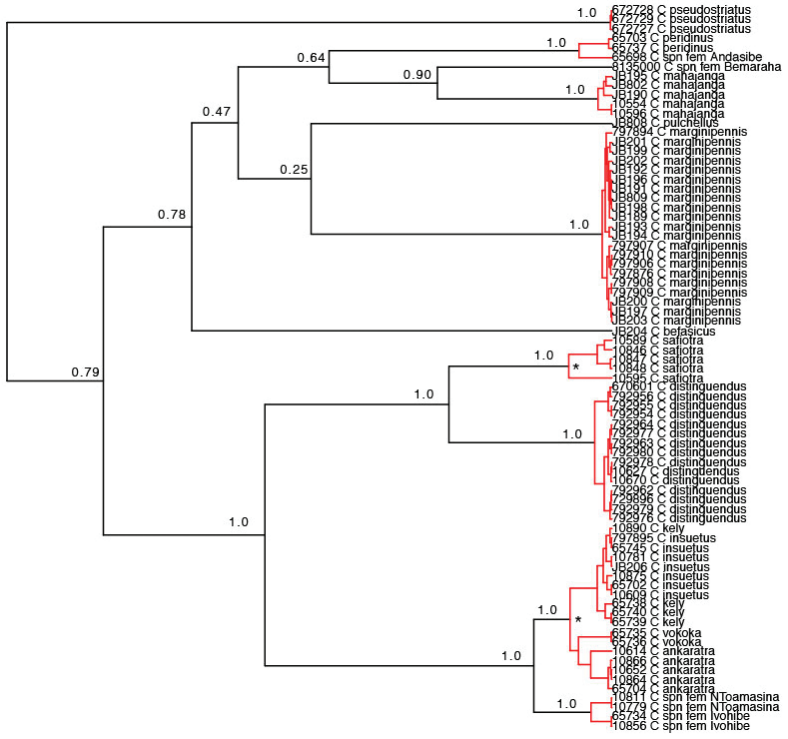
Result of the single-locus GMYC species delimitation using an ultrametric CO1 genetree from BEAST. Black branches indicate interspecific divergences, red branches represent intraspecific coalescence events. Values above interspecific nodes indicate posterior probability from the Beast analysis under a strict clock model. The * indicates two nodes of further splitting from the GMYC analysis that is within the confidence interval of 2Log likelihood units from the optimal solution.

The GMYC species delimitation analysis of the strict clock tree resulted in 11 separate evolutionary units that were largely but not entirely consistent with our morphological delimitation (Fig. 3). Especially, the *Copelatus insuetus* complex of four morphologically delimited species where merged into one unit in the GMYC analysis. But in contrast the non-named females that we could not identify morphologically were separated in the GMYC analysis and these very likely represent one or two new species of which we have yet to discover the male. In no case, had the GMYC analysis split groups of individuals that were morphologically considered the same species. The approximative 2log-likelihood confidence interval included between 5–13 units and in the case (13) closest to the morphological delimitations, the *Copelatus insuetus* complex was divided into two species, but also the northernmost *Copelatus safiotra* sp. nov. specimen was separated from geographically more southern populations. The dry-preserved female from Bemaraha (CASENT-8135000) undoubtedly represents a fourth Malagasy species from the *Copelatus pulchellus* complex. However, the female from Andasibe (NHRS-JLKB000065698) was merged with *Copelatus peridinus*, despite a genetic distance of approximately 3%, and this is discussed below.

### Identification key

Key to *Copelatus* species of Madagascar with fewer than ten discal and one submarginal elytral striae. Note that for some species males are necessary.

**Table:** 

1	Impressed elytral striae absent (rows of points may be present) (Fig. 7A, B)	**2** (hydroporoides group)
–	Impressed elytral striae present (Figs 7D, 8–10)	**3**
2	Body length less than 4 mm (Fig. 7B); penis shape unknown (only 1 female known)	*** C. baculiformis ***
–	Body length between 5.7 and 6.6 mm (Fig. 7A); penis in lateral view with non-even curvature creating two gentle angles (Fig. 4A)	*** C. peridinus ***
3	Elytra with five discal but no submarginal stria; dorsal surface reddish brown with a lighter elytral base; first elytra stria abbreviated and only present in posterior third; body length 4.1–4.2 mm (Fig. 10D)	***C. befasicus*** (longicornis group)
–	Elytra with six discal and one submarginal stria (Figs 7D, 8, 9, 10A–C); other characters the same or different	**4** (irinus group)
4	First, third, and fifth elytral striae distinctly abbreviated anteriorly; dorsal colouration largely black (Fig. 7D); penis distinctive in lateral view resembling a “pumpjack” with a long sub-erect neck and a downturned apex (Fig. 4B)	*** C. distinguendus ***
–	Third and fifth elytral striae not abbreviated anteriorly; first elytral stria abbreviated or not; dorsal colouration rarely mostly black; penis shape variable	**5**
5	Body shape more broadly oval (Fig. 8)	**6**
–	Body shape narrow, elongate, subparalell (Figs 9, 10)	**8**
6	Elytra without a transverse testaceous band at base, black except laterally and posteriorly (Fig. 8D); penis in lateral view rather evenly curved with neither dorsal knob nor ventral invagination; penis in ventral view non-expanded apically and with apex left-curved (Fig. 5C)	*** C. pulchellus ***
–	Elytra with basal testaceous transverse band; penis apex in ventral view right-curved	**7**
7	Body broadly oblong; first elytral stria often longer and basal testaceous elytral band narrower but both characters overlapping; penis in lateral view with dorsal knob and ventral invagination; penis in ventral view not broadly expanded apically, but with a small tooth (Fig. 5A)	*** C. marginipennis ***
–	Body more narrowly oblong and attenuating posteriorly; first elytral stria often shorter and elytra with a broader testaceous transverse band at base; penis in ventral view bisinuate and widened at apex (Fig. 5B)	*** C. mahajanga ***
8	Elytra with irregular traces of intermediate striae or “pseudostriae” between first and second stria and between second and third stria; body length larger, 5.3 to 5.6 mm (Fig. 10B); penis curvature in lateral view rather weak (Fig. 4C)	***C. pseudostriatus* sp. nov**.
–	Elytra without pseudostriae in elytral intervals; body length smaller, less than 5.3 mm; Penis curvature in lateral view stronger	**9**
9	Body subparallell and head with a broad interocular distance; pronotum without strioles (Fig. 10C); penis in lateral view with a distinct expansion subapically followed by a narrow apical blade (Fig. 6E)	***C. safiotra* sp. nov.**
–	Body more attenuating anteriorly and posteriorly and interocular distance narrower; pronotum with strioles posterolaterally; penis slender, without subapical expansion in lateral view	**10**
10	Dark colouration dorsally, with narrow testaceous band at base of elytra and testaceous, sometimes strongly contrasting, anterolateral pronotal corners; head distinctly infuscated (Fig. 10A); base of penis in posteroventral view distinctly angled; apex in lateral view without a dorsal ridge crossing posterior inner outline (Fig. 6D)	***C. ankaratra* sp. nov.**
–	Colouration usually lighter brown, testaceous band at base of elytra variable; base of penis in posteroventral view not angled; apex in lateral view with or without a dorsal ridge crossing posterior inner outline	**11**
11	Body length smaller, 3.8 to 4.3 mm (Fig. 9C); penis apex in lateral view without a dorsal ridge crossing posterior inner outline (Fig. 6B)	***C. kely* sp. nov.**
–	Body length larger, 3.9 to 5.0 mm; penis in lateral view with a dorsal ridge crossing posterior inner outline near apex	**12**
12	Elytra usually with a more narrow basal testaceous band; penis in lateral view with distinct shoulder definition interrupting an evenly curved outer outline (Fig. 6A); penis on ventral to right-lateral side with coarse longitudinal sulci (Fig. 6A)	*** C. insuetus ***
–	Elytra often with broader basal testaceous band; penis in lateral view evenly curved without a distinct shoulder definition (Fig. 6C); penis on ventral to right-lateral side with only weak longitudinal microsculpture (Fig. 6C)	***C. vokoka* sp. nov.**

**Figure 4.**
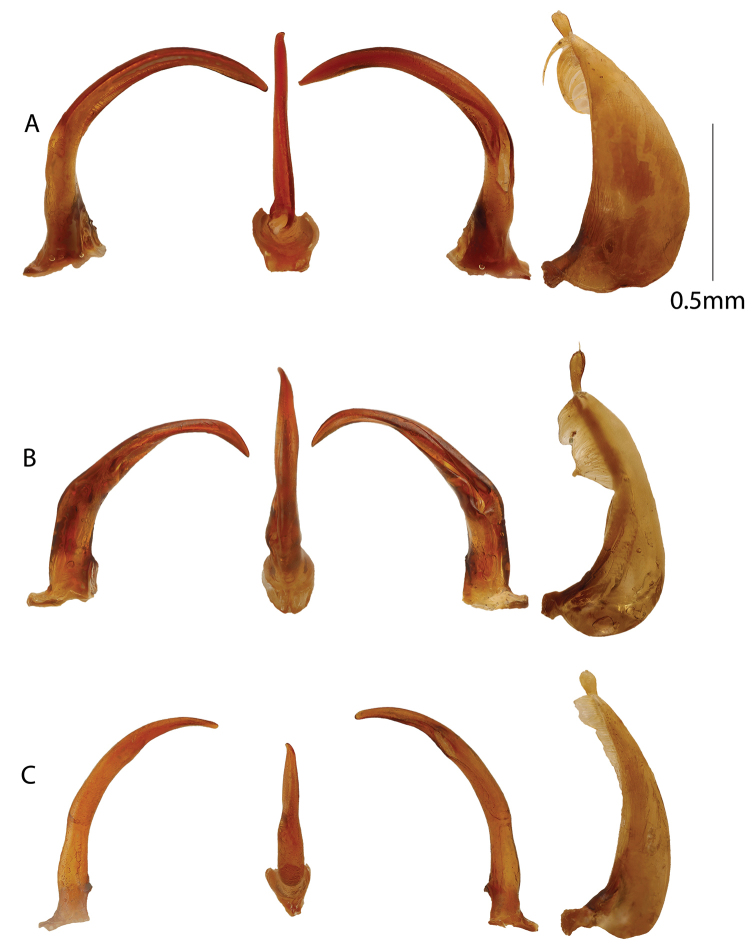
Male genitalia. From left to right, aedeagus in right lateral, ventral, left lateral views and left paramere. **A***Copelatus peridinus***B***Copelatus distinguendus***C***Copelatus pseudostriatus* sp. nov.

**Figure 5.**
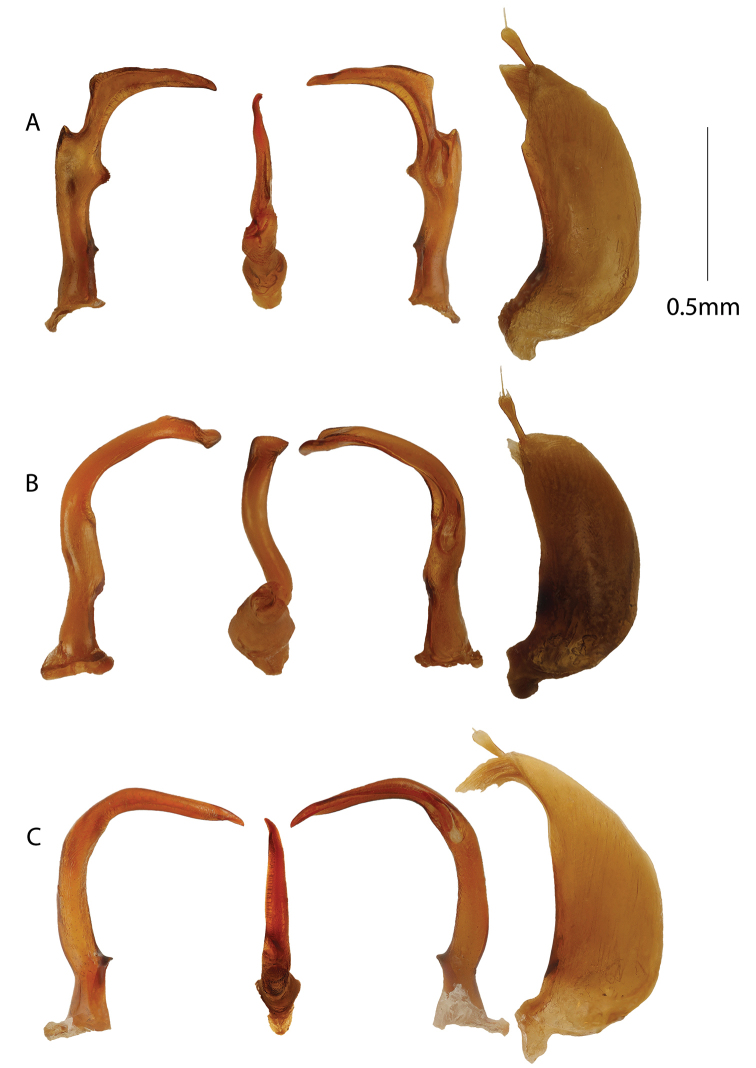
Male genitalia. From left to right, aedeagus in right lateral, ventral, left lateral views and left paramere. **A***Copelatus marginipennis***B***Copelatus mahajanga***C***Copelatus pulchellus*.

**Figure 6.**
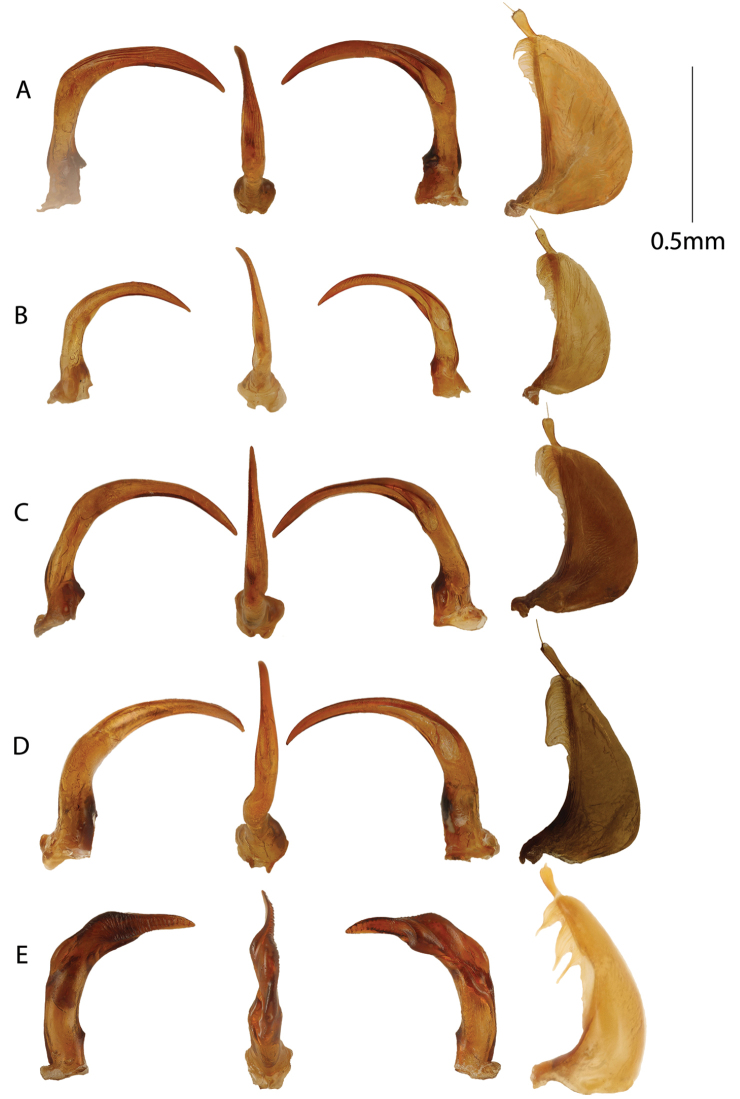
Male genitalia. From left to right, aedeagus in right lateral, ventral, left lateral views and left paramere. **A***Copelatus insuetus***B***Copelatus kely* sp. nov. **C***Copelatus vokoka* sp. nov. **D***Copelatus ankaratra* sp. nov. **E***Copelatus safiotra* sp. nov.

## Taxonomy

The following three taxa were described as *Copelatus* species from Madagascar and were listed as such by Rocchi (1991):

### Exocelina subjecta

Taxon classificationAnimaliaColeopteraDytiscidae

(Sharp, 1882)

0A694D306A4456EB8ADB140766C419B7

Copelatus subjectus Sharp, 1882: 568.
Copelatus bilunatus Guignot, 1955: 73; TL: Madagascar [mislabelled, likely New Caledonia; see Balke et al. (2014)]

#### Type locality.

New Caledonia.

#### Remarks.

*Copelatus bilunatus* Guignot, 1955 is a synonym of *Exocelina subjecta* (Sharp, 1882) following Balke et al. (2014). *Copelatus bilunatus* was described by Guignot (1955a) from Zimmermann’s collection based on a “♀ unique” from “Madagascar” without any further locality data. The species was assigned to *Copelatus* based on the presence of complete metacoxal lines and placed in the *Copelatus hydroporoides* species group, as it lacked impressed elytral striae. Balke et al. (2014) concluded that the specimen is identical to *Exocelina subjecta* (Sharp, 1882), a common species on New Caledonia, and synoymised the two assuming the specimen was mislabelled. Apparently the holotype is a male in contrast to what is stated in the original description. Balke et al. (2014) document the presence of a stout spine-like setae on the anterodistal angle of protarsomere IV, which is characteristic of the Copelatinae genus *Exocelina* (but also of Malagasy *Madaglymbus*). We have not examined the type ourselves but it is clear that in any case it is not a *Copelatus* species.

### Madaglymbus apicalis

Taxon classificationAnimaliaColeopteraDytiscidae

(Fairmaire, 1898) comb. nov.

7B3FDEB746D75239A167247A824B06E0

Copelatus apicalis Fairmaire, 1898: 465.

#### Type locality.

Madagascar, Suberbieville [= Maevatanana].

#### Type material examined.

**Mahajanga. Betsiboka: Maevatanana**: -ST ♂ (GP) (MNHN, “coll. Régimbart”): // Data in NHRS | JLKB | 000030208 // Madag. Perrier | // Type [red writing] // Museum Paris | Coll. Régimbart | apicalis // *Copelatus* | *apicalis* n. sp. //

#### Remarks.

*Copelatus apicalis* was described by Fairmaire (1898) from Suberbieville [=Maevatanana]. It is classified in the genus *Copelatus* and currently placed in the *Copelatus hydroporoides* species group (Guignot 1961; Nilsson et al. 1997; Nilsson 2001; Nilsson and Hájek 2018). We have studied a male syntype of this species which was not found by Guignot (1961: note 638) and conclude that is not a *Copelatus*. The syntype male has a stout spine-like setae on a protruding anterodistal corner of protarsomere IV which places it in *Madaglymbus* (Shaverdo et al. 2008) and we hereby transfer it to that genus: *Madaglymbus apicalis* (Fairmaire, 1898) comb. nov.; in fact Guéorguiev (1968: 32) suggested it might belong to *Aglymbus* “Peut-être c’est une *Aglymbus*?” [Eng. translation “Maybe it is an *Aglymbus*”], which was correct since this was before *Madaglymbus* was erected for the Madagascar species of *Aglymbus* (Shaverdo et al. 2008).

### Madaglymbus unguicularis

Taxon classificationAnimaliaColeopteraDytiscidae

(Régimbart, 1903) comb. nov.

45AB55AB28805B229F01D47FDED687E7

Copelatus unguicularis Régimbart, 1903:19.

#### Type locality.

Madagascar, Suberbieville [= Maevatanana].

#### Type material examined.

**Mahajanga. Betsiboka: Maevatanana**: -HT ♂ (GP) (MNHN, “coll. Régimbart”): // Data in NHRS | JLKB | 000030225 // Suberbieville | Madag. Perier // Type [red label] // MUSEUM PARIS | Coll. Maurice Régimbart | 1908 // *C. unguicularis* | Type // *unguicularis* Rég. //

#### Remarks.

*Copelatus unguicularis* was described by Régimbart (1903), based on a single male from Suberbieville [= Maevatanana]. It was classified by Régimbart in the fifth species group of Sharp (1882) (the *consors* species group sensu Guignot 1961) based on ten discal but no submarginal elytral stria where it is still classified today (Guignot 1961; Guéorguiev 1968; Nilsson et al. 1997; Nilsson 2001; Nilsson and Hájek 2018). We have examined the male holotype and like the type of *Copelatus apicalis*, it has a stout spine-like setae on a protruding anterodistal corner of protarsomere IV and is not a *Copelatus*. In addition, the elytral striae are very irregular, more like very elongate and deep strioles and possibly not homologous to the regular impressed striae in *Copelatus*. We here transfer it to *Madaglymbus*: *Madaglymbus unguicularis* (Régimbart, 1903) comb. nov.

### The *Copelatus hydroporoides* group

This group is defined by the lack of elytral striae (Sharp 1882; Guignot 1961; Guéorguiev 1968). It is certainly not a natural group. A number of species formerly placed in this group has lately been moved into other genera. Perhaps it still remains useful as a “trash can” for *Copelatus* species lacking elytral striae, but it is very probably not monophyletic and likely still mixed with some copelatine species that should be transferred to other genera.

#### Copelatus baculiformis

Taxon classificationAnimaliaColeopteraDytiscidae

Guignot, 1955

0DA77899943B5226BBA57EB36328B122

Fig. 7B

Copelatus baculiformis Guignot, 1955b: 193.

##### Type locality.

Madagascar, Massif Ankaratra, Manjakatompo, alt. 1700–1800 m.

##### Type information from original description.

based on a single female specimen (holotype), collected December 1951 by R. Benoist.

##### Type material studied.

**Antananarivo. Vakinankaratra: Ambatolampy**: -HT ♀ (MNHN “coll. Guignot”): // Data in NHRS | JLKB | 000030226 // Madagascar: Massif An- | karatra 1700/1800 Man- | jakatompo XII-51 Benoist // Type [red label] // Guignot det., 1954 | *Copelatus* | *baculiformis* n. sp. | Type ♀ //

##### Diagnosis.

**Figure 7.**
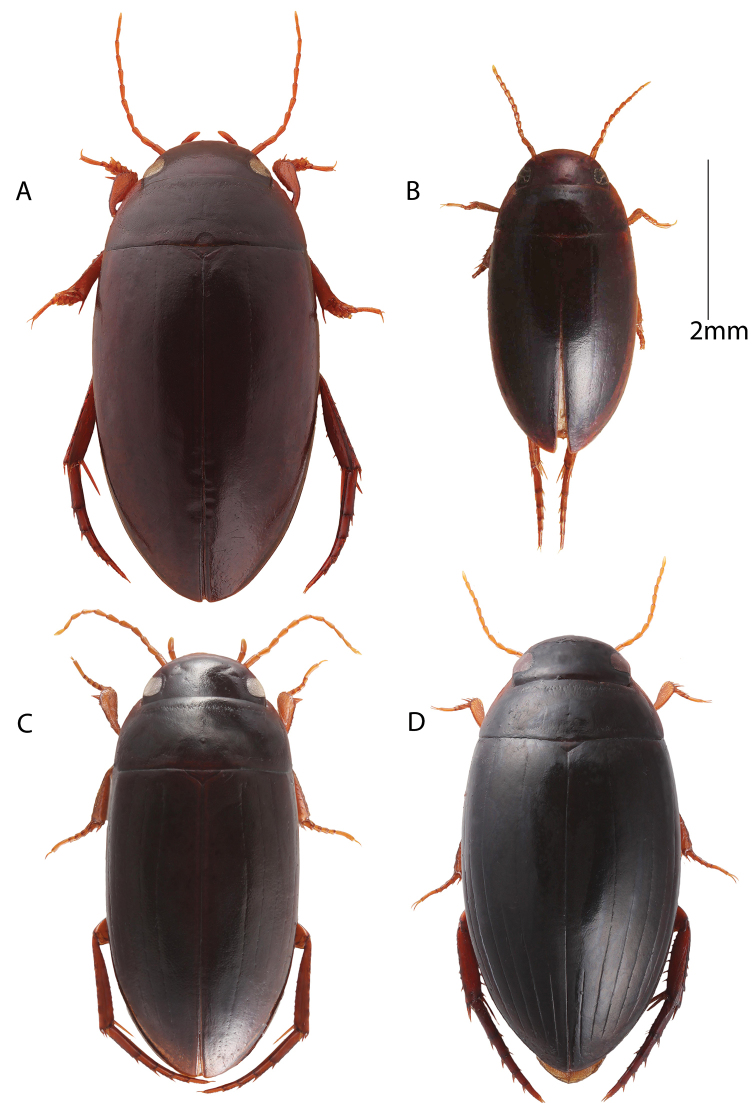
Habitus, dorsal view. **A** Male: *Copelatus peridinus***B** Female: *Copelatus baculiformis***C** Female: *Copelatus* sp. 2 (Andasibe) **D** Female: *Copelatus distinguendus*.

Small size (4 mm). Elytra uniformly dark brown ferrugineous, without a basal testaceous area (Fig. 7B) which separates the species from small species of *Madaglymbus*. The absence of deep impressed elytral striae (remnants of four elytral striae present) separates this species from all other *Copelatus* of Madagascar except *Copelatus peridinus*, a larger species (5.7–6.6 mm).

##### Description.

(based on holotype ♀):

Body length 4 mm. Body shape elongate oval and dark brown to blackish ferrugineous. Head uniformly dark brown ferrugineous to slightly darker posteriorly inside eyes, with thin sparse punctation. Pronotum dark brown ferrugineous, same colour medially and laterally but darker along anterior and posterior third. Disc of pronotum less densely punctuated, posterolateral corners with dense superficial strioles. The entire dorsal surface covered with a microsculpture. Elytra uniformly coloured in same dark brown to blackish ferrugineous colour as anterior and posterior parts of pronotum (Fig. 7B). Impressed striae absent but four elytral rows of impressed points present. Innermost, a presumed first row completely lacking, row 2, 3, and 4 visible from base but row 3 less distinct than 2 and 4. Row 5 very vague and only visible posteriorly. Elytra densely covered with punctures which laterally of approximately the third row of points is replaced by strioles (probably only in the female). Appendages testaceous.

Ventral side testaceous to weakly infuscated. Prosternal process carinate also onto apical process. Lateral parts of metaventrite (“metasternal wings”) rather broad. Metacoxal lines anteriorly diverging and abbreviated before metaventral margin. Metacoxa with fine and long longitudinal strioles, continuing onto abdominal ventrites, and with 6–7 transverse “wrinkles” anterolaterally.

Male: unknown.

##### Distribution.

Madagascar, central highlands, only known from type locality Manjakatompo, Ankaratra Massif (Fig. 11A).

##### Habitat and ecology.

Unknown, but according to original description collected at at an altitude of 1700–1800 m. See Hjalmarsson et al. (2013) for a description of the locality Manjakatompo and its conservation priority.

##### Comments.

No other specimen than the female holotype is known of this species and it is a bit of a “mystery species”. We have conducted fieldwork at the type locality Manjakatompo multiple times (2011, 2012, 2014, and 2016) but never found any specimens resembling this species. The species belongs in the *hydroporoides* species group of *Copelatus*, but two other Malagasy species placed in this group have turned out to be misidentified *Madaglymbus* or *Exocelina* (see above). *Copelatus baculiformis* was described by Guignot the same year (1955) that he described *C. bilunatus*, considered mislabeled (Balke et al. 2014) from the Zimmermann collection. It is certainly possible that also *C. baculiformis* is based on a mislabeled specimen, but in contrast to *C. bilunatus* the type locality data is more exact; Massif Ankaratra (mountain), Manjakatompo (locality), 1700–1800 m (altitude in meters), XII-51 (collecting month and year), and R. Benoist (collector), speaking against such a mistake. Based on general external morphology, body shape and lack of striae the species resembles *C. peridinus*. It is much smaller and therefore likely not conspecific, but they could be closely related, as well as with the female sequenced from Andasibe (NHRS-JLKB000065698).

#### Copelatus peridinus

Taxon classificationAnimaliaColeopteraDytiscidae

Guignot, 1955

25AF5D96C7285A06B37C8F9CC93796E0

Figs 4A , 7A

Copelatus peridinus Guignot, 1955c: 188.
Copelatus seydeli Guignot, 1958: 107; TL: Elisabethville, Zaire [DR Congo, Haut-Katanga, Lubumbashi].

##### Type locality.

Elisabethville, Zaire [DR Congo, Haut-Katanga, Lubumbashi].

##### Type material studied.

-PT ♀ (MNHN, “Coll. Guignot”): // Data in NHRS | JLKB | 000030317 // Allotype [red label] // [female symbol] // Elisabethville | XI. 1951 // *Copelatus* | *peridinus* | Allotype ♀ //

##### Additional material studied.

-1♂(GP) (MNHN, “Coll. Guignot”): // Data in NHRS | JLKB | 000030318 // Congo Belge Lac | Edouard: Vitshumbi | U.V. 27.XI.1953 | 3091 // **Antananarivo. Analamanga: Antananarivo**: -3♂(GP), 3♀ (MNHN, “Coll. Legros Magasin”): // Data in NHRS | JLKB | 000030319–24 // MADAGASCAR | TANANARIVE | BETONGOLO | 2 XII 1946 // Piège | lumineux // Museum Paris | 1983 | Coll. Cl. Legros // **Toamasina. Alaotra Mangoro: Moramanga**: -1♂(GP), 2♀ (MNHN, “Coll. Paulian”): // Data in NHRS | JLKB | 000030316, 30325–6 // Madagascar Est | P.K.57-Rte d’Anosibe | Moramanga | II.58 R. Vieu // -1♂(GP), 1♀ (NMPC, “Coll. J. Hájek”): // Data in NHRS | JLKB | 000030327, 30328 // Madagascar | Lokato, near | Andasibe Mantadia NP | M. Tryzna leg., 9–10.i.2007 // coll. Jiri HÁJEK | National Museum | Prague, Czech Republic // [“Lokato near Andasibe Mantadia NP” interpreted as the bifurcation of the road to Lakato which is near Andasibe Mantadia NP. Lakato itself is not near Andasibe Mantadia NP.] -2♂(GP), 1♀(teneral), 1♀, 1♂ (Alc. teneral) (NHRS): // NHRS-JLKB | 000010887, 65737, 65703, 10888(Alc.) // MAD: TOAM: Alaotra Mangoro: | Andasibe Mantadia NP, Analamazaotra: | 150m E of park entrance: Mad14-14 | shallow partly dried out forest pond: | 18.9355S | 48.4166E: 930m: 27.XI.2014 // Leg. J. Bergsten, R. | Bukontaite, S. Holmgren, | J.H. Randriamihaja & T. Ranarilalatiana //

##### Diagnosis.

Similar to *C. baculiformis* based on shape and colouration, but *C. peridinus* is bigger with body length between 5.7 and 6.6 mm (Fig. 7A). Elytra lacking deeply impressed striae, but elytral rows of impressed points present out of which two rows are most distinct. Penis in lateral view curved with two points at which it curves slightly more abruptly creating two gentle angles (Fig. 4A); parameres rather broad at base and evenly curved (Fig. 4A).

##### Description.

Body length 5.7–6.6 mm. Body shape elongate oval, from midpoint uniformly tapering towards a rather pointed apex. Head and pronotum both in the same dark brown to blackish ferrugineous colouration; elytra anteriorly in the same colour as pronotum, but posterior part brown ferrugineous with largely testaceous lateral margins (Fig. 7A). Head, pronotum and elytra with thin dense punctation, in addition pronotum and elytra with small and shallow punctures, and microsculptured. At posterolateral corners of pronotum, punctures almost a little joined and corrugated. Elytra lacking deeply impressed striae. Two continuous rows of punctures are most obvious and divide the elytra in three more or less equal intervals. A third and a fourth row of punctures also present, albeit less distinct and more fragmented. These occur between the two first distinct rows and between the second distinct row and the elytral margin. A fifth row suggested between the two outermost rows by a few spaced out punctures, almost unidentifiable in most Malagasy specimens seen but more distinct in specimens seen from mainland Africa. Appendages testaceous to rufus.

Ventral side dark ferrugineous. Prosternal process strongly carinate anteriorly and with a rather short process. Lateral parts of the metaventrite medium-broad. Metacoxal lines short, anteriorly diverging and abbreviated well before metaventral margin. Metacoxa with short fine strioles continuing onto abdominal ventrites.

Male: first three pro- and mesotarsomeres widened. Protibia modified, narrow at base and with an early abrupt bend, extended and broadened towards middle with a straight ventral side but angled dorsal side. Pro- and mesotarsal claws unmodified.

Penis in ventral view with apical part more or less straight and even, gently pointed at apex but with the very apex minutely twisted to the right (Fig. 4A). Penis in lateral view curved with two points (1/3^rd^ and 2/3^rd^ from base) at which it curves slightly more abruptly creating two gentle angles (Fig. 4A). Parameres rather broad at base and evenly curved and tapering towards apex (Fig. 4A).

Female: dorsal sculpture similar to male.

##### Distribution.

Likely a more widespread distribution in at least central and eastern continental Africa than the current records (Lubumbashi and Kivu in DR Congo) indicate. In Madagascar, only know from the eastern central parts: Betongolo (Antananarivo), the Analamazaotra NP, and P.K.57 Route d’Anosibe [RN23] (Fig. 11C).

##### Habitat and ecology.

A series of six specimens collected with light trap (“piège lumineux”) in the capital Antananarivo 1946, indicates flight capacity and anthropogenically disturbed habitats. All records from DR Congo are also from light trap catches (Bilardo 1982). We collected a second series of teneral specimens in a shallow forest pool with vegetation, near the entrance to the Analamazaotra NP.

##### Comments.

This species was described from Lubumbashi, DR Congo, and has not been recorded from Madagascar before. Earliest record found is from November 1946. It seems to be a dispersive good flier and often collected at light so its presence in Madagascar is therefore not surprising. However, it is not widespread in Madagascar as far as we know. In fact, the known distribution is restricted to the surroundings of the capital and east of the capital along the main national route towards Toamasina, which could suggest a recent incidental human-mediated introduction from mainland Africa.

Note that it may be that this is a species with intraspecific variation with regards to elytral striation, ranging from five puncture lines out of which two are more distinct, to five weakly impressed striae (see further discussion under *Copelatus* sp. 2 below).

### The *Copelatus longicornis* group

Sharp (1882) defines this group as those having 3, 4, or 5 discal striae on elytra but lacking a submarginal stria. There is only one species in Madagascar from this group, *Copelatus befasicus* Guignot, 1956, and it has five discal striae.

#### Copelatus befasicus

Taxon classificationAnimaliaColeopteraDytiscidae

Guignot, 1956

246290F17F4059918CAB1EF8A036B9D5

Fig. 10D

Copelatus befasicus Guignot, 1956: 79.

##### Type locality.

Madagascar, Morondava, forest south of Befasy.

##### Type information from original description.

based on an unknown number of female type specimens but holotype and paratypes are distinguished in introduction. Collected in January 1956 by R. Paulian.

##### Type material studied.

**Toliara. Menabe: Morondava**: -HT♀ (MNHN, “coll. IRSM”): // Data in NHRS | JLKB | 000030021 // Morondava | fôret sud | de Befasy | I-56 R.P // Type [red label] // INSTITUT | SCIENTIFIQUE | MADAGASCAR // Guignot det., 1956 | *Copelatus* | *befasicus* n. sp. | Type // -2PT ♀ (MNHN, “coll. Guignot”): // Data in NHRS | JLKB | 000030300-1// Morondava | fôret sud | de Befasy // I-56 R.P // Paratype [red label] // [female symbol] //

##### Additional material studied.

**Mahajanga. Melaky: Morafenobe**: -1♀ (NHRS): // NHRS-JLKB | 000010860 (JB204) // Madagascar: Mahajanga: Melaky | Btw. Morafenobe–Ambohijanahary | S18.19091; E045.19986, 290 m.a.o | 19.XII.2009 Water Net, Field# MAD09-74 | Leg: J. Bergsten, N. Jönsson, T. | Ranarilalatiana, H.J. Randriamihaja //

##### Diagnosis.

Similar to *C. insuetus* and related species in habitus by being small, elongate, and subparalell, but *C. befasicus* is distinguished from *C. insuetus* and from all other Malagasy *Copelatus* species by the presence of only five elytral striae, and without submarginal striae. In addition, the first stria is shortened, present only in the posterior third (Fig. 10D).

##### Description.

Body length 4.1–4.2 mm. Body shape elongate and subparallel, dorsal surface reddish brown with a lighter elytral base. Head and pronotum uniformly rufus brown. Head, pronotum and elytra with dense punctation. Elytra and pronotum covered with dense punctures and the whole dorsal surface with a microsculpture. Lateral sides of pronotum striolate with the widest striolate area in the posterior corners. Elytra light brown with a distinct testaceous band basally (Fig. 10D). First elytral stria shortened and present only in posterior third. Second to fifth elytral stria starting more or less at base and all striae approaching the apex of elytron, but the second and fourth a little shorter. Submarginal striae absent. Appendages testaceous.

Ventral side light brown. Metacoxa and abdominal ventrites punctate and striolate. Prosternal process rather short and medially raised, triangular in cross-section. Lateral parts of metaventrite medium broad. Metacoxal lines anteriorly diverging but rather weakly so, abbreviated well before metaventral margin. Antennae, palps and legs testaceous.

Male: unknown.

##### Distribution.

Known only from two localities in the western part of Madagascar, the deciduous forest south of Befasy, Morondava, and at one locality between Morafenobe and Beravina village (Fig. 12D).

##### Habitat and ecology.

Paulian collected the species in 1956 in the western dry deciduous forest south of Befasy, Morondava. We rediscovered the species in 2009, when we found one female specimen of *C. befasicus* also in the western part but a bit further north than the type locality, along the road between Morafenobe and Beravina village at an altitude of 290 m. This locality consisted of dry savannah with mixed wood and grassland ecosystem after deforestation. The habitat consisted of muddy/sandy residual pools with some dead leaves in a temporary stream after the rainy season. The dry deciduous forest ecosystem in western Madagascar has suffered immensely from deforestation and very little of this habitat remains (Ganzhorn et al. 2001; Whitehurst et al. 2009). The species is rare and likely threatened due to the disappearance of western deciduous forests in Madagascar. That all four known specimens are females may suggest an uneven sex ratio as a test of equal sex ratio is marginally non-significant (p = 0.0625) if considered randomly picked from the population.

##### Comments.

This is the only species in the *Copelatus longicornis* group from Madagascar. The *longicornis* species group currently contains 38 species distributed mainly in the Neotropical and Afrotropical regions but also with few species present in Japan, New Guinea, and Fiji Islands (Nilsson and Hájek 2018). The group as currently defined is certainly artificial from a phylogenetic perspective and the only character they have in common is the low number of elytral striae.

### The *Copelatus irinus* group

The *irinus* group is characterised by the presence of six discal and one submarginal elytral striae (Sharp 1882; Guignot 1961; Guéorguiev 1968; Nilsson et al. 1997). There are six species reported from Madagascar from this group (Rocchi 1991): *Copelatus distinguendus* Régimbart, 1903, *Copelatus mahajanga* Pederzani & Hájek, 2005, *Copelatus aldabricus* J. Balfour-Browne, 1950, *Copelatus mimetes* Guignot, 1957, *Copelatus insuetus* Guignot, 1941, and *Copelatus nodieri* Régimbart, 1895. Here we describe five new species of *Copelatus* belonging to the *irinus* group based on the elytral striae: *Copelatus ankaratra* sp. nov., *Copelatus kely* sp. nov., *Copelatus pseudostriatus* sp. nov., *Copelatus safiotra* sp. nov., and *Copelatus vokoka* sp. nov. It is clear from the male genitalia, however, that *C. safiotra* sp. nov. is phylogenetically closer to the radiation of the *C. owas* complex in the *erichsonii* group with ten discal and one submarginal striae. We consider the record of *C. nodieri* from Madagascar as misidentified, and this is discussed below (see under *Copelatus* sp_Bemaraha: sp 1).

#### Copelatus distinguendus

Taxon classificationAnimaliaColeopteraDytiscidae

Régimbart, 1903

48693E4733015386A1417218CDEAFB7A

Figs 4B , 7D

Copelatus distinguendus Régimbart, 1903: 19 [nom. nov., referring to his description of Malagasy material under the name Copelatus duodecimstriatus Aubé in Régimbart 1895: 163]

##### Type locality.

Environs de Tananarive [surroundings of Antananarivo] and Fianarantsoa, Madagascar.

##### Type information from original description.

Based on an unknown number of specimens (syntypes) collected by Sikora (Antananarivo) and Perrot (Fianarantsoa).

##### Type material studied.

Type material in MNHN not studied, as it was out on loan.

##### Additional material studied.

**Fianarantsoa. Matsiatra Ambony: Ambalavao, Ambohimahasoa, Lalangina**: -2♀, 1♂(GP) (NHMUK): // BMNH-792954–6 // MAD: FIAN: Andringitra | Zomandao R.: River edge: Bottle trap | P39EM08: N: -22.1043: E:46.92: 1420 m | 09/V/2006: Leg. Bergsten et al // BMNH (DNA Voucher) // -1♀ (NMW): // Data in NHRS | JLKB | 000010718 // Madagascar: Ambohimahasoa (Fianarantsoa) | RN7 (Km 378) | 16.04.2011: Leg. R. Gerecke (MD211) // spring area with meadow swamps Exp. E: 21°15'41.5"S, 47°14'10.9"E, 1500 m // -2♀ (NMW): // Data in NHRS | JLKB | 000065754–5 // Madagascar Est, 1100– | 1200m, P.N. Ranomafana // Vohiparara, 21.–24.1.1993 | J. Janák lgt // -1♂(GP), 1♀, 5 ex. (Alc.) (NHRS): // NHRS-JLKB | 000010618–9, 10787(Alc.) // Madagascar: Fianarantsoa: Matsiatra | Ambony: Ranomafana NP: | Sahamalaotra 2Km from Vohiparara: | S21.23807, E047.39489, 1140 m.| 01:XI:2011: stamping with sieves: forest | bog in rainforest: Field# MAD11-12 // Leg. J. Bergsten, R. | Bukontaite, T. | Ranarilalatiana & | H.J. Randriamihaja // -1♀(Alc.) (NHRS): // NHRS-JLKB | 000010830 // MAD: FIAN: Matsiatra Ambony | Ranomafana NP: 450m along | Sahamalaotra trail, left at the first | junction: Mad14-07: forestmarsh: | 21.2382S 49.3947E: 1130 m: 02.XI.2014 // Leg. J. Bergsten, | T. Ranarilalatiana | & S. Holmgren // -1♀ (NHMUK): // BMNH-670601_MSL007 | 06.xii.2004, Ranomafana, | Madagascar: lat - 21.2359 | Lon 47.3963 Coll Balke_M; | Monaghan_M // DNA Voucher | BMNH <670601> | MSL007:E07 // **Fianarantsoa. Amoron’i Mania: Ambositra**: -3♀ (NHMUK): // BMNH-792962–4 // Col de Tapias: Rte Tana–Fianarantsoa: Pond | P36C: N: -20.772: E:47.179: 1717 m | 06/V/2006: Leg. Bergsten et al // BMNH (DNA Voucher) // -1♀ (NHMUK): // BMNH-792912 // Ambositra: Ankazomivady forest | 01.xii.2005 // BMNH (DNA Voucher) // -9♀ (NHMUK), 8 ex. (Alc.) (NHRS): // BMNH-729890, 729893, 729896–7, 792976–80, 10793(Alc.) // 08.xii.2004, Col de Tapias, | P30MD33: lat -20.238 | Lon 47.1 Coll Balke_M | Monaghan_M // BMNH (DNA Voucher) // **Antananarivo. Vakinankaratra: Ambatolampy**: -3♂(GP), 3♀, 10 ex. (Alc.) (NHRS, DEUA & PBZT/MBC): // NHRS-JLKB | 000010620–5, 10786(Alc.) // Madagascar: Antananarivo: | Vakinankaratra: Manjakatompo Stn. | forestière: 500m E Lac Froid by the | road: S19.34485 E047.33381, 1620 m. | 04.XI.2011: GB Nets and sieves: pond | and inlet stream: MAD11-16 // Leg. J. Bergsten, R. | Bukontaite, T. | Ranarilalatiana & | H.J. Randriamihaja // -1♂(GP) (NHRS): // NHRS-JLKB | 000010626 // Madagascar: Antananarivo: | Vakinankaratra: Manjakatompo Stn. | forestière: Analafandriana 500 m S | fish farm by the road: S19.36191 E | 47.31495, 1730 m, 03.XI.2011: GB | Nets: grassy pond: Field# MAD11-14 // Leg. J. Bergsten, R. | Bukontaite, T. | Ranarilalatiana & | H.J. Randriamihaja // -1♂(GP) (NHRS): // NHRS-JLKB | 000010660 // Madagascar: Ambatolampy: Manjaka- | tompo Ankaratra Reserve: MAD16-03: | “Lac froid”: S-19.34292; E047.33893; | 1651 m: lake with grass at margins: | 03/02/2016 Leg. T. Ranarilalatiana // -4♂(GP), 4♀, 5 ex. (Alc.) (NHRS, DEUA & PBZT/MBC): // NHRS-JLKB | 000010662–9, 10791(Alc.) // Madagascar: Ambatolampy: Manjaka- | tompo Ankaratra Reserve: MAD16-46: | “Lac froid”: S-19.34292; E047.33893; | 1651 m: lake with grass at margins: | 17/09/2016 Leg. T. Ranarilalatiana // -1♂(GP) (NHRS): // NHRS-JLKB | 000010661 // Madagascar: Ambatolampy: Manjaka- | tompo Ankaratra Reserve: MAD16-10: | Ankafotra mountain: S-19.33753; | E047.24530; 2466 m: streampools: | 07/02/2016; Leg. T. Ranarilalatiana // -4♂(GP), 2♀, 18 ex. (Alc.) (NHRS, DEUA & PBZT/MBC): // NHRS-JLKB | 000010670–5, 10792(Alc.) // Madagascar: Ambatolampy: Manjaka- | tompo Ankaratra Reserve: MAD16-47: | Ankafotra mountain: S-19.33753; | E047.24530; 2466 m: streampools: | 18/09/2016; Leg. T. Ranarilalatiana // -1♀ (Alc.) (NHRS): // NHRS-JLKB | 000010832 // Madagascar: Antananarivo: | Vakinankaratra: Manjakatompo Stn | forestière: Analamitana: S19.363972 E | 047.299083, 1757 m. 22:I:2012: swamp | near stream: Field# MJK12-02: Leg. T. | Ranarilalatiana & J.H. Randriamihaja // **Antananarivo. Analamanga: Anjozorobe, Ankazobe**: -1♂(GP), 1♀ (NHRS): // NHRS-JLKB | 000010684–5 // Madagascar: Anjozorobe: MAD16-36: | Amboasarianala: S-18.45792; E047. | 93438; 1367 m: Ambatovikinina stream: | 04/04/2016; Leg. T. Ranarilalatiana // -1♂(GP), 1♀ (NHRS): // NHRS-JLKB | 000010686–7 // Madagascar: Anjozorobe: MAD16-43:| Amboasarianala, Antanambe stream: | S-18.4671; E047.93807; 1271 m: Stream | with sidepools: 07/04/2016 | Leg. T. Ranarilalatiana // -2♂(GP), 2♀, 1♀(Alc.) (NHRS): // NHRS-JLKB | 000010688–91, 10790(Alc.) // Madagascar: Anjozorobe: MAD16-44 | Amboasarianala, Mangarivotra stream: | S-18.4676; E047.92535; 1271 m: stream | with bedrock and grass at edge | 07/04/2016; Leg. T. Ranarilalatiana // -2 ex. (Alc.) (NHRS): // NHRS-JLKB | 000010831 // MAD: ANTA: Analamanga: Anjoz | orobe forest reserve: Marsh next | to the stream by Saha forest, 10Km E of Anjozorobe: MAD14- | 78: forestmarsh: 18.4128S | 47.9439E; 1320 m; 23.XI.2014 // Leg. J. Bergsten, R. | Bukontaite, S. Holmgren, | J.H. Randriamihaja | & T. Ranarilalatiana // -2♂(GP), 2♀, 6 ex. (Alc.) (NHRS, DEUA & PBZT/MBC): // NHRS-JLKB | 000010676–9, 10797(Alc.) // Madagascar: Ankazobe: MAD16-24: | Firarazana: S-18.13132; E047.23976; | 1551 m; Lake with grass at margins: | 12/03/2016; Leg. T. Ranarilalatiana // -2♀, 11 ex. (Alc.) (NHRS, DEUA & PBZT/MBC): // NHRS-JLKB | 000010680–1, 10789(Alc.) // Madagascar: Ankazobe: MAD16-26: | Maharidaza, Large stream by the road to | military camp: S-18.22102; E047.27087; | 1547 m: stream and bog with grass: | 14/03/2016; Leg. T. Ranarilalatiana // -1♂(GP), 1♀, 7 ex. (Alc.) (NHRS): // NHRS-JLKB | 000010682–3, 10788(Alc.) // Madagascar: Ankazobe: MAD16-29: | Firarazana, SW of Ambohitantely | reserve: S-18.16717; E047.26090; | 1532 m: Bog with grass: 17/03/2016; | Leg. T. Ranarilalatiana // -3 ex. (Alc.) (NHRS): // NHRS-JLKB | 000010829 // MAD: ANTA: Analamanga: Mana- | nkazo river by the bridge of | RN4: Mad14-75: medium size river over bedrock: 18.158S | 47.2104E: 1450 m: 21.XI.2014 // Leg. J. Bergsten, | J.H. Randriamihaja | & T. Ranarilalatiana // -14 ex. (Alc.) (NHRS, DEUA & PBZT/MBC): // NHRS-JLKB | 000010834 // MAD: ANTA: Analamanga: Andra- | nofeno river by the bridge of | RN4, next to Andranofeno Sud | village: Mad14-74: medium size, | slow flowing river: 18.0844S | 47.1776E: 1430 m: 21.XI.2014 // Leg. J. Bergsten, | J.H. Randriamihaja | & T. Ranarilalatiana // - 1♀ (Alc.) (NHRS): // NHRS-JLKB | 000010835 // Madagascar: Ankazobe: TR18L10: | Stream by the bridge S of Ambohitantely | reserve: S-18.2023; E047.2780; 1556 m: | Hygropetric rock and stagnant pools: | 11/04/2018 | Leg. T. Ranarilalatiana -1♀ (NMW): // Data in NHRS | JLKB | 000065756 // RM: Betsiboka Bas (PO533) | Andranofeno Sud Riv. | 47°10'46"E, 18°05'00"S | 06.11.1995 | Leg. Elouard, J.-M., Oliarinony. R. // **Toamasina. Alaotra Mangoro: Ambatondrazaka, Andilamena, Moramanga**: -1♀ (NMW): // Data in NHRS | JLKB | 000065759 // E-Madagascar (09) Ambaton- | drazaka Region, 5Km N Didy | 1100–1200 m. asl. 14–16.01.1995 | G. Dunay & J. Janák coll. // -1♀ (NMW): // Data in NHRS | JLKB | 000065760 // E-Madagascar (10) | Ambatombe, near Andilamena | 900 m asl. 17.01.1995 | G. Dunay & J. Janák coll. // -1♂ (NMW): // Data in NHRS | JLKB | 000065761 // E-Madagascar (11) Ampamoho | near Andilamena, 1200–1300 m. | asl. 18–20.01.1995 | G. Dunay & J. Janák coll. // -1♂ (GP) (NHRS): // NHRS-JLKB | 000010627 // MAD: TOAM: Alaotra Mangoro | Betsabora river by RN2 near | Antsapanana village, 6 Km W | of Moramanga: MAD14-81: river | with side pools: 18.9247S | 48.1828E; 900 m; 24.XI.2014 // Leg. J. Bergsten, | J.H. Randriamihaja | & T. Ranarilalatiana // -1♂(Alc.) (NHRS): // NHRS-JLKB | 000010833 // Madagascar: Toamasina: Alaotra | Mangoro: Analamazaotra SR: bog | at S border of reserve: S18.95456 E | 048.44048, 910 m: 09.XI.2011: GB | Nets and sieves: bog with red mud: | Field# MAD11-27 // Leg. J. Bergsten, R. | Bukontaite, T. | Ranarilalatiana & | H.J. Randriamihaja // -1♀ (NHMUK): // BMNH-677103 // 11.xii.2004, Andasibe, | Madagascar: P27MD36 | Coll Balke_M; | Monaghan_M // (DNA Voucher) // -1♀ (NMW): // Data in NHRS | JLKB | 000065757 // MADAGASCAR (Md-4) | Andasibe, NP Perinet | 1150 m, Pfütze auf waldwiese | und in Kleinen Bach | 7.12.2000, Leg. W. Dolin // -1♀ (NMW): // Data in NHRS | JLKB | 000065758 // E-Madagascar (07) | Andranokobaka, N Moramanga | 800 m. Asl, 13.01.1995 | G. Dunay & J. Janák coll. // **Antsiranana. Sava: Andapa**: -1♂ (GP), 1♂ (Alc.) (NHRS): // NHRS-JLKB | 000010827, 10828 (Alc.) // MAD: ANTS: Sava: Anjanaharibe | Sud NP: river Marolakana at the | crossing place: Mad14-64: larger | river with rocks; 14.7623S | 49.4834E: 920 m: 15.XI.2014 // Leg. J. Bergsten, R. | Bukontaite, S. Holmgren, | J.H. Randriamihaja | & T. Ranarilalatiana //

##### Diagnosis.

Body shape elongate oval, convex, and attenuate posteriorly, with uniform black colouration (Fig. 7D). The pattern of abbreviation of the elytral striae separates this species from all other Malagasy *Copelatus* species. Striae 1, 3, and 5 present only in posterior one third (1) or two thirds (3, 5); striae 2, 4, and 6 fragmented anteriorly and, except for stria 4, never reach the base as clearly impressed striae. Penis profile in lateral view is characteristic, resembling a “pumpjack” (Fig. 4B).

##### Description.

Body length: 5.3–6.3 mm. Body shape elongate oval, convex, and attenuate posteriorly. Head, pronotum, and elytra all the same colour, ferrugineous black and finely punctate.

Lateral margin of pronotum rusty ferrugineous, with short sparse strioles. Pronotum with puncture rows and microsculpture. Elytra narrowly testaceous to ferrugineous posterolaterally. Six elytral striae present and one submarginal stria (Fig. 7D). Striae 2, 4, and 6 longer than striae 1, 3, and 5, at least as continued fragments; first stria shortest, present only in posterior fourth to posterior third. Second and third striae present as clearly impressed striae in posterior half or a little longer, but second stria continues, albeit fragmentary and less impressed, more distinctly all the way to base. Fourth stria the most complete, more or less present to base but less deeply impressed or fragmentary in anterior third. Fifth stria abbreviated a little less than third stria, present in approximately posterior two thirds, and like stria 3 has small isolated point remnants of stria more anteriorly. Sixth stria longer than fifth and with more distinct remnants of striae present anteriorly. Elytra covered with punctures in apical part.

Ventral side ferrugineous dark brown. Metacoxa and abdominal sternite II, III, IV, striolate. Prosternal process raised medially but rounded, not carinate. Lateral parts of metaventrite broad. Metacoxal lines short and strongly diverging anteriorly. Antenna, palps, pro- and mesothoracic legs brown to yellowish, but metathoracic legs dark brown.

Male: protibia slightly widened at apex, somewhat curved and angulate basally. Penis in lateral view curved with two points where curvature is more abrupt, constricted before apex at a narrow “neck” and expanding to apical part (Fig. 4B). As a whole in lateral view, the penis bears resemblance to the silhouette of a “pumpjack”. Penis apex in ventral view left-turned. Parameres broad at base, curved and tapering towards apex, apical part with numerous setae on ventral margin (Fig. 4B).

Female: dorsal sculpture similar to male.

##### Distribution.

Occurs on Madagascar and Mauritius (Gomy 2016). Guignot (1948) tentatively refer a single female specimen from Lulua in DR Congo to *C. distinguendus* but this was very likely a different species. In Madagascar, it has been recorded from the provinces of Antananarivo, Fianarantsoa Toamasina, and Antsiranana; more specifically from Anjozorobe Angavo reserve, around Ankazobe, Manankazo, Andranofeno Sud, around Ambohitantely reserve, Manjakatompo Ankaratra reserve; also from the eastern central area around Moramanga, Analamazaotra NP; in the southeast from Ambositra in col de Tapia, Ankazomivady forest, Ranomafana NP, Andringitra NP; and from the northeast in Anjanaharibe Sud reserve (Fig. 11D).

##### Habitat and ecology.

This species has been collected in various localities, mostly from open, partly deforested areas at mid- to high altitudes or open forest marshes. It occurs at altitudes above 900 m and is often associated with grass vegetation along lake shores and in marshes, found by stamping, and at vegetation-rich margins of rivers.

##### Comments.

Régimbart (1895) described this species under the name *Copelatus duodecimstriatus* Aubé but realised later (1903) that what he had described from Madagascar was not conspecific with Aubé’s *C. duodecimstriatus* from the Mascarene islands and he gave it the new name *C. distinguendus*, referring to his description from 1895. *Copelatus duodecimstriatus* lacks submarginal striae, has a distinctly different penis shape, and is endemic to Mauritius (Vinson 1967; Guignot 1961; Gomy 2016). The closest relatives of *Copelatus distinguendus* are likely a group of species on continental Africa with similar genitalia (e.g., *C. ateles* Guignot, 1955, *C. sylvaticus* Guignot, 1952, *C. andreinii* Régimbart, 1905, and additional species described by Bilardo and Rocchi 2013). These have a variable number of elytral striae from five discal to ten discal plus one submarginal, but similar “pumpjack”-shaped genitalia (Bilardo and Rocchi 2013). *Copelatus distinguendus* differs from these especially by the lack of a lateral tooth on the male penis in ventral view.

#### Copelatus pulchellus

Taxon classificationAnimaliaColeopteraDytiscidae

(Klug, 1834)

0149666EE3E15D2FA4E180FD888742E5

Figs 5C , 8D

Agabus pulchellus Klug 1834: t. XXXIII: 7
Copelatus africanus Sharp, 1882: 583; TL: Botswana, Lake Ngami;
? Copelatus basalis Boheman, 1848: 244; TL: South Africa (Caffraria interiore);
Copelatus discoideus Sharp, 1882: 582; TL: Mesopotamia;
Copelatus obtusus Boheman, 1848: 242; TL: South Africa (Caffraria orientali);
Copelatus strigulosus Sharp, 1882: 582; TL: Mesopotamia;
Copelatus mimetes Guignot, 1957: 73; TL: Madagascar, Sakaraha, Lambomakandro; syn. nov.

##### Type locality.

Egypt, Sinai.

##### Type material studied.

-HT♂ (GP) (*Copelatus mimetes*) (MNHN, “coll. Guignot”): // Data in NHRS | JLKB | 000030032 // Sakaraha | Lambomakandro | III-56 A.R. // Type [red label] // F. Guignot Det., 1956 | *Copelatus* | *mimetes* n. sp.| Type ♂ // INSTITUT SCIENTIFIQUE MADAGASCAR // -LT♀ (lectotype here designated) (*Copelatus obtusus*) (NHRS): // Caffra | ria. // J. Wahlb // Type. // HoloTypus [red label, “Typus” printed, “Holo” handwritten with small letters in front, possibly by J. Balfour-Browne who studied the type] // obtusus Boh. // *Cop. pulchellus* var: | *obtusus* Boh. | J. Balfour-Browne det. // 5657 | E91 // NHRS-JLKB | 000065335 // Lectotype | *Copelatus obtusus* | Boheman, 1842 | Des. Ranarilalatiana | & Bergsten, 2019// -? ST♂ (*Copelatus basalis*) (NHRS): // Caffra | ria. // J Wahlb // Type. // Typus [red label, printed] // 158 | 61 // Copelatus | basalis Bhn. // *Copelatus* | *pulchellus* | Klug. | Det. 19.iv.1961 | J. Omer-Cooper. // 5597 | E91 // NHRS-JLKB | 000065337 // -? ST♀ (*Copelatus basalis*) (NHRS): // Caffra | ria. // J. Wahlb // Paratypus [red label, printed] // 160 | 61 // 5596 | E91 // NHRS-JLKB | 000065338 // -? ST♀ (*Copelatus basalis*) (NHRS): // Caffra | ria. // J. Wahlb // Paratypus [red label, printed] // 159 | 61 // 5595 | E91 // NHRS-JLKB | 000065339 //

##### Additional material studied.

**Antsiranana. Diana: Antsiranana**: -1♂(GP) (MNHN): // Data in NHRS | JLKB | 000030259 // Madagascar | Diego-Suarez | Ch. Alluaud 1893 // **Mahajanga. Boeny: Mitsinjo**: -1♀ (NHRS): // NHRS-JLKB | 000065733 // Madagascar: Mahajanga: Boeny: | Mahavavy Kinkony RS: S 16.13337 | E 045.95778, 19 m.a.o. 04.XII.2009 | Water Net, Field# MAD09-25 | leg. J. Bergsten, N. Jönsson, | T. Ranarilalatiana, H.J. Randriamihaja // **Mahajanga. Melaky: Antsalova**: -1♂(GP) (NHRS): // NHRS-JLKB | 000010695 // Madagascar: Mahajanga: Melaky: | Tsingy de Bemaraha NP: S19.03572 | E044.77507, 66 m.a.o. 15.XII.2009 | Water Net, Field# MAD09-58 | Leg. J. Bergsten, N. Jönsson, T. | Ranarilalatiana, H.J. Randriamihaja //

##### Diagnosis.

**Figure 8.**
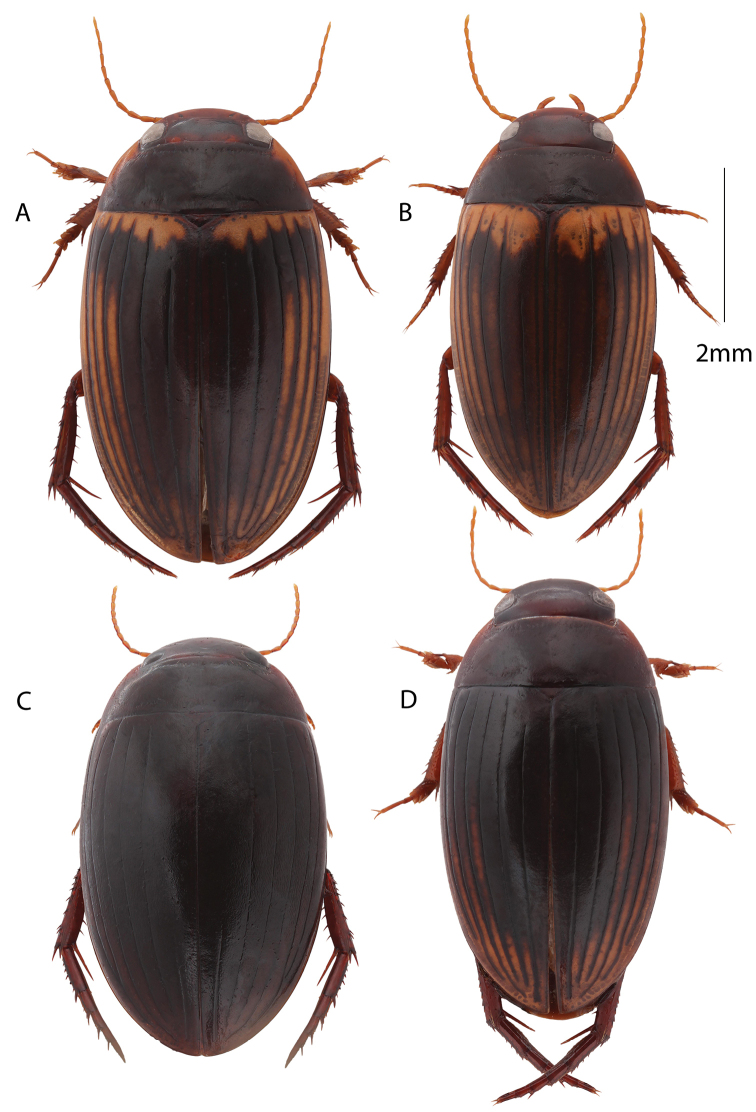
Habitus, dorsal view. **A** Male: *Copelatus marginipennis***B** Female: *Copelatus mahajanga***C** Female: *Copelatus* sp. 1 (Bemaraha) **D** Male: *Copelatus pulchellus*.

Similar to *C. marginipennis* (Laporte, 1835) and *C. mahajanga* in overall habitus, but body shape more like the elongated shape of *C. mahajanga*. *Copelatus pulchellus* on Madagascar is ferrugineous black in overall colouration with or without a rather narrow or vague testaceous band basally on elytra (Fig. 8D). Instead testaceous regions on elytra are limited to posterolateral and apical parts. The first elytral stria almost full length. Penis in lateral view rather abruptly curved past middle so that basal and apical parts are approximately at right angles (Fig. 5C). Penis overall simpler compared to that of *C. marginipennis* and *C. mahajanga* and apex slightly twisted.

##### Description.

(based on Malagasy specimens):

Body length 5.5–6.1 mm. Body shape oblong oval, rather convex and attenuate posteriorly, dark brown to blackish ferrugineous. Head infuscated brown ferrugineous, somewhat lighter posteriorly, covered with dense microreticulation and sparser punctation.

Pronotum dark brown to ferrugineous black with testaceous anterolateral corners. Disc covered with fine microsculpture forming regular cells and regularily spread small punctures of about same size as cells. Punctuation becomes coarser in posterolateral corners with a weak tendency to corrugate.

Elytra predominantely dark brown to ferrugineous black on disc and along striae with or without a rather narrow and vague basal testaceous band (Fig. 8D). Outer intervals and apical region testaceous to varying degrees, often a testaceous interval 4 stands out. Elytral surface covered with same type of microsculpture and punctures as on pronotum. Six clearly impressed elytral striae present on disc and one submarginal stria: second to sixth stria starting at base, first stria somewhat abbreviated anteriorly (by approximately 1/8^th^ the length of elytra); submarginal stria starting at approx. middle.

Ventral side ferrugineous dark brown, with testaceous spots laterally on abdominal ventrites. Metacoxa and ventrites with strioles. Prosternal process more elongate lanceolate and with blunter apex compared with *C. marginipennis* and *C. mahajanga*. Lateral parts of metaventrite medium broad. Metacoxal lines short and rather strongly diverging anteriorly. Antennae, palps, pro- and mesolegs testaceous, metalegs somewhat darker testaceous.

Female: elytral striolation limited to the medial parts of the outer three elytral intervals in the single female studied from Madagascar. From other parts of the distribution a female form is known that has the entire elytra striolated (Sharp 1882; Hájek and Reiter 2014).

Male: protibia bisinuate and angled at base, distally expanded. Penis thin, strongly angled at middle in lateral view, and apex somewhat twisted to the left in ventral view (Fig. 5C). Parameres more narrowly triangular apically compared to those in *C. marginipennis* and *C. mahajanga*.

##### Distribution.

As the species *C. pulchellus* is currently interpreted, this is a very widely distributed Afrotropical and Middle Eastern species. Balfour-Browne (1950) recorded the species from Senegal in the west, Tanzania in the east, South Africa in the south, and Mesopotamia (Iraq and adjacent regions) in the Middle East. Hájek and Reiter (2014) recorded the species from Yemen and stated that the likely distribution in the Middle East included the entire Arabian Peninsula. Guignot (1961) also gives India and Ceylon (= Sri Lanka) but this was likely based on the misidentification of closely related oriental species (see Ghosh and Nilsson 2012; Sheth et al. 2018). *Copelatus pulchellus* was not previously recorded from Madagascar following the revision by Balfour-Browne (1950) but as we synonymise *C. mimetes* with *C. pulchellus*, Madagascar now forms part of the distribution. From Madagascar we have seen specimens from Antsiranana, Mahavavy Kinkony Reserve, Tsingy de Bemaraha National Park (Bekopaka), and from Lambomakandro, Sakaraha (Fig. 11C). It can likely show up anywhere in lowland Madagascar, but especially in the western lowlands.

##### Habitat and ecology.

On Madagascar we have collected the species associated with a small forest stream with sidepools in a karstic limestone area (“tsingy”) and in a muddy stagnant pool in a dried-out river bed. Both localities are in dry deciduous forests of lowland western Madagascar.

##### Comment.

*Copelatus pulchellus* forms part of a diverse species group with many externally very similar species. Balfour-Browne (1950) admitted that his previous treatment of the species (Balfour-Browne 1939) was entirely wrong as he had then not studied the male genitalia. When he did so in 1950 it resulted in the description of several new species previously lumped under *C. pulchellus*.

*Copelatus pulchellus* is now interpreted as a widespread Afrotropical and Middle Eastern species with the male penis similar to that illustrated in Figure 5C. Dorsal colouration is interpreted as very variable, even consisting of several distinct colour forms such as the darker forms described as *C. obtusus* Boheman and later as *C. africanus* Sharp (compare figure 24 with photographs in Perissinotto et al. (2016: fig. 24) and Hájek and Reiter (2014: fig. 30)). Females are also interpreted as variable in the striolation pattern on the elytra. Sharp described a female form from Mesopotamia that was entirely striolated over the elytra under the name *C. strigulosus*, which is interpreted today as intraspecific variation of *C. pulchellus* (see photograph in Hájek and Reiter (2014: fig. 30)). We have seen three males and one female from Madagascar. All males are of the darker colour form, lacking a basal transverse testaceous band on elytra, similar to Boheman’s *C. obtusus*. The female has a weak basal testaceous band but is otherwise also most similar to the dark colour form. Striolation is limited to the middle of the outer three intervals. We have no doubt that the three non-type specimens examined are conspecific with *C. mimetes* Guignot. But after our comparison of the male genitalia of the type with *C. pulchellus* specimens from mainland Africa, we concluded that *C. mimetes* cannot be upheld as a separate species given how *C. pulchellus* is interpreted. We noted very minor differences in the penis apex which are not greater than differences between east, south, and west African specimens of *C. pulchellus*. The penis figured by Guignot (1957: fig. 2, reproduced by Nilsson et al. 1997: fig. 8) is very inaccurate, depicting a shape quite different from *C. pulchellus* but that does not correspond with the holotype. *Copelatus pulchellus* may still be a species complex for future studies to solve but currently it is a widespread and variable species distributed over Africa, Madagascar, and the Middle East.

*Copelatus basalis* Boheman, 1848 was synonymised with *C. pulchellus* by Omer-Cooper (1965). We have studied the same type material as Omer-Cooper and agree with this conclusion. However, we are not convinced that the material housed at NHRS as these types are the correctly identified types of the name *C. basalis*. Boheman (1848) describes in Latin four species from Johan August Wahlbergs collectings in Caffraria (South Africa) of which three are new, numbered 259 – *Copelatus pulchellus* Klug; 260 – *Copelatus obtusus*; 261 – *Copelatus striatellus*; and 262 – *Copelatus basalis*. For *C. pulchellus* the disc of elytra is described as having six striae. For the longer description of *C. obtusus* this is further elaborated to detail that there are six discal stria but entire elytra has seven striae “septem-striata” (six discal and one submarginal). This pattern and number of striae is consistent with the *Copelatus pulchellus* species group. The following species is correctly identified as *C. striatellus* with nine discal striae, and is clearly stated as such: “disco striis 9 tenuibus”, of which the innermost is much abbreviated. This description matches very well with the types preserved at NHRS. Finally, *C. basalis* is described as the last *Copelatus* species in Boheman’s work and elytra is described as “12-striata”. The supposed types at NHRS for both *C. obtusus* and *C. basalis* have six discal and one submarginal striae. The type for *C. obtusus* matches the original description of “septem-striata” but the three types of *C. basalis* with the same number of striae does not match the original description of “12-striata”. It would be very inconsistent of Boheman to describe the total number of striae on one elytron in one case “elytra…septem-striata”, and in the other only the discal striae but summing up the number from both elytral halves “elytra…12-striata”. It would also be very illogical to place *C. basalis* after the 9-striated *C. striatellus* if it has the same number of striae as *C. pulchellus* and *C. obtusus*, which come first. Similarily, Guignot (1961) was confused about Boheman’s *C. basalis* and listed the name both under the 12-striated *C. mocquerysi* Régimbart with a question mark, and under *C. pulchellus*. We consider the status of *C. basalis* Boheman as uncertain but we have not found any alternative potential type material at NHRS. We designate the single undoubted syntype of *C. obtusus* Boheman, 1848 in the NHRS collection as lectotype to preserve the stability of the name.

#### Copelatus marginipennis

Taxon classificationAnimaliaColeopteraDytiscidae

(Laporte, 1835)

C9FE8EA486D557DFA5B1A38A9F232743

Figs 5A , 8A

Colymbetes marginipennis Laporte, 1835: 102.
Copelatus aldabricus J. Balfour-Browne, 1950: 368 syn. nov.; TL: Seychelles, Aldabra Islands.
Copelatus aldabricus var. simplex Guignot 1952: 28 syn. nov.; TL: Madagascar.

##### Type locality.

Senegal [possibly mislabelled].

##### Type information from original description.

housed in Buquet collection and originating from Senegal; of *aldabricus*: based on male (holotype), J.C. Fryer collection, collected 1908-9 from Aldabra; of *simplex*: based on male and female syntypes from Madagascar without further locality data.

##### Type material studied.

-LT♂ (lectotype here designated) (*Colymbetes marginipennis*) (MNHN): // Data in NHRS | JLKB | 000065416 // *Copelatus marginipennis* Buquet | pulchellus var. Aubé | h. in Senegal D. Buquet // D. Sharp | Monogr. // Ex-Musaeo Déjean // *pulchellus* // Lectotype | *Colymbetes marginipennis* | Laporte, 1835 | Des. Ranarilalatiana | & Bergsten, 2019// -HT♂ (*Copelatus aldabricus*) (NHMUK): // Aldabra, 08-9. J.C.F. Fryer // Perey Sladen Trust | expedition. | 1913-170. // Type [red round label] // *Copelatus* | *aldabricus* Type | J. Balfour-Browne det. //

##### Additional material studied.

**Toamasina. Alaotra Mangoro: Andilamena, Moramanga**: -1♀ (NMW): // Data in NHRS | JLKB | 000010726 // Madagascar 17.01.1995 | Ambatombe | nr. Andilamena 900 m | leg. Dunay & Janák (10) // -1♀ (NHMUK): // BMNH-797894 // MAD: AMPA: Moramanga: Andasibe | Andasibe NP: Big Pond | P61BI15: N: -18.937: E:48.416: 940 m | 06/I/2007: Leg. Isambert et al. // DNA Voucher | BMNH <797894> | MSL294:B10 // -2♂ (GP), 15 ex. (Alc.) (NHRS, DEUA & PBZT/MBC): // NHRS-JLKB | 000010587–8, 10799(Alc.) // Madagascar: Tamatave: Alaotra Mangoro: | Analamazaotra RS; Bas fond, non-permanent | pond near trail to “lac rouge”MAD15-1| 943 m, 18°56'26.7"S, 048°25'03.9"E, 16.III.2015 | Among vegetation and dead leaves in the pond, | Leg. T. Ranarilalatiana & H.J. Randriamihaja // -2♂ (GP), 2♀ (Alc.) (NHRS): // NHRS-JLKB | 000010604–5, 10800(Alc.) // Madagascar: Tamatave: Alaotra Mangoro: Mantadia NP. | Non-permanent pond at PK18,50 m; E of Park road | 973 m, 18°46'09.9"S, 048°26'10.4"E, 17.III.2015 | Under dead leaves & vegetation at the edge of the pond, | Leg.T. Ranarilalatiana, H.J. Randriamihaja; MAD15-5 // -1♂ (GP) (NHRS): // NHRS-JLKB | 000010815 // Madagascar: Toamasina: Alaotra | Mangoro: RN2, Mangoro river | 10Km W of Moramanga: S18.92438 | E048.18273, 940 m. 06.XI.2011 | GB Nets and sieves: river and | pools: Field# MAD11-21 // Leg. J. Bergsten, R. | Bukontaite, T. | Ranarilalatiana & | J.H. Randriamihaja // -1♀ (Alc.) (NHRS): // NHRS-JLKB | 000010822 // Madagascar: Toamasina: Alaotra | Mangoro: Analamazaotra SR: | close to park entrance: S18.9355 E | 048.41656, 970 m: 08.XI.2011: GB | Nets and sieves: dried up forest | pond: Field# MAD11-25 // Leg. J. Bergsten, R. | Bukontaite, T. | Ranarilalatiana & | H.J. Randriamihaja // -6 ex. (Alc.) (NHRS): // NHRS-JLKB | 000010823 // Madagascar: Toamasina: Alaotra | Mangoro: by RN2 S border of | Analamazaotra reserve 1Km E | Antsampanana: S18.94987 E048.42331| 980m: 09.XI.2011: GB Nets & sieves | ditch next to road: Field# MAD11-29 // Leg. J. Bergsten, R. | Bukontaite, T. | Ranarilalatiana & | J.H. Randriamihaja // **Toamasina. Analanjirofo: Maroantsetra**: -3♂ (GP), 2♀ (NHMUK): // BMNH-797906–10 // MAD: TOAM: Maroantsetra: Masoala | Masoala NP: Pool | P58BI14: N: -15.758: E: 49.993: 10 m| 17/XI/2006: Leg. Isambert et al. // BMNH (DNA Voucher) // -1♂ (GP) (NHRS): // NHRS-JLKB | 000010794 // MAD: TOAM: Maroantsetra: Masoala | Masoala NP: Pool | P58BI14: N: -15.758: E: 49.993: 10 m| 17/XI/2006: Leg. Isambert et al. // -1♂ (NMW): // Data in NHRS | JLKB | 000010728 // E-Madagascar: Fenerive | Foret de Tampolo | 28.12.1998 | leg. J. Moravec // -1♀(Alc.) (NHRS): // NHRS-JLKB | 000011122 // Madagascar: Toamasina: Analajinrofo: | Masoala NP: degraded lowalt. forest: | MAD18-47: small waterpools on path | ~0.5km NW of Marofototra village, | 15.7606S, 49.9926E, 15 m, 17.II.2018 | Leg. T. Ranarilalatiana // **Toliara. Menabe: Morondava**: -3♂ (GP) 2♀ (Alc.) (NHRS): // NHRS-JLKB | 000010580, 10730 (JB197), 10751, 10802(Alc.) //Madagascar: Toliara: Menabe: | Kirindy RS: S20.07430 | E044.66307, 52 m.a.o. 12.XII.2009 | Water Net, Field# MAD09-46 | Leg: J. Bergsten, N. Jönsson, T. | Ranarilalatiana, H.J. Randriamihaja // **Mahajanga. Boeny: Ambato-Boeny, Mitsinjo**: -1♂ (NHRS): // NHRS-JLKB | 000010734 (JB201) // Madagascar: Mahajanga: Boeny: | Ankarafantsika NP. S16.30350 | E046.81068, 87 m.a.o. 29.XI.2009 | Water Net, Field# MAD09-03 | Leg. J. Bergsten, N. Jönsson, | T. Ranarilalatiana, H.J. Randriamihaja // -6♂ (GP), 3♀, 11 ex. (Alc.) (NHRS, DEUA & PBZT/MBC): // NHRS-JLKB | 000010567–71, 10729 (JB196), 10732 (JB199), 10742–3, 10798(Alc.) // Madagascar: Mahajanga: Boeny: | Ankarafantsika NP. S16.30341 | E046.81073, 74 m.a.o. 29.XI.2009 | 22W Black Light, Field# MAD09-07 | Leg. J. Bergsten, N. Jönsson, | T. Ranarilalatiana, H.J. Randriamihaja // -1♂ (NHRS): // NHRS-JLKB | 000010731 (JB198) // Madagascar: Mahajanga: Boeny | Ankarafantsika NP, S16.30270 | E046.80996; 75 m.a.o. 30.XI.2009 | 22W Black light, Field# MAD09-13 | Leg. J. Bergsten, N. Jönsson, | T. Ranarilalatiana, H.J. Randriamihaja // -1♀ (NHRS): // NHRS-JLKB | 000010737 (JB191) // Madagascar: Mahajanga: Boeny: | Ankarafantsika NP. S16.31418 | E046.81731, 30.XI.2009 | Hand picking, Field# MAD09-14 | Leg. J. Bergsten, N. Jönsson, | T. Ranarilalatiana, H.J. Randriamihaja // -3♀, 1♂(GP), 11 ex. (Alc.) (NHRS, DEUA & PBZT/MBC): // NHRS-JLKB | 000065749 (JB809), 10738 (JB192), 10744–5, 10806(Alc.) // Madagascar: Mahajanga: Boeny: | Mahavavy Kinkony RS. S16.14653 | E045.94926, 9 m.a.o. 04.XII.2009 | Water net, Field# MAD09-24 | Leg. J. Bergsten, N. Jönsson, T. | Ranarilalatiana, H.J. Randriamihaja // -2♂ (GP), 2♀, 18 ex. (Alc.) (NHRS, DEUA & PBZT/MBC): // NHRS-JLKB | 000010578–9, 10739 (JB193), 10746, 10807(Alc.) // Madagascar: Mahajanga: Boeny: | Mahavavy Kinkony RS. S16.13337 | E045.95778, 19 m.a.o. 04.XII.2009 | Water net, Field# MAD09-25 | Leg. J. Bergsten, N. Jönsson | T. Ranarilalatiana, H.J. Randriamihaja // -5♂ (GP), 4♀, 79 ex. (Alc.) (NHRS, DEUA & PBZT/MBC): // NHRS-JLKB | 000010572–7, 10740 (JB194), 10747–8, 10808 (Alc.) // Madagascar: Mahajanga: Boeny: | Mahavavy Kinkony RS. S16.05776 | E045.80585, 22 m.a.o. 05.XII.2009 | Water net, Field# MAD09-28 | Leg. J. Bergsten, N. Jönsson, | T. Ranarilalatiana, H.J. Randriamihaja // -1♂, 1♀, 4 ex. (Alc.) (NHRS): // NHRS-JLKB | 000010735 (JB202), 10749, 10809(Alc.) // Madagascar: Mahajanga: Boeny: | Mahavavy Kinkony RS. S16.06651 | E045.77672, 24 m.a.o. 05.XII.2009 | Water net, Field# MAD09-29 | Leg. J. Bergsten, N. Jönsson, T. | Ranarilalatiana, H.J. Randriamihaja // -1♀ (NHRS): // NHRS-JLKB | 000010741(JB189) // Madagascar: Mahajanga: Boeny | Mahavavy Kinkony RS, 16.05648S | 045.76371E; 55 m.a.o. 05.XII.2009 | Water net, Field# MAD09-30 | Leg: J. Bergsten, N. Jonsson, T. | Ranarilalatiana, H.J. Randriamihaja // -1♂, 6ex. (Alc.) (NHRS): // NHRS-JLKB | 000010750, 10810(Alc.) // Madagascar: Mahajanga: Boeny: | Mahavavy Kinkony RS. S16.01334 | E046.00376, 24 m.a.o. 06.XII.2009 | Water net, Field# MAD09-33 | Leg. J. Bergsten, N. Jönsson, | T. Ranarilalatiana, H.J. Randriamihaja // **Mahajanga. Melaky: Antsalova**: -2♂ (GP) (CAS): // CASENT-8135015–6 // Madagascar: Mahajanga | Prov. Parc National Tsingy | de Bemaraha, 2.5 Km 62° ENE | Bekopaka, Ankidrodroa river | elev 100 m: 11–15 Nov 2001 // 19°7'56"S, 44°48'53"E | Coll: Fisher, Griswold et al. | California Acad. of Sciences | sifted litter - tropical dry forest | on Tsingy, code: BLF4340 // -1♂ (GP) (CAS): // CASENT-8131891 // Madagascar: Mahajanga | Prov. Parc National Tsingy | de Bemaraha, 2.5 Km 62° ENE | Bekopaka, Ankidrodroa river | elev 100 m: 11–15 Nov 2001 // 19°7'56"S, 44°48'53"E | Coll: Fisher, Griswold et al. | California Acad. of Sciences | at light- tropical dry forest | on Tsingy, code: BLF4343 // -1♂ (GP) (CAS): // CASENT-8135006 // Madagascar Mahajanga | Prov. Foret de Tsimembo | 11.0Km 346° NNW Soatana | elev 50 m: 21–25 Nov 2001 | 18°59'43"S, 44°26'37"E // Coll: Fisher, Griswold et al. | California Acad. of Sciences | sifted litter (leaf mold, rotten wood) | in tropical dry forest | coll. Code: BLF4508 // -1♂ (GP), 2♀, 3 ex. (Alc.), 2♂(Alc.) (NHRS): // NHRS-JLKB | 000010581–2, 10752, 10803(Alc.), 10821(Alc.)// Madagascar: Mahajanga: Melaky: | Tsingy de Bemaraha NP: S19.03572 | E044.77507, 66 m.a.o. 15.XII.2009 | Water Net, Field# MAD09-58 | Leg: J. Bergsten, N. Jönsson, T. | Ranarilalatiana, H.J. Randriamihaja // -3♂ GP, 1♀, 5 ex. (Alc.) (NHRS): // NHRS-JLKB | 000010583–4, 10736 (JB203), 10754, 10805(Alc.) // Madagascar: Mahajanga: Melaky: | Tsingy de Bemaraha NP: S18.75643 | E044.71398, 119 m.a.o. 17.XII.2009 | Water Net, Field# MAD09-65 | Leg: J. Bergsten, N. Jönsson, T. | Ranarilalatiana, H.J. Randriamihaja // -1♀, 1♂ (GP), 2♀ (Alc.) (NHRS): // NHRS-JLKB | 000010733 (JB200), 10753, 10804(Alc.) // Madagascar: Mahajanga: Melaky: | Tsingy de Bemaraha NP: 19.03419S | 044.77499E, 41 m.a.o. 15.XII.2009 | Water Net, Field# MAD09-59 | Leg: J. Bergsten, N. Jönsson, T. | Ranarilalatiana, H.J. Randriamihaja // **Antsiranana. Diana: Ambanja, Ambilobe, Antsiranana II**: -1♂ (GP) (NHRS): // NHRS-JLKB | 000010585 // Madagascar: Antsiranana: Diana: | Ambilomagodra: Stream under the | bridge of the road RN6 in | Ambilomagodra village, S13.00780 | E49.13313, 139 m, 30.XI.2012, GB nets: | dried up stream with some pools in | the village: Field# MAD12-33 // Leg. J. Bergsten, R. | Bukontaite, T | Ranarilalatiana & | J.H. Randriamihaja // -1♂ (GP), 2♀ (Alc.) (NHRS): // NHRS-JLKB | 000010586, 10801(Alc.) // Madagascar: Antsiranana: Diana: | Andrafiabe: Antsoha stream | 200m from Andrafiabe, 12.93022S | 49.03466E, 32 m, 01.XII.2012, GB | nets: stream with some pools: | Field# MAD12-39 // Leg. J. Bergsten, R. | Bukontaite, T | Ranarilalatiana & | J.H. Randriamihaja // -1♂ (Alc.) (NHRS): // NHRS-JLKB | 000011123 // Madagascar: Antsiranana: Diana: | Antsaba: 1km W of Antsaba, | 13.63474S; 48.72918E, 67 m, | 28.XI.2012, GB nets: forest | stream: Field# MAD12-32 // Leg. J. Bergsten, R. | Bukontaite, T | Ranarilalatiana & | J.H. Randriamihaja // -1♀ (NMW): // Data in NHRS | JLKB | 000010725 // MADAGASCAR: Sakaramy | (M. d’Ambre, Antsiranana) | lake Farihy Makery | 29.03.2011 | leg. R. Gerecke (MD189) // riparian area near outflow | 12°26'20.5"S, 49°14'20.0"E | 377 m. 29.5 °C // -1♂ (NMW): // Data in NHRS | JLKB | 000010727 // N. Madagascar | Antseranana distr. | Sambirano riv. | Marovato vill. | 5–12.12.01, leg. J. Horák // Antsira**nana. Sava: Sambava, Vohemar**: -1♀ (NHMUK): // BMNH-797876 // MAD: DIEG: Sambava: Marojejy | Marojejy NP: Pool | P57BI31: N: -14.457: E:49.79: 162m | 10/XI/2006; Leg. Isambert et al // DNA Voucher | BMNH <797876> | MSL294:A4 // -1♀ (CAS): // CASENT-8135007 // Madagascar Antsiranana | Foret d’Ampondrabe. | 26.3Km 10° NNE Daraina | elev 175m: 10 December 2003 // 12°58'12"S, 049°42'00"E | California Acad. of Sciences | Coll: B.L.Fisher, sifted | litter (leaf mold, rotten wood) | tropical dry forest, BLF9974 //

##### Diagnosis.

*Copelatus marginipennis* is distinguished from all other Malagasy *Copelatus* except *C. pulchellus* and *C. mahajanga* by the presence of six discal elytral striae and a broadly oval body shape. *Copelatus marginipennis* is most easily separated by the distinct shape of the male penis in lateral view (Fig. 5A). *Copelatus pulchellus* (from Madagascar) lacks a transverse testaceous band basally on the elytra and females of *C. marginipennis* can usually be distinguished from *C. mahajanga* by the normally narrower testaceous band, a longer first elytral stria, and a broader body shape.

##### Description.

Body length 5.2–6.6 mm. Body shape oval, rather convex and attenuate posteriorly, dark brown to brown ferrugineous. Head, pronotum and elytra in the same dark brown ferrugineous, covered with fine dense punctation. Lateral sides of pronotum more brownish, with short strioles and the widest striolate area in the posterior corners. Elytra dark brown to brown ferrugineous, with a testaceous transverse band at base (Fig. 8A). Six clearly impressed elytral striae present and one submarginal: first stria abbreviated and slightly shorter, starting at approx. 1/7^th^ posterior of elytral base, second to sixth full length, and submarginal stria starting at approx. the middle; all striae approaching the apex of elytron except the sixth, which is abbreviated posteriorly and a little shorter. Sometimes, the first and second elytral striae, like fifth and sixth, unite posteriorly. Elytral surface covered with dense punctures.

Ventral side brownish to ferrugineous, metacoxa with microsculpture, densely and finely punctate. Metacoxa and abdominal ventrites striolate. Prosternal process rather short and spear-shaped, medially only weakly raised and rounded. Lateral parts of metaventrites rather broad. Metacoxal lines short and rather strongly diverging anteriorly. Antennae and palps both in the same brown colour. Pro- and mesothoracic legs brown ferrugineous. Metathoracic legs dark brown ferrugineous.

Male: Protibia strongly angled basally and expanded in apical two thirds. Penis in ventral view with a small preapical tooth on right side; in lateral view very characteristic with a subbasal dorsal knob, and post-middle with a deep ventral invagination (Fig. 5A).

Females from Madagascar usually with elytral striolation rather weak and restricted to outer intervals, but rarely the elytra are entirely and distincly striolate. Females on average smaller than males.

##### Distribution.

Endemic to the western Indian Ocean islands as far as is modernly known, but the 1835 type locality of the original description and labels read “Senegal”, which indicates either mislabeling or that the species in fact also occurs on continental Africa. Known from Madagascar, Reunion, Comores, Seychelles, and Aldabra Island (Gomy 2016). Widespread in Madagascar and recorded from Montagne d’Ambre NP and Masoala NP in the north and northeast to Kirindy in the southwest (Fig. 11A), but apparently lacking from most of the central plateau. It can probably show up anywhere around the island below 1000 m in altitude.

##### Habitat and ecology.

This is a lowland species that seems to be most common in the deciduous western parts of Madagascar. It was common in the newly designated protected areas of Mahavavy Kinkony when we visited it in 2009. In the deciduous forest biome it has been recorded from Kirindy in the south to Ankarana NP in the north. The species seems to be a generalist and can as well show up on lowland humid east coast and at midaltitudes up to at least 1000 m. It is an apt flier collected with light traps and often in very temporary and small shallow pools including water-filled wheel tracks. Found in all kinds of temporary pools, as well as in streams and in residual pools in dried-up riverbeds. It occurred sympatrically with *C. mahajanga* in Betsabora river near Moramanga and at a 22W black light trap by a forest pool in Ankarafantsika NP.

##### Comments.

*Copelatus aldabricus* was described by J. Balfour-Browne (1950) from Aldabra Island in the Seychelles. Guignot (1952) named a non-striolated female variety from Madagascar as var. simplex, and again in 1961 as var. aequabilis (non-available infrasubspecific name) from Andranofotsy (E of Maroantsetra NE Madagascar), and stated (1961) that females of *C. aldabricus* are normally striolated, but that on Madagascar the non-striolated var. aequabilis is most common or even ubiquitous. We have found at least one female on Madagascar (from Masoala NP) with the elytra entirely striolated (the “normal” variety sensu Guignot 1961), but indeed the form where striolation is rather weak and only at outer elytral intervals is most common. Guignot’s (1961) distinction between female forms either having or lacking strioles is, however, a simplification. All females we have seen have at least some weak tendencies of striolation on outer intervals and the extent can vary significantly between individuals.

*Copelatus marginipennis* (Laporte, 1835) has been treated as a junior synonym of *Copelatus pulchellus* Klug, 1834 since Aubé (1838). We discovered the type material of Laporte´s *C. marginipennis* in MNHN, Paris. This could be identified based on a folded blue label bearing the information “*Copelatus marginipennis* Buquet, *pulchellus* var Aubé, h. in Senegal, D. Buquet”, which fits perfectly the description by Laporte (1835). The genitalia of this male show without any doubt that it is a synonym of the younger name *C. aldabricus* and not of *C. pulchellus*. The name *marginipennis* has to our knowledge not been used as a valid name after 1899, fulfilling the first of the two criteria for protecting a younger currently used synonym from an older available name (ICZN 23.9.1.1). However, *Copelatus aldabricus* is a relatively recent name from a region with a low-intensity taxonomic research and we do not believe it fulfils the second criteria of at least 25 papers published by at least ten authors in the immediately preceding 50 years and encompassing a span of not less than ten years (ICZN 23.9.1.2). Therefore it is not justifiable to protect the younger synonym *C. aldabricus* and the older *C. marginipennis* is therefore brought back as the valid name of this species. We designate the discovered syntype of *Colymbetes marginipennis* Laporte, 1835 in the MNHN collection as lectotype to preserve stability of the name. At the same time as this type specimen was discovered, also a likely “type” specimen of the nomen nudum *Copelatus fimbriolatus* Dejean, 1837 from “Ile de France” [= Mauritius] was found with the same type of blue folded label (see supplementary file 1: NHRS-JLKB000065420). This nomen nudum is currently considered a synonym of *C. pulchellus* Klug (Nilsson and Hajek 2018). Unfortunately, it is a female specimen, which is why its identity cannot be established with certainty, but it is more likely identical with *C. marginipennis* since *C. pulchellus* is not yet known from any of the Mascarene Islands (Gomy 2016).

#### Copelatus mahajanga

Taxon classificationAnimaliaColeopteraDytiscidae

Pederzani & Hájek, 2005

BD7D9CA1871E598AAC575F54EB7455FD

Figs 5B , 8B

Copelatus mahajanga Pederzani & Hájek, 2005: 104.

##### Type locality.

Madagascar, Mahajanga Distr., Mahajanga env. [ca. 15°43'S, 46°18'E]

##### Type information from OD.

Based on male (holotype), 3 male and 4 female (paratypes). I. Janis 1–10 December 1996. HT & 5PT in NMPC. Two paratypes in collection of Pederzani, Ravenna, Italy.

##### Type material studied.

**Mahajanga. Boeny: Mahajanga I/Mahajanga II** [district cannot be verified based on original description or labels]: -HT♂(GP) (NMPC): // MADAGASCAR 1996, Mahajanga Distr., Mahajanga env., Dec. 1–10., I. Janiš leg. // HOLOTYPE, COPELATUS, *mahajanga* sp. nov., F. Pederzani & J. Hájek det.2005 //

##### Additional material studied.

**Mahajanga. Boeny: Ambato-Boeny, Mitsinjo, Soalala**: -3♂ (GP), 1♀ (NMW): // Data in NHRS | JLKB | 000010719–22 // RM: Betsiboka Bas (PO124) | Loc. Ambohimanatrika | Kamoro Riv. // 47°10'06", 16°28'55" | alt. 40 m 01.04.1993 // Leg. ORSTOM ANTANANARIVO // -1♂ (GP) (NHRS): // NHRS-JLKB | 000010717 // Madagascar: Mahajanga: Boeny: | Ankarafantsika NP. S16.30341 | E046.81073, 74 m.a.o. 29.XI.2009 | 22W Black Light, Field# MAD09-07 | Leg. J. Bergsten, N. Jönsson, | T. Ranarilalatiana, H.J. Randriamihaja // -2♀ (NHRS): // NHRS-JLKB | 000065747 (JB802), 65732 // Madagascar: Mahajanga: Boeny: | Mahavavy Kinkony RS. S16.13337 | E045.95778, 19 m.a.o. 04.XII.2009 | Water net, Field# MAD09-25 | Leg. J. Bergsten, N. Jönsson, | T. Ranarilalatiana, H.J. Randriamihaja // -2♀ (NHRS): // NHRS-JLKB | 000010724 (JB195), 65748 // Madagascar: Mahajanga: Boeny: | Mahavavy Kinkony RS. S16.01334 | E046.00376, 24 m.a.o. 06.XII.2009 | Water net, Field# MAD09-33 | Leg. J. Bergsten, N. Jönsson, | T. Ranarilalatiana, H.J. Randriamihaja // -2♂ (GP), 3♀ (CAS): // CASENT-8131811–5 // Madagascar: Mahajanga | Province, Parc National de | Namoroka 16.9 km 317° NW | Vilanandro 12–16 Nov 2002 // 16°24'24"S, 045°18'36"E // Colls. Fisher, Griswold et al.| California Acad. of Sciences | sifted litter, (leaf mold, rotten | wood) in tropical dry forest | elev 100 m code: BLF6582 // -1♀ (CAS): // CASENT-8135013 // Madagascar: Mahajanga | Prov. Parc National de | Namoroka 16.9 Km 317° NW | Vilanandro elev 100 m | 12–16 November 2002 // 16°24'24"S, 045°18'36"E | Coll. Fisher, Griswold et al. | California Acad. of Sciences | malaise trap- | tropical dry forest | collection code BLF6581 // -1♂ (GP) (CAS): // CASENT-8135001 // Madagascar: Mahajanga | Province Parc National de | Namoroka | 17.8 Km 329° WNW Vilanandro | elev 100 m: 08–12 Nov 2002 // 16°22'36"S, 045°19'36"E | Colls. Fisher, Griswold et al. | California Acad. of Sciences | pitfall trap- in tropical dry forest, | collection code BLF6506 // **Mahajanga. Melaky: Antsalova**: -1♂ (GP) (NHRS): // NHRS-JLKB | 000010723 (JB190) // Madagascar: Mahajanga: Melaky: | Tsingy de Bemaraha NP: S19.03572 | E044.77507, 66 m.a.o. 15.XII.2009 | Water Net, Field# MAD09-58 | Leg. J. Bergsten, N. Jönsson, T. | Ranarilalatiana, H.J. Randriamihaja // **Toamasina. Alaotra Mangoro: Moramanga**: -5♂ (GP), 3♀, 11 ex. (Alc) (NHRS, DEUA & PBZT/MBC): // NHRS-JLKB | 000010554–5, 10596–601, 10795 (Alc.) // MAD: TOAM: Alaotra Mangoro: | Betsabora river by RN2 near | Antasmpanana village, 6Km W | of Moramanga: MAD14-81: river | with side pools: 18.9247S | 48.1828E: 900 m: 24.XI.2014 // Leg. J. Bergsten, | J.H. Randriamihaja | & T. Ranarilalatiana // -3♂ (GP) 26 ex. (Alc.), 7 ex. (Alc.) (NHRS, DEUA & PBZT/MBC): // NHRS-JLKB | 000010602–3, 10825, 10796(Alc.), 10826(Alc.) // Madagascar: Toamasina: Alaotra | Mangoro: RN2, Betsabora river | 10Km W of Moramanga: S18.92438 | E048.18273, 940 m. 06.XI.2011: | GB Nets and sieves: river and | pools: Field# MAD11-21 // Leg. J. Bergsten, R. | Bukontaite, T. | Ranarilalatiana & | H.J. Randriamihaja //

##### Diagnosis.

Close to *C. marginipennis* in habitus appearance but body shape more elongate. On average *C. mahajanga* have a broader transverse testaceous band at base of elytra (Fig. 8B), and the first elytral stria is more abbreviated anteriorly, starting at approx. one quarter to one half posterior of base. Penis shape is unique in *C. mahajanga*, bisinuate in ventral view with an expanded apex, not resembling that of any other *Copelatus* species in the *irinus* group from Madagascar.

##### Description.

Body length 5.2–6.2 mm. Body shape oblong oval, rather convex, brown to dark brown. Head ferrugineous brown, paler in front, sometimes darker around eyes, finely microreticulate and punctate, two shallow depressions between eyes. Pronotum dark brown, paler at sides. Dorsal surface with fine microsculpture and scattered punctures, lateral sides of pronotum striolate with the widest striolate area in the posterior corners. Elytra brown, paler at sides and at apex, with a broad testaceous transverse band at base (Fig. 8B). Elytral surface finely reticulate and punctate. Six clearly impressed elytral striae present and one fairly long submarginal stria; first stria abbreviated anteriorly, starting at approx. one quarter to one half posterior of elytral base; other striae (second to sixth) starting more or less at base; submarginal stria starting around middle; striae 1–5 approaching apex of the elytron, but the second and fourth are a little shorter; the sixth stria much abbreviated posteriorly and sometimes the sixth unites with fifth apically approx. 1/5 from apex.

Ventral side ferrugineous brown, metacoxa and abdominal ventrites striolate and punctate. Prosternal process similar to *C. marginipennis* but lateral parts of metaventrite slightly narrower and metacoxal lines less divergent anteriorly. Antennae, palps, pro- and mesothoracic legs brown, but metathoracic legs ferrugineous dark brown.

Male: protibia modified, widened in front, strongly angled after base, with several long spines on the outer side of distal half. Tarsomeres I–III of pro- and mesolegs enlarged, with pads of numerous setae. Penis unique: in ventral view with a bisinuate shape and a widening asymmetrical apex; in lateral view abruptly curved near middle, with a right angle of more or less 90 degrees, the basal part robust (Fig. 5B). Parameres broad (Fig. 5B).

Female: similar but smaller than the male, legs not modified.

##### Distribution.

Endemic to Madagascar. Known from several places in Mahajanga province and near Moramanga (Fig. 11B). We have seen material from around Mahajanga (Type series), Mahavavy Kinkony Reserve, Namoroka NP, Tsingy de Bemaraha NP, Ankarafantsika NP, Kamoro River south of Ankarafantsika NP and collected the species both in 2011 and 2014 at Betsabora River 6 km W of Moramanga which expanded the known distribution significantly. Note that the name Betsabora is uncertain and recovered from locals on site for this river which is a tributary to Mangoro River. Ampanihifana may be the correct name.

##### Habitat and ecology.

Type series probably collected by light trap (Pederzani and Hájek 2005), and we collected it with black light by a leaf-choked forest pool in Ankarafantsika NP. Also collected with pitfall traps and from leaf-litter in Namoroka NP before the rainy season in November, indicating the species can tolerate periods when the habitats dry up without immediately seeking new water. We collected the largest series at Betsabora River at an elevation of 940 m. This small river runs through a savannah type area with mixed woodland-grassland ecosystem subsequent to degradation and deforestation. The river site consisted of grassy, vegetation-rich margins, bays and side pools, further described in Michat et al. (2017). Around the Mahajanga area all sites are lowaltitude, 100 m or lower.

##### Comments.

*Copelatus mahajanga* was the most recently described *Copelatus* species from Madagascar (Peredzani and Hájek 2005) and was previously only known from the type series and lowland west type locality “Mahajanga env.” without further details. The discovery of the species in Betsabora River at mid-altitude in the east indicates that the species may have a much wider distribution and ecological niche. As the species is very similar to *C. marginipennis* habitually, it might be misidentified as such in collections if male genitalia were not examined.

#### Copelatus insuetus

Taxon classificationAnimaliaColeopteraDytiscidae

Guignot, 1941

573F0C84F70555E7A4F6FC4974616368

Figs 6A , 9A

Copelatus insuetus Guignot, 1941: 39.

##### Type locality.

Madagascar, Perinet [= Analamazaotra NP].

##### Type information from original description.

Based on single male specimen (holotype). Madagascar, Perinet.

##### Type material studied.

**Toamasina. Alaotra Mangoro: Moramanga**: -HT♂ (GP) (MNHN, “coll. Guignot”): Data in NHRS | JLKB | 000030227 // Madagascar | Perinet // Type [red label] // [male symbol] // *Copelatus* | *insuetus* | guign. Type //

##### Additional material studied.

**Toamasina. Alaotra Mangoro: Moramanga, Ambatondrazaka, Andilamena**: -2♀ (MNHN, “coll. Guignot”): Data in NHRS | JLKB | 000030228–9 // Perinet // INSTITUT | SCIENTIFIQUE | MADAGASCAR // [female symbol] // -5♀ (NMW): Data in NHRS | JLKB | 000030302–6 // Madagascar (Md 4/5) | Andasibe NP, Perinet | 1250 m Pfütze im Urwald | 5.12.2000, leg. W. Dolin // -1♂ (NMCP, “coll. Hájek”): Data in NHRS | JLKB | 000030312 // Madagascar 10.1.2007 | Andasibe NP. | Lokato-near Andasibe | Mracek Z. leg. // coll. Jiri HÁJEK | National Museum | Prague, Czech Republic // -1♀, 1♂ (NMW): Data in NHRS | JLKB | 000030307–8 // E-Madagascar (10) | Ambatombe near Andilamena | 900 m asl. 17.01.1995 | G. Dunay & J. Janák coll. -1♀, 1♂ (NMW): Data in NHRS | JLKB | 000030309–10 // E-Madagascar (11) Ampamoho | near Andilamena, 1200–1300 m. | asl. 18–20.01.1995 | G. Dunay & J. Janák coll. // -1♀ (NMW): Data in NHRS | JLKB | 000030311 // Madagascar 18–20.1.1995 | 5km S Ampamoho | nr. Andilamena, 950–1000 m | Dunay & Janák (11) // -1♀ (NHMUK): // BMNH-797884 // MAD: AMPA: Moramanga: Andasibe | Andasibe NP: Chanel| P61BI02: N: -18.935: E:48.418: 944 m | 04/I/2007 Leg. Isambert et al // DNA Voucher | BMNH <797884> | MSL294:A12 // -2♀ (NHRS): // NHRS-JLKB | 000010692–3 // Madagascar: Tamatave: Alaotra Mangoro: | Analamazaotra RS; Bas fond, non-permanent | pond near trail to “lac rouge”MAD15-1 | 943 m, 18°56'26.7"S, 048°25'03.9"E, 16.III.2015 | Among vegetation and dead leaves in the pond | Leg.T. Ranarilalatiana & H.J. Randriamihaja // -1♀, 2♀ (Alc.) (NHRS): // NHRS-JLKB | 000065745, 10783 (Alc.) // Madagascar: Toamasina: Alaotra | Mangoro: Analamazaotra RS: close to | park entrance: 18.93573S; 048.41741E | 930 m. 08.XI.2011 GB Nets and sieves: | stagnant pools in tributary to | Analamazaotra river; Field# MAD11-26 // Leg. J. Bergsten, R. | Bukontaite, T. | Ranarilalatiana & | H.J. Randriamihaja // -1♂ (GP), 1♀, 3 ex. (1♂, 2♀) (Alc.) (NHRS): // NHRS-JLKB | 000010839, 65702, 10840 (Alc.) // MAD: TOAM: Alaotra Mangoro | Andasibe Mantadia NP, Analamazaotra | 250m E of park entrance: Mad14-18: | dried up river bed with stagnant pools: | 18.9357S 48.4174E: 930 m: 27.XI.2014 // Leg. J. Bergsten, R. | Bukontaite, S. Holmgren, | J.H. Randriamihaja | & T. Ranarilalatiana // -1♀ (NHRS): // NHRS-JLKB | 000010841 // MAD: TOAM: Alaotra Mangoro | Andasibe Mantadia NP, Analamazaotra | pond: 100m E from Lac vert: Mad14-15: | shallow forest pool: 18.9385S 48.4219E: | 940 m: 27.XI.2014 // Leg. J. Bergsten, R. | Bukontaite, S. Holmgren, | J.H. Randriamihaja | & T. Ranarilalatiana // -1♂ (GP), 1♀, 1♀ (Alc.) (NHRS): // NHRS-JLKB | 000065705, 65777, 10844(Alc.) // MAD: TOAM: Alaotra Mangoro | Andasibe Mantadia NP, Analamazaotra | 150 m E of park entrance: Mad14-14: | shallow partly dried out forest pond: | 18.9355S 48.4166E: 930 m: 27.XI.2014 // Leg. J. Bergsten, R. | Bukontaite, S. Holmgren, | J.H. Randriamihaja | & T. Ranarilalatiana // -1♂ (GP), 34 ex. (Alc.) (NHRS, DEUA & PBZT/MBC): // NHRS-JLKB | 000010842, 10843(Alc.) // MAD: TOAM: Alaotra Mangoro | Andasibe Mantadia NP, | Mantadia: 12Km N of park | entrance: Mad14-83: | forest pond with some | vegetation: 18.7911S | 48.4259E: 960 m: 28.XI.2014 // Leg. J. Bergsten, R. | Bukontaite, S. Holmgren, | J.H. Randriamihaja | & T. Ranarilalatiana // -6♂ (GP), 4♀, 33 ex. (Alc.) (NHRS, DEUA & PBZT/MBC): // NHRS-JLKB | 000010534–7, 10606–11, 10780(Alc.) // MAD: TOAM: Alaotra Mangoro: | Betsabora river by RN2 near | Antasmpanana village, 6Km W | of Moramanga: MAD14-81: river | with side pools: 18.9247S | 48.1828E: 900 m: 24.XI.2014 // Leg. J. Bergsten, | J.H. Randriamihaja | & T. Ranarilalatiana // -1♀ (NHMUK): // BMNH-797895 // MAD: TOAM: Ambatondrazaka | Zahamena: Zahamena NP: Stream | P60BI15:N: -17.52: E:48.721: 1075 m| 31:XI:2006: Leg. Isambert et al // DNA Voucher | BMNH <797895> | MSL294:B11 // -4♂ (GP), 1♀ 11 ex. (Alc.) (NHRS, DEUA & PBZT/MBC): // NHRS-JLKB | 000010817, 10872–4, 65700, 10880(Alc.) // Madagascar: Toamasina: Alaotra Mangoro: | Zahamena NP: Sect. Antanandava: | path towards Camp Cascade: S17.5166; | E048.7227; 1040 m. 07.III.2018; GB Nets, | white pan & sieves: Waterfilled goldigging | hole in forestswamp: Field# MAD18-80 | Leg. J. Bergsten, & T. Ranarilalatiana // -1♀ (Alc.) (NHRS): // NHRS-JLKB | 000010885 // Madagascar: Toamasina: Ambatondrazaka | Zahamena NP: the way to Camp | Cascade: 19.III.2013, GB Nets & sieves: slow | stream, Leg. J.H. Randriamihaja & | T. Ranarialalatiana: Field# ZAH13-02 // -1♀ (Alc.) (NHRS): // NHRS-JLKB | 000010881 // Madagascar: Toamasina: Alaotra Mangoro: | Zahamena NP: Sect. Antanandava: | Analamaitso forest: S17.5179; E048.7232; | 1050 m. 07.III.2018; GB Nets, white pan & | sieves: small foreststream: Field# MAD18-81 | Leg. J. Bergsten, & T. Ranarilalatiana // -3♀ (Alc.) (NHRS): // NHRS-JLKB | 000010882 // Madagascar: Toamasina: Alaotra Mangoro: | Zahamena NP: Sect. Antanandava: | Ambavahala forest: S17.5300; E048.7161; | 1070 m. 08.III.2018; GB Nets, white pan & | sieves: larger shaded forestswamp: Field# MAD18-90 | Leg. J. Bergsten, & T. Ranarilalatiana // -1♂ (GP), 2♀ (Alc.) (NHRS): // NHRS-JLKB | 000010875, 10883(Alc.) // Madagascar: Toamasina: Alaotra Mangoro: | Zahamena NP: Sect. Antanandava: | Pandanus Swamp: S17.5166; E048.7227; | 1040 m. 08.III.2018; GB Nets, white pan & | sieves: forestswamp: Field# MAD18-91 | Leg. J. Bergsten, & T. Ranarilalatiana -1♀ (Alc.) (NHRS): // NHRS-JLKB | 000010884 // Madagascar: Toamasina: Alaotra Mangoro: | Zahamena NP: Sect. Antanandava: | Analamaitso forest: S17.5225; E048.7250; | 1090 m. 08.III.2018; GB Nets, white pan & | sieves: waterfilled golddigging hole in flat: Field# MAD18-92 | Leg. J. Bergsten, & T. Ranarilalatiana // -2♂ (GP), 3♀, 6♀ (Alc.), 6ex. (3♂, 3♀) (Alc.), (NHRS, DEUA & PBZT/MBC): // NHRS-JLKB | 000010816, 10876–9, 10886(Alc.), 65753(Alc.) // Madagascar: Toamasina: Alaotra Mangoro: | Zahamena NP: Sect. Antanandava: 150 m | upstream to Camp Cascade: S17.5458; E048.7244; | 1270 m. 10–11.III.2018; GB Nets, white pan & | sieves: waterpool with dead leaves: Field# MAD18-109, | Leg. J. Bergsten, & T. Ranarilalatiana // **Toamasina. Analanjirofo: Maroantsetra**: -1♂, 1♀ (MNHN, “coll. Legros”): Data in NHRS | JLKB | 000030314-5 // Maroantsetra | Vadon IV.50 // 1/6 //? insuetus | guign. // [These two specimens were seen in MNHN, but their identity, after the discovery of additional very similar species in the complex, was not confirmed. We therefore only tentatively refer them to this species.] **Fianarantsoa. Vatovavy Fitovinany: Ifanadiana**: -1♀ (CAS): // CASENT-8131927 // Madagascar: Province | Fianarantsoa, Parc National | Ranomafana, radio tower | at forest edge, elev 1130 m | 19–26 February 2002 // 21°15.05'S, 47°24.43' | coll: M. Irwin, R. Harin’Hala | California Acad. of Sciences | malaise. Mixed tropical | forest MA-02-09B-17 // -1♂ (teneral) (NMPC, “coll. Hájek”): Data in NHRS | JLKB | 000030313 // Madagascar 26–31.I.2007 | Ranomafana NP | Ranomafana vill. env. | Z. Mracek leg. // coll. Jiri HÁJEK | National Museum | Prague, Czech Republic // **Fianarantsoa. Matsiatra Ambony: Lalangina**: -1♀ (CAS): // CASENT-5004000 // Madagascar, Fianarantsoa | Province, Ranomafana National | Park, Vohiparara village, 1160m | mixed tropical forest | 2–22 January 2001 | 21.23906S / 47.38487E | COL-DHK-2001–003 // D.H. & K.M. Kavanagh | R.L. Brett, E. Elsom, F. Vargas, | R. Ranaivosolo, E.F. Randrianirina, | N. Rasoamanana, T.J. | Ravelomanana and H.C. | Raveloson collectors // -4♂ (CAS): // CASENT-5004056, 5004070–1, 5004075 // Madagascar, Fianarantsoa | Province, Ranomafana | National Park, Vohiparara | area, 1170m mixed tropical | forest 2–22 January 2001 | 21.22644S, 47.36979E, | Stop# DHK-01-004 // D.H. & K.M. Kavanagh | R.L. Brett, E. Elsom, F. | Vargas, R. Ranaivosolo, | E.F. Randrianirina, N. | Rasoamanana, T.J. | Ravelomanana and H.C. | Raveloson collectors // **Antsiranana. Diana: Nosy-Be**: -1♂(GP) (MNHN): // Data in NHRS | JLKB | 000030230 // Nosy-Be | Sokobe [=Lokobe NP] | V. 57 Hoyt Coll // ♂ [male symbol] // **Mahajanga. Boeny: Ambato-Boeny**: -1♀ (NHRS): // NHRS-JLKB | 000010694 (JB206) // Madagascar: Mahajanga: Boeny: | Ankarafantsika NP. S16.30341 | E046.81073, 74 m.a.o. 29.XI.2009 | 22W Black Light, Field# MAD09-07 | Leg. J. Bergsten, N. Jönsson, | T. Ranarilalatiana, H.J. Randriamihaja // -1♀ (NHRS): // NHRS-JLKB | 000010781 (JB205) // Madagascar: Mahajanga: Boeny: | Ankarafantsika NP. S16.30350 | E046.81068, 87 m.a.o. 29.XI.2009 | Water Net, Field# MAD09-03 | Leg. J. Bergsten, N. Jönsson, | T. Ranarilalatiana, H.J. Randriamihaja //

##### Diagnosis.

**Figure 9.**
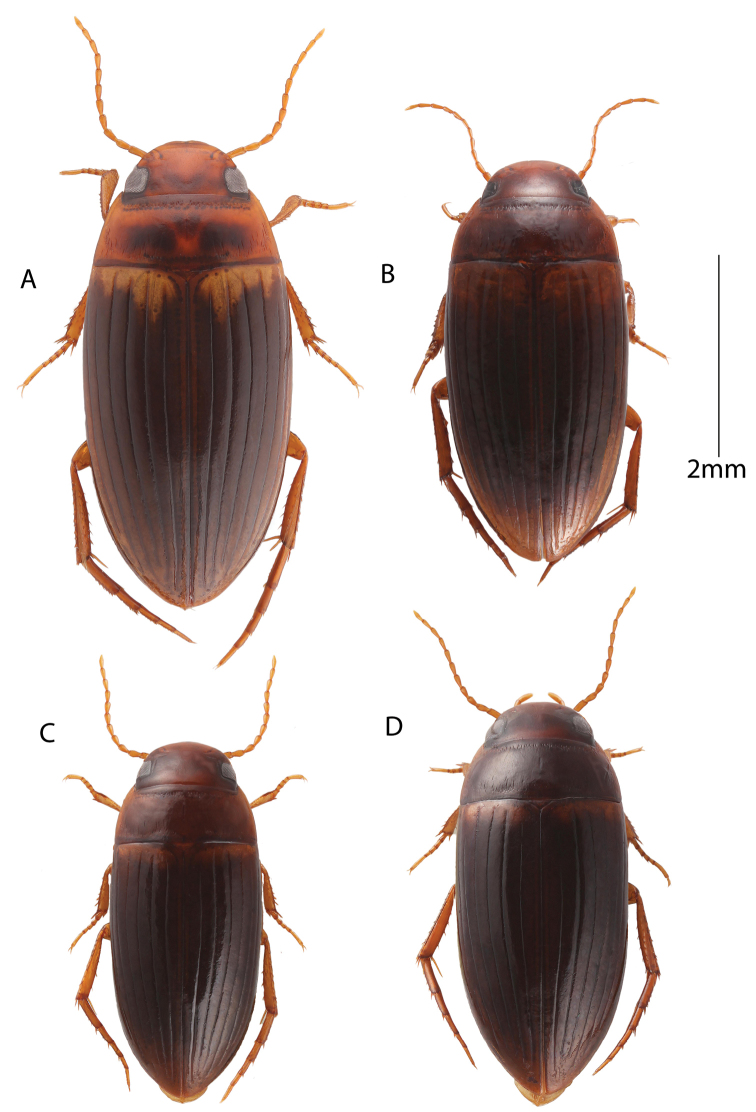
Habitus, dorsal view. **A** Female: *Copelatus insuetus***B** Male: *Copelatus vokoka* sp. nov. **C** Female: *Copelatus kely* sp. nov. **D** Female: *Copelatus* sp. 3 (Ivohibe).

Body shape elongate oval, elytra brown with basal testaceous band, with six discal and one submarginal stria (Fig. 9A). *Copelatus insuetus* can be distinguished from the following species based on the penis ventral outline in lateral view with a distinct “shoulder” (Fig. 6A), an apex in left lateral view with a dorsal ridge crossing posterior dorsal margin and medial part longitudinally coarsely sulcate ventrolaterally in right lateral view (Fig. 6A).

##### Description.

Body length 4.3–5.0 mm. Body shape elongate, weakly oval, some specimens appearing subparalell. Head rufotestaceous anteriorly and posteriorly but infuscated by two blotches inbetween the eyes. Blotches usually meet in middle and form an M-mark, but sometimes infuscation reduced to nearly absent. Pronotum usually with extensive medial infuscation leaving only lateral, anterolateral and posterolateral areas testaceous. Some specimens with less extensive medial infuscation (Fig. 9A). Elytra brown with an irregular testaceous band at base. Testaceous band with longest projection posteriorly in second interval. All appendages testaceous.

Elytra with six clearly impressed discal and one submarginal stria. Fifth and sixth striae abbreviated anteriorly, and also first stria may be somewhat abbreviated. Submarginal stria starting between one third from the base and midway between base and apex. Head, pronotum, and elytra microreticulate and finely micropunctate. Posterolateral corners of pronotum with strioles that extend along posterior surface, reduced or absent medially.

Ventral side ferrugineous brown, except prothorax, epipleura, appendages, and gula of head which are testaceous. Area around metacoxal lines lighter (not all individuals) and also spots laterally on abdominal sternites II–VII and along some sternite margins are lighter. Metacoxa and abdominal sternites II–IV marked with strioles. Prosternal process short, broad, with blunt apex. Lateral parts of metaventrite broad. Metacoxal lines long, abbreviated only slightly before metaventral margin, diverging anteriorly.

Male: first three pro- and mesotarsomeres widened and ventrally equipped with suction cups; number of suction cups per segment for I–III: 7:4:4 for both pro- and mesotarsus. Protibia modified, narrow with a bisinuate angulate ventral margin at base, broadened distally. Pro- and mesotarsal claws unmodified. Penis in ventral view thin and simple, apical part slightly left-turned. Penis in lateral view angled after basal third forming a “shoulder”. Apex in left lateral view with a characteristic dorsal ridge crossing posterior dorsal margin (Fig. 6A). Penis serrated with fine transverse ridges preapically on the left ventral ridge and halfway down the left side. Penis medially strongly longitudinally sulcate ventrolaterally in right lateral view (Fig. 6A). Parameres as in Figure 6A.

Female: anterior half of elytra finely striolated from second or third elytral interval to external margin. Degree of elytral striolation in females quite variable between specimens. These strioles are finer than the strioles on pronotum found in both sexes.

##### Distribution.

Endemic to Madagascar. Known with certainty from the eastern escarpment, Ranomafana NP in the south, Analamazaotra NP, and Mantadia NP in the central region, and Zahamena NP and Andilamena further north (Fig. 12A). We have also seen older material including males from Paris museum from lowland Lokobe NP (island of Nosy Be) in NW and Maroantsetra in NE (not reconfirmed) that correspond to the same species. Finally, two females (NHRS) from Ankarafantsika NP is likely this species.

##### Habitat and ecology.

We have collected this species in Analamazaotra NP in stagnant forest pools with vegetation or with plentiful of dead leaves. Also found in a sidepool next to a river in semi-open degraded area near Moramanga. The eastern escarpment localities range in altitude between 900–1300 m, but if the localities of Nosy Be and Maroantsetra are correct this species can also occur at lowland sea level altitudes. It seems to be most abundant in tropical eastern forests, but if the Ankarafantsika females belong to this species, then it can also occur in western deciduous forests.

##### Comments.

Eventual older records of *C. insuetus* should be regarded with caution in light of the habitually very similar new species described below.

#### Copelatus kely

Taxon classificationAnimaliaColeopteraDytiscidae

Ranarilalatiana & Bergsten sp. nov.

9FD551E5A9DF5CB19C529BDE43F5F8D0

http://zoobank.org/3AFB0F44-539F-4621-9002-F2E44EEDC73A

Figs 6B , 9C

##### Type locality.

Ambohidray reserve, Andriambe [18.61317S, 048.32593E] [Madagascar, Alaotra Mangoro region, Moramanga district].

##### Type material.

**Toamasina. Alaotra Mangoro: Moramanga**: -HT♂(GP) (NHRS): // NHRS-JLKB | 000010890 // Madagascar: Moramanga: Ambohidray | reserve: TR18L14: Andriambe stream: | S-18.61317; E48.32593; 1044m: | stagnant pool in pitfall holes: | 23/05/2018; Leg. T. Ranarialalatiana // Holotype | *Copelatus kely* sp. nov. | Det. Ranarilalatiana | & Bergsten, 2019 // **Paratypes**: -2♂(GP), 3♀, 11 ex. (7♂, 4♀) (Alc.) (NHRS, NHMUK, DEUA & PBZT/MBC): // NHRS-JLKB | 000010891, 65741–4, 10889(Alc.) // Madagascar: Moramanga: Ambohidray | reserve: TR18L14: Andriambe stream: | S-18.61317; E48.32593; 1044m: | stagnant pool in pitfall holes: | 23/05/2018; Leg. T. Ranarialalatiana // Paratype | *Copelatus kely* sp. nov. | Det. Ranarilalatiana | & Bergsten, 2019 // -2♂GP (teneral), 2♀, 10 ex. (Alc.) (teneral) (NHRS): // NHRS-JLKB | 000010858–9, 65738–9, 10861(Alc.) // Madagascar: Moramanga: Ambohidray | reserve: TR18L04: Andriambe stream: | S-18.6132; E48.3262; 1044m: | stagnant pool in pitfall holes: | 07/04/2018; Leg. T. Ranarialalatiana & al. // Paratype | *Copelatus kely* sp. nov. | Det. Ranarilalatiana | & Bergsten, 2019 // -2♀ (teneral), 5 ex. (Alc.) (teneral) (NHRS): // NHRS-JLKB | 000065701, 65740, 10862(Alc.) // Madagascar: Moramanga: Ambohidray | reserve: TR18L07: Andriambe stream: | S-18.6131; E48.3257; 1046m: | stagnant pools in path: | 07/04/2018; Leg. T. Ranarialalatiana & al. // Paratype | *Copelatus kely* sp. nov. | Det. Ranarilalatiana | & Bergsten, 2019 // -1♂(GP) (NHRS): // NHRS-JLKB | 000065746 // MAD: TOAM: Alaotra Mangoro | Andasibe Mantadia NP, Analamazaotra | 250m E of park entrance: Mad14-18: | dried up river bed with stagnant pools: | 18.9357S; 48.4174E: 930m: 27.XI.2014 // Leg. J. Bergsten, R. | Bukontaite, S. Holmgren, | J.H. Randriamihaja | & T. Ranarilalatiana // Paratype | *Copelatus kely* sp. nov. | Det. Ranarilalatiana | & Bergsten, 2019 //

##### Diagnosis.

Similar body shape to *C. insuetus* but *C. kely* is smaller than all other species in the *Copelatus insuetus* complex on Madagascar; body length 3.8–4.4 mm (Fig. 9C). The penis is most similar to that of *C. insuetus* but the “shoulder” in lateral view is less distinct; there is no crossing of a dorsal ridge against the posterior dorsal margin at apex in left lateral view and longitudinal ventrolateral sulcation is lacking (Fig. 6B).

##### Description.

Body length 3.8–4.3 mm. Body shape elongate oval to subparallel. Head rufo-testaceous and only vaguely infuscated medially and around eyes. Pronotum largely rufotestaceous with only faint infuscation medially. Lateral margins lighter testaceous but pronotum also medially lighter than elytra. This gives the habitus appearance of a lighter more rufous anterior part of body contrasting with brown elytra. Elytra brown with a testaceous band basally. Testaceous band narrower and with less of a tendancy to be extended posteriorly in second interval (Fig. 9C) compared with *C. insuetus*. All appendages testaceous.

Elytra with six impressed discal and one submarginal striae. First to fourth striae full length or first stria slightly abbreviated at base; fifth and sixth striae abbreviated anteriorly; submarginal stria starting approx. one third to one half from the base. Head, pronotum, and elytra microreticulate and finely micropunctate. Striolation of pronotum rather restricted and present only in posterolateral corners and somewhat inwards along posterior margin but not posteromedially.

Ventral side largely testaceous to faintly infuscated, similar to *C. insuetus* but lighter. Prosternal process and lateral parts of metaventrite similar to *C. insuetus* but metacoxal lines less strongly diverging anteriorly.

Male: first three pro- and mesotarsomeres widened, but less so than in *C. insuetus*, and ventrally equipped with suction cups (same constellation as in *C. insuetus*). Protibia modified, bisinuate and angled basally and broadened distally. Pro- and mesotarsal claws unmodified. Penis thin and simple, in ventral view with apical part slightly leftturned; in lateral view slightly angled after basal third giving a suggested “shoulder” (Fig. 6B) but which is less distinct compared with *C. insuetus*. Apex in left lateral view without a dorsal ridge crossing posterior dorsal margin, but finely serrated preapically (only visible at high magnification). Parameres as in Figure 6B.

Female: with very weak, faint and dispersed strioles on anterior half of elytra from third or fourth interval to the lateral margin.

##### Etymology.

The new species is named after the Malagasy word for small, “kely”, referring to the small body size. It is the smallest species so far known from the *C. insuetus* species complex on Madagascar. It is a non-latinised adjective.

##### Distribution.

Known from the eastern central part of Madagascar, at Ambohidray Reserve and in Analamazaotra NP (Fig. 12B).

##### Habitat and ecology.

This species occurs in the eastern central rainforest. Most specimens were collected from small waterfilled pitfalltrap holes for *Mantella* frogs, but we’ve also found it in muddy, stagnant, forest pools with dead leaves. The altitude of known localities ranges from 930 to 1050 m. At Analamazaotra NP, we found one specimen occurring sympatrically with *Copelatus insuetus* at the same locality.

##### Comments.

This species may be endemic to a very limited area and apart from one specimen found in Analamazaotra NP, we collected the remaining series from the Ambohidray reserve. Most specimens were in fact found in water-filled pitfall trap holes set for a microendemic *Mantella* frog species. *Copelatus kely* adds to the importance of this reserve for conservation of rare and microendemic eastern rainforest species. Ambohidray reserve was established in 2013 and managed through collaboration between the local people association (VOI MMA) and Antananarivo University. During fieldwork at the reserve in April 2018 however, we observed worrying signs of disturbances; “tavy” or slash and burn of the forest for agriculture, the cutting of woods for charcoal, signs of zebu-cattle along forest paths. These factors could cause a serious threat to the aquatic insect fauna of the reserve. The reserve of Ambohidray harbours some species not known from anywere else on Madagascar. If the reserve has any ambition to serve as a refugium for these species, activities destroying or degrading the forests or aquatic habitats should be avoided. *Copelatus kely* is very close to *C. insuetus*, and the two species were not reciprocally monophyletic in the CO1 gene tree (Fig. 2).

#### Copelatus vokoka

Taxon classificationAnimaliaColeopteraDytiscidae

Ranarilalatiana & Bergsten sp. nov.

585E67A8A2805A19B8996B9FBA19B8AA

http://zoobank.org/8FF486E9-5A55-4581-9CE8-5990C65FAF6D

Figs 6C , 9B

##### Type locality.

Ivohibe Special Reserve [22.456683S, 46.956283E] [Madagascar, Ihorombe region, Ivohibe district]

##### Type material.

**Fianarantsoa. Ihorombe: Ivohibe**: -HT♂ (GP) (NHRS): // NHRS-JLKB | 000010849 // Madagascar: Fianarantsoa: Ihorombe: R.S. | Pic d’Ivohibe Corridor: at the confluence of | two rivers Inganga and Anefitany: | S22.456683; E046.956283; 874 m; 09.XII.2013 | GB Nets & sieves: forest pools with dead | leaves, Leg. J.H. Randriamihaja & | T. Ranarialalatiana: Field# MAD13-55 // Holotype | *Copelatus vokoka* sp. nov. | Det. Ranarilalatiana | & Bergsten, 2019 // **Paratypes**: -6♂ (GP), 2♀, 16 ex. (6♂, 10♀) (Alc.) (NHRS, NHMUK, DEUA & PBZT/MBC): // NHRS-JLKB | 000010850–4, 10857, 65735–6, 10855(Alc.) // Madagascar: Fianarantsoa: Ihorombe: R.S. | Pic d’Ivohibe Corridor: at the confluence of | two rivers Inganga and Anefitany: | S22.456683; E046.956283; 874 m; 09.XII.2013 | GB Nets & sieves: forest pools with dead | leaves, Leg. J.H. Randriamihaja & | T. Ranarialalatiana: Field# MAD13-55 // Paratype | *Copelatus vokoka* sp. nov. | Det. Ranarilalatiana | & Bergsten, 2019 // -1♂ (GP), 1♀, 3♀ (Alc.) (NHRS): // NHRS-JLKB | 000010512–3, 10782(Alc.) // Madagascar: Fianarantsoa: Ihorombe: | R.S. Pic d’Ivohibe Corridor: The | confluence of rivers Inganga and | Anefitany: S22.457283; E046.95535; 870 m, | 09.XII.2013, GB Nets & sieves: big | muddy pool, Leg. J.H. Randriamihaja & | T. Ranarialalatiana: Field# MAD13-57 // Paratype | *Copelatus vokoka* sp. nov. | Det. Ranarilalatiana | & Bergsten, 2019 // **Fianarantsoa. Vatovavy Fitovinany: Ifanadiana**: -1♂ (GP), 4♂, 11♀ (coll. Michaël Manuel, Paris, NHRS): // Madagascar. Ex-prov. Fiana- | rantsoa. ca. 3.3 km WSW | Ranomafana. 28 XII 2017. | Ramahandrison & Manuel leg. // 21°16'05"S, 47°25'28"E Alt. 993 m. | Shallow shaded pond with large | quantity of dead tree leaves and | some Poaceae, in forest. | Ranomafana NP. // Coll. | M. Manuel | Paris // Paratype | *Copelatus vokoka* sp. nov. | Det. Ranarilalatiana | & Bergsten, 2019 //

##### Diagnosis.

Body shape slightly shorter, less elongate and slightly more oval than *C. insuetus*, and eyes smaller. Elytral striae more deeply impressed and intervals therefore slightly more convex (Fig. 9B). Penis shape in lateral view gently curved from base to apex (Fig. 6C), lacking the defined “shoulder” of *C. insuetus* and *C. kely*. Penis more or less straight in ventral view, which separates the species from all other in the *insuetus* complex.

##### Description.

Body length 3.9–4.5 mm. Body shape elongate oval, but slightly less elongate compared with *C. insuetus*. Head rufotestaceous with only a faint suggesstion of an M-shaped infuscation between eyes. Pronotum rufotestaceous as the head, with weak medial infuscation Elytra dark brown, with testaceous band at base. Testaceous band generally broader and more diffusely transitioning into the darker elytral colour posteriorly (Fig. 9B) compared with *C. insuetus*. Antennae, palps and legs testaceous.

Elytra with six discal and one submarginal striae. First to fourth more or less full length, fifth and sixth slightly abbreviated anteriorly; submarginal stria starting approximately one half to one third from base. Striae more deeply impressed and intervals therefore slightly more convex compared to *C. insuetus*. Head, pronotum, and elytra microreticulate and finely micropunctate. Pronotum extensively and coarsely striated on posterior surfaces, although reduced posteromedially. On some specimens striolation covers most of pronotum, also on anterior surfaces, but is reduced medially.

Ventral side entirely testaceous except metacoxal plate which is variably infuscated, especially laterally in some individuals; metacoxa and abdominal sternites II–IV striolate. Prosternal process with a slightly more pointed apex than in *C. insuetus* and *C. kely*, and lateral parts of metaventrite not as broad. Metacoxal lines long and not strongly diverging, as in *C. kely*.

Male: first three pro- and mesotarsomeres widened, ventrally equipped with suction cups (same constellation as in *C. insuetus*). Protibia modified, bisinuate and angled basally and broadened distally. Pro- and mesotarsal claws unmodified. Penis long, thin and simple; apex in ventral view straight and not leftturned; in lateral view rather evenly arched, lacking the distinct “shoulder” characteristic of *C. insuetus* but with a different type of postmedial and preapical suggested humps in the curvature. Apex in lateral view also broder closer to apex. Apex in left lateral view with a dorsal ridge crossing posterior dorsal margin but at a more acute angle (Fig. 6C) compared with *C. insuetus*. Preapically left side with fine transverse ridges. Right lateral and ventral side with fine longitudinal microsculpture but not forming coarse sulci as in *C. insuetus*. Parameres as in Figure 6C.

Female: very faint to no striolation on outer elytral intervals in the series from Ivohibe. All females from Ranomafana NP were densely striolated over the entire elytral surface except at the apical part, and entire pronotum. Density of striation approx. 5–8 striae in breadth across each elytral interval.

##### Etymology.

Vokoka is a Malagasy adjective for curvature, also used for an old person with a hunched back. Here it refers to the even curvature of the male aedeagus in lateral view where the even curvature sets it apart from *C. insuetus*. It is a non-latinised adjective.

##### Distribution.

Known from the mountainous escarpment of southeastern Madagascar at Ivohibe Special Reserve and Ranomafana NP (Fig. 12B). Our sampling is rather scant in the humid forests south of Ivohibe, for instance down to Andohahela NP so it is possible the species distribution continues further south.

##### Habitat and ecology.

We collected this species in 2013 from forest pools with dead leaves next to streams at the Ivohibe reserve in pristine humid forest at an altitude of 870 m. The reserve of Pic d’Ivohibe was established in 1964 and is managed through collaboration between Madagascar National Parks, local people associations, and other partners. During our visit in 2013, there were little signs of degradation except at the entrance and at the west edge of the reserve. Local people sometimes take zebu cattle with them on a path through the forest. At a larger scale zebu excrement could influence freshwater quality and species assemblages through eutrophication, but there were no signs of this inside the intact pristine forest. In Ranomafana NP it was collected in a shallow clear-water shaded forest pond with large quantity of dead tree leaves and some Poaceae (M. Manuel pers. comm.).

##### Comments.

*Copelatus vokoka* sp. nov. falls in the irinus group, based on the number of elytral striae. The new species is most closely related to *C. ankaratra* according to the mitochondrial gene CO1 and belongs to the *C. insuetus* species complex on Madagascar.

#### Copelatus ankaratra

Taxon classificationAnimaliaColeopteraDytiscidae

Ranarilalatiana & Bergsten sp. nov.

2CC38FF3A07F541E8F8A801E6B92EAB4

http://zoobank.org/2E3F790E-F146-4865-8BBE-4B521FF74094

Figs 6D , 10A

##### Type locality.

Manjakatompo Ankaratra Reserve, Tsiafajavona mountain [19.35163S, 47.24278E] [Madagascar, Vakinankaratra region, Ambatolampy district]

##### Type material.

**Antananarivo. Vakinankaratra: Ambatolampy**: -HT♂ (GP) (NHRS): // NHRS-JLKB | 000065412 // Madagascar: Ambatolampy: Manjaka- | tompo Ankaratra Reserve: MAD16-11: | Tsiafajavona mountain: S-19.35163; | E47.24278; 2597 m: stream near source: | 07/02/2016; Leg. T. Ranarialalatiana // Holotype | *Copelatus ankaratra* sp. nov. | Det. Ranarilalatiana | & Bergsten, 2019 // **Paratypes**: -12♂ GP, 10♀, 77 ex. (Alc) (NHRS, NHMUK, DEUA & PBZT/MBC): // NHRS-JLKB | 000010644–59, 10863–7, 65704, 10778(Alc.) // Madagascar: Ambatolampy: Manjaka- | tompo Ankaratra Reserve: MAD16-11: | Tsiafajavona mountain: S-19.35163; | E47.24278; 2597 m: stream near source: | 07/02/2016; Leg. T. Ranarialalatiana // Paratype | *Copelatus ankaratra* sp. nov. | Det. Ranarilalatiana | & Bergsten, 2019 // -6♂ (GP), 3♀, 60 ex. (Alc) (NHRS, DEUA & PBZT/MBC): // NHRS-JLKB | 000010612–7, 65750–1, 10870, 10776(Alc.) // Madagascar: Antananarivo: | Vakinankaratra: Manjakatompo Stn | forestière: Anosiarivo: S19.344889 E | 047.304139, 2073 m. 24.I.2012: lake near source | Field# MJK12-13 : Leg. T. | Ranarilalatiana & J.H. Randriamihaja // Paratype | *Copelatus ankaratra* sp. nov. | Det. Ranarilalatiana | & Bergsten, 2019 // -12♂ GP, 6♀, 115 ex. (Alc.) (NHRS, DEUA & PBZT/MBC): // NHRS-JLKB | 000010628–43, 10868–9, 10777(Alc.) // Madagascar: Ambatolampy: Manjaka- | tompo Ankaratra Reserve: MAD16-08: | Anosiarivo: S-19.34505; E47.30384; | 2062 m: large pond in stream source: | 06/02/2016; Leg. T. Ranarialalatiana // Paratype | *Copelatus ankaratra* sp. nov. | Det. Ranarilalatiana | & Bergsten, 2019 //

##### Diagnosis.

The best diagnostic character for the species is the angled base of penis in posteroventral view (Fig. 6D) which separates the species from all other species in the *C. insuetus* complex. The penis also lacks any apical dorsal ridge crossing dorsal posterior margin in left lateral view and is devoid of any sulcation ventrolaterally other than a faint microsculpture.

##### Description.

**Figure 10.**
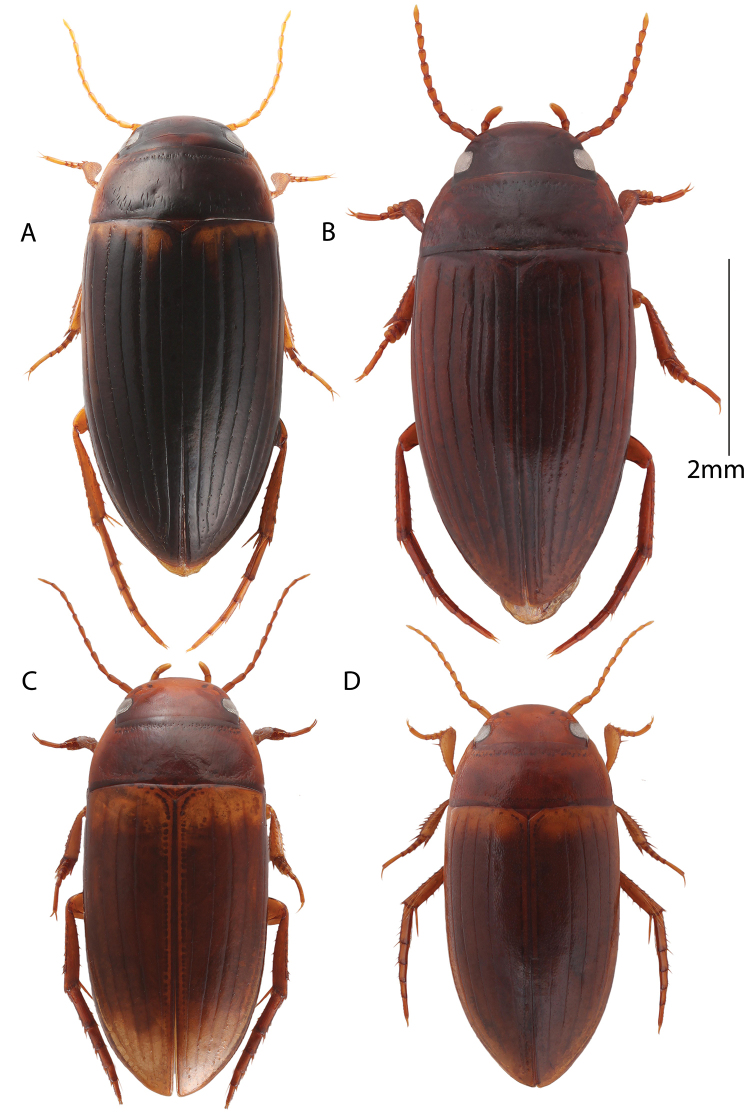
Habitus, dorsal view. **A** Female: *Copelatus ankaratra* sp. nov. **B** Male: *Copelatus pseudostriatus* sp. nov. **C** Male: *Copelatus safiotra* sp. nov. **D** Female: *Copelatus befasicus*.

Body length 4.4–5.2 mm. Body shape very elongate and subparallel. Head infuscated around eyes and medially, testaceous on clypeus and as a posterior band. Pronotum entirely infuscated except lateral margins and especially the anterolateral corners more broadly testaceous. Elytra in same dark brown colour as infuscated parts of head and pronotum, except an irregular basal testaceous band (Fig. 10A). Testaceous basal band overall narrower than in *C. insuetus*. All appendages testaceous.

Elytra with six clearly impressed discal and one submarginal stria. First to fourth elytral striae more or less full length, fifth and sixth striae slightly abbreviated anteriorly; submarginal stria starting 1/3^rd^ to 1/2 posterior of base and does not reach apex. Head, pronotum, and elytra microreticulate and finely micropunctate. Posterior third to posterior half of pronotum striolate. Strioles on average coarser than in *C. insuetus* and in some specimens more extensive onto disc and posteromedially.

Ventral side similar to *C. insuetus*, slightly darker on average so that medial light area around metacoxal lines may be more contrasting. Strioles on metacoxa rather short. Prosternal process more rhomboid and apex more strongly raised medially than in *C. insuetus*. Lateral parts of metaventrite rather broad. Metacoxal lines long and anteriorly diverging, similar to *C. insuetus*.

Male: first three pro- and mesotarsomeres widened and ventrally equipped with suction cups (same pattern as in *C. insuetus*). Protibia modified, bisinuate, angled basally, and broadened distally. Pro- and mesotarsal claws unmodified. Penis in posteroventral view distinctly angled at base (Fig. 6D) so that main blade appears tilted. Penis in lateral view evenly curved, lacking a distinct “shoulder”, long, thin, and simple apex without a dorsal ridge crossing posterior dorsal margin in left lateral view. Faint longitudinal microsculpture visible at high magnification but lacking coarse longitudinal sulci ventrolaterally and lacking serrations as in *C. insuetus*. Parameres as in Figure 6D.

Female: all but one specimen examined lack finer elytral striolation and is in elytral structure similar to males. However, the entire pronotum and elytra except apical quarter are coarsely longitudinally striolate in one female specimen examined. Striolation coarser and made up of longer irregular but connected strioles and very different to the short separate fine strioles seen in external intervals of *C. insuetus* females. Density approx. 5–7 strioles in breadth across an elytral interval.

##### Etymology.

The new species is named after the mountain massif Ankaratra where it was found and in honour of the newly created Ankaratra Massif Reserve in 2015. The epithet is a noun in apposition (ICZN 11.9.1.2).

##### Distribution.

Known only from a few localities in the Ankaratra Massif Reserve on the central highland plateau of Madagascar (Fig. 12C).

##### Habitat and ecology.

This new species was collected in the mountains of Ankaratra at altitudes above 2000 m. The first locality, Anosiarivo forest, consisted of water from a source flowing over grass vegetation at an altitude of 2070 m. The second locality, Tsiafajavona Mountain, was a small stream with grass vegetation downstream but very near to the source at an altitude of 2600 m near Ankaratra peak. *Copelatus ankaratra* seems to be a high-altitude alpine species associated with spring water.

##### Comments.

*Copelatus ankaratra* sp. nov. belongs to the *irinus* group, based on the number of elytral striae. It belongs to the *Copelatus insuetus* species complex radiation on Madagascar and based on its CO1 it is most closely related to *C. vokoka* from Ivohibe. Notably, both these species have a densely striolated female form. There was a surprisingly large genetic distance (2.3–2.5% uncorrected p-distance) between the locality near the peak (2600 m) and the locality in the forested region ca. 5 km away at a lower altitude (2070 m). We find the male genitalia and other characters very similar and treat them here as conspecific.

The Ankaratra Massif is an area known for several microendemic species not known from anywhere else on Madagascar. This includes two critically endangered micro-endemic frogs, *Boophis williamsi* (Guibé, 1974) and *Mantidactylus pauliani* Guibé, 1974 only found in montane streams at elevations above 2000 m.

Manjakatompo forestry station was established in 1923 in the forested part of the mountains to preserve an area of 8320 ha, out of which only 650 ha is natural forest and 2300 ha has been replanted with exotic trees (Nicoll and Langrand 1989; Goodman et al. 1996). Even the natural forest part is largely composed of secondary forest mixed with exotic trees. The forests still support endangered highland fauna and are important sites for some of the last remaining central plateau forests (Hjalmarsson et al. 2013). The higher elevation of the Ankaratra Massif has, until recently, lacked any protection despite the unique faunal components and characteristics (Vences et al. 2002; Goodman et al. 1996). The area has suffered severe degradation due to anthropogenic activities, mainly heavy deforestation and fire (Rabemananjara et al. 2012), but also overgrazing by livestock and expanding potato farming (IUCN SSC Amphibian Specialist Group 2016). However, in 2015 the Ankaratra Massif Reserve was created which encompasses Manjakatompo Special Reserve and the higher elevations where the endangered montane frogs are found (Moore 2015). It is managed by the local Malagasy conservation association (VIF) in collaboration with Ministère de l’Environnement, de l’Ecologie et des Forêts. This is one of the results of an ambitious conservation programme involving habitat restoration, alternative livelihood initiatives, and public awareness (Rabemananjara et al. 2012). Nevertheless, during a visit as late as 2016, there were many signs of continuous degradation from “tavy” slash and burn agriculture, commercial logging, and charcoal extraction exposing forest streams. Very large parts of the forest were burnt, causing erosion that spills into the streams. Manjakatompo forest and the Ankaratra Massif are very important forest and montane refugia of highland fauna (Vences et al. 2002; Goodman et al. 1996; Hjalmarsson et al. 2013; Andreone et al. 2014), and it should be highly prioritised for protection; we hope that the new reserve status on paper will lead to actual changes in practice.

#### Copelatus pseudostriatus

Taxon classificationAnimaliaColeopteraDytiscidae

Ranarilalatiana & Bergsten sp. nov.

5433FAE18DE15C1298549264274DB2EF

http://zoobank.org/576CE645-9356-483E-806F-3030D772B260

Figs 4C , 10B

##### Type locality.

Tsaratanana reserve, Antetikalambazaha Camp [14.1824S, 48.9448E] [Madagascar, Sofia region, Bealanana district].

##### Type material.

**Mahajanga. Sofia: Bealanana**: -HT♂ (GP) (NHRS): // BMNH-672729 // HOLOTYPE // Madagascar: Tsaratanana NP | [Antetykalambazaha Camp], 14.1824S, | 48.9448E, 1700 m, 20–24.xii.2004 | P32, Leg. Lees_D, Ranaivosolo_R // DNA Voucher | BMNH <672729> | MSL027:A07 // Holotype | *Copelatus* | *pseudostriatus* sp. nov. | Det. Ranarilalatiana | & Bergsten, 2019 // **Paratypes**: -1♀ (NHRS): // BMNH-672728 // PARATYPE // Madagascar: Tsaratanana NP | [Antetykalambazaha Camp], 14.1824S, | 48.9448E, 1700 m, 20–24.xii.2004 | P32, Leg. Lees_D, Ranaivosolo_R // DNA Voucher | BMNH <672728> | MSL027:A06 // Paratype | *Copelatus* | *pseudostriatus* sp. nov. | Det. Ranarilalatiana | & Bergsten, 2019 // -1♀ (NHMUK): // BMNH-672727 // PARATYPE // Madagascar: Tsaratanana NP | [Antetykalambazaha Camp], 14.1824S, | 48.9448E, 1700 m, 20–24.xii.2004 | P32, Leg. Lees_D, Ranaivosolo_R // DNA Voucher | BMNH <672727> | MSL027:A05 // Paratype | *Copelatus* | *pseudostriatus* sp. nov. | Det. Ranarilalatiana | & Bergsten, 2019 //

##### Diagnosis.

The best diagnostic character for the species is the pseudostriae between first and second, and second and third striae (Fig. 10B), which separates the species from all other *Copelatus* species from Madagascar; the penis is gently curved from base to apex and parameres are long and thin (Fig. 4C).

##### Description.

Body length 5.3–5.6 mm. Body shape elongate oval. Head and pronotum of all three specimens exposed to DNA extraction lysis buffer which has discoloured them slightly. Colour descriptions of head and pronotum below should therefore be taken with caution and can differ somewhat from other specimens. Head and pronotum rather uniformly brown but head likely infuscated between and around eyes (more visible in one paratype) and pronotum may have been infuscated medially prior to exposure to lysis buffer. Elytra uniformly testaceous brown with a faint suggestion of a darker transverse field preapically (Fig. 10B). Appendages testaceous.

Elytra with six clearly impressed striae and one submarginal stria. Stria five distincly shorter basally and the submarginal stria starts 1/4^th^ to 1/3^rd^ posterior from the base. Between first and second, and between second and third striae, there are irregular traces of intermediate striae, or “pseudostriae”, extending from just after base until 1/4^th^ from the apex (Fig. 10B). Anteriorly they are continuous or almost so and posteriorly they become fragmented. Few traces of pseudostriae can also be seen between the third and fourth striae but only anteriorly, and these are fragmented. Head, pronotum, and elytra microreticulate and finely micropunctate. Pronotum rather weakly striolate along posterior margin, slightly more at posterolateral corners, and sporadically anteriorly and on disc.

Ventral side largely ferrugineous, a little lighter testaceous-ferrugineous around metacoxal processes, medially on the metaventrite and on sternite II. Prosternal process short, medially raised and rounded and with a fairly pointed apex. Lateral parts of metaventrite medium broad. Metacoxal lines anteriorly diverging and abbreviated well before metaventral margin. Metacoxal plate distinctly striolate with short strioles.

Male: first three pro- and mesotarsomeres widened. Protibia modified, narrow but not bisinuate with an angulate ventral margin at base, broadened, almost club-like, distally. Protarsal and mesotarsal claws unmodified. Penis in ventral view narrowed one third from apex and the very last apical tip angled to the left; in lateral view evenly and weakly curved from base to apex (Fig. 4C). Parameres as in Figure 4C, long and thin and form an evenly curved elongated triangle.

Female: elytral structure similar to male.

##### Etymology.

The name *pseudostriatus* is a compound word formed from *pseudo*- (false) and *striatus* (furrowed or striated) and refers to the intermediate non-complete striae in-between the complete continuous striae on the elytra in this species. It is the only species of *Copelatus* on Madagascar with this characteristic. The word *striatus* (masculine) is a participle (verb as adjective) in the nominative singular (ICZN 11.9.1.1).

##### Distribution.

Endemic to Madagascar, only known from the type series from Tsaratanana Massif (Fig. 12A).

##### Habitat and ecology.

Not known, but the type series of specimens were collected in 2004 likely from a stream, near Antetikalambazaha Camp at an altitude of 1700 m.

##### Comments.

Species group assignment of *Copelatus pseudostriatus* sp. nov. is hardly possible: based on the complete striae it would fall in the *irinus* group, but the incomplete pseudostriae are likely reduced striae of an ancestor with a higher number of complete striae. This is a very distinct species with no recognisable close relatives, either based on genitalia or the CO1 gene. Tsaratanana Massif contains the highest peak in Madagascar at 2876 m and possibly this species is endemic to the Tsaratanana Massif. However, Hjalmarsson et al. (2013) showed that a high-altitude diving beetle species, *Rhantus manjakatompo* Pederzani & Rocchi, 2009, collected at the same place and time as the type series of *C. pseudostriatus*, is a species shared between Tsaratanana and Ankaratra Massif in central Madagascar.

#### Copelatus safiotra

Taxon classificationAnimaliaColeopteraDytiscidae

Ranarilalatiana & Bergsten sp. nov.

851D9EEE861E5E29812C70504B087522

http://zoobank.org/137C62A2-22DD-4550-8697-9E81CED4ED45

Figs 6E , 10C

##### Type locality.

Anjanaharibe Sud reserve, [14.7414S, 049.4975E] [Madagascar, Sava region, Andapa district]

##### Type material.

**Antsiranana. Sava: Andapa**: -HT♂ (GP) (NHRS): // NHRS-JLKB | 000065415 // MAD: ANTS: Sava: Anjanaharibe | Sud NP: stream next to Camp | site: Mad14-62: medium size | sandy forest stream: 14.7414S | 49.4975E; 910 m: 14.XI.2014 // Leg. J. Bergsten, R. | Bukontaite, S. Holmgren, | J.H. Randriamihaja | & T. Ranarilalatiana // Holotype | *Copelatus safiotra* sp. nov. | Det. Ranarilalatiana | & Bergsten, 2019 // **Paratypes**: -2♀ (Alc.) (NHRS): // NHRS-JLKB | 000010785 // MAD: ANTS: Sava: Anjanaharibe | Sud NP: stream next to Camp | site: Mad14-62: medium size | sandy forest stream: 14.7414S | 49.4975E; 910 m: 14.XI.2014 // Leg. J. Bergsten, R. | Bukontaite, S. Holmgren, | J.H. Randriamihaja | & T. Ranarilalatiana // Paratype | *Copelatus safiotra* sp. nov. | Det. Ranarilalatiana | & Bergsten, 2019 // -4♂ (GP), 1♀, 12 ex. (Alc.), 3 ex. (Alc.): (NHRS, NHMUK, DEUA & PBZT/MBC): // NHRS-JLKB | 000010506–7, 10566, 10595, 65414, 10775(Alc.), 10845(Alc.) // MAD: ANTS: Sava: Anjanaharibe | Sud NP: Camp site: Mad14-70: | forest stream: 14.7414S | 49.4975E; 910 m: 16.XI.2014 // Leg. J. Bergsten, R. | Bukontaite, S. Holmgren, | J.H. Randriamihaja | & T. Ranarilalatiana // Paratype | *Copelatus safiotra* sp. nov. | Det. Ranarilalatiana | & Bergsten, 2019 // **Toamasina. Alaotra Mangoro: Ambatondrazaka, Moramanga**: -4♂ (GP), 29 ex. (Alc.), 6 ex. (3♂, 3♀) (Alc.) (NHRS, NHMUK, DEUA & PBZT/MBC): // NHRS-JLKB | 000010818, 10836–7, 65413, 10838(Alc.), 65752(Alc.) // Madagascar: Toamasina: Alaotra | Mangoro: Zahamena NP: Sect. | Antanandava: close to Camp Bemoara | S17.5108; E048.7287; 1060 m. 07.III.2018 | GB Nets, white pan and sieves: Waterfilled | goldigging holes: Field# MAD18-87 // Leg. J. Bergsten, & | T. Ranarilalatiana // Paratype | *Copelatus safiotra* sp. nov. | Det. Ranarilalatiana | & Bergsten, 2019 // -1♂ (teneral) (NHRS): // NHRS-JLKB | 000010871 // Madagascar: Toamasina: Alaotra | Mangoro: Zahamena NP: Sect. | Antanandava: Manambato stream by Camp | Cascade: S17.545; E048.7237; 1290 m. 09.III.2018 | GB Nets, white pan and sieves: large | foreststream: Field# MAD18-100 // Leg. J. Bergsten, & | T. Ranarilalatiana // Paratype | *Copelatus safiotra* sp. nov. | Det. Ranarilalatiana | & Bergsten, 2019 // -4♂ GP, 2♀, 6 ex. (1♂, 5♀) (Alc.) (NHRS, DEUA & PBZT/MBC): // NHRS-JLKB | 000010589–94, 10774(Alc.) // Madagascar: Toamasina: Alaotra | Mangoro: Mantadia NP: Waterfall | 6km from park entrance: S18.83396 | E048.43777, 1000 m, 11.XI.2011 GB | Nets and sieves: forest stream in | rainforest: Field# MAD11-37 // Leg. J. Bergsten, R. | Bukontaite, T | Ranarilalatiana & | J.H. Randriamihaja // Paratype | *Copelatus safiotra* sp. nov. | Det. Ranarilalatiana | & Bergsten, 2019 // -4♀ (Alc.) (NHRS): // NHRS-JLKB | 000010784 // Madagascar: Toamasina: Alaotra | Mangoro: Mantadia NP: River | Sahanody 9 km from park entrance: S | 18.80973 E 048.42861, 930 m. 11.XI.2011 | GB Nets and sieves: forest stream in | rainforest: Field# MAD11-34 // Leg. J. Bergsten, R. | Bukontaite, T | Ranarilalatiana & | J.H. Randriamihaja // Paratype | *Copelatus safiotra* sp. nov. | Det. Ranarilalatiana | & Bergsten, 2019 // **Fianarantsoa. Matsiatra Ambony: Lalangina**: -1♂ (GP) (NHRS): // NHRS-JLKB | 000010846 // MAD: FIAN: Matsiatra Ambony | Ranomafana NP: next to Sahamalaotra | trail entrance: Mad14-04: forest stream: | with sandy bottom: 21.2395S 49.3947E: 1130 m: 02.XI.2014 // Leg. J. Bergsten, | T. Ranarilalatiana | & S. Holmgren // Paratype | *Copelatus safiotra* sp. nov. | Det. Ranarilalatiana | & Bergsten, 2019 // **Fianarantsoa. Ihorombe: Ihosy**: -2♂ (GP) (NHRS): // NHRS-JLKB | 000010847–8 // Madagascar: Fianarantsoa: Ihorombe: | Isalo NP: 300m into the canyon de | Makis: S22.48665 E45.37966, 700 m | 13.XI.2012, GB nets and sieves: canyon | river with side pools: Leg. R. Bukontaite | & J.H. Randriamihaja: Field# MAD12-03 // Paratype | *Copelatus safiotra* sp. nov. | Det. Ranarilalatiana | & Bergsten, 2019 //

##### Diagnosis.

Somewhat similar to *C. insuetus* on habitus appearance, but sturdier, broader pronotum and head with a much greater interocular distance compared to width of eyes, and subparalell along a longer distance of body with more rapidly attenuating anterior and posterior ends; easily distinguished from all other *irinus* group species of Madagascar by the penis shape, which is of a type otherwise found in species closely related to *Copelatus owas*; penis has a large medial expansion in lateral view followed by an apical blade (Fig. 6E).

##### Description.

Body length 4.3–5.2 mm. Body shape elongate and subparallel along a very long part of the body. Pronotum and head broad and eyes small creating a very wide interocular distance. Maximum width of pronotum clearly in front of hind corners. Head rufotestaceous with weak or absent infuscation in between and posterior of eyes. Pronotum infuscated medially with broadly testaceous lateral sides. Elytra brown, with a broad testaceous transverse band basally (Fig. 10C). Testaceous band broader and transitioning posteriorly more diffusely into the brown colour compared with *C. insuetus*. Antennae, palps and legs testaceous.

Elytra with six discal and one submarginal stria. Fifth stria abbreviated anteriorly, variably also first and third striae. In some individuals especially fifth but also first and sixth striae are rudimentary or with very shallow impressions. Interval between fifth and sixth striae narrow, approx. half interval between first and second striae. Submarginal stria short, starting around middle. Head, pronotum, and elytra microreticulate and finely micropunctate. Pronotum not striolate.

Ventral side entirely testaceous except last three abdominal ventrites may be vaguely infuscated and have lighter lateral spots. Metacoxa and abdominal sternite II–IV striolate, but strioles shallower and finer than in *C. insuetus*. Compared with *C. insuetus*, the metacoxal lines are shorter, and the anterior traces suggest an inward curve towards the posterior metaventral margin. The lateral part of metaventrite is narrower than in *C. insuetus* at level of mesocoxa, equal to the width of mesofemur at middle. Posterior metaventral margin not straight but slightly angular at level of apex of mesotrochanter. Prosternal process is slightly more elongate.

Male: first three pro- and mesotarsomeres widened, ventrally equipped with suction cups. Pattern of suction cups same as for *C. insuetus* but tarsomeres not as wide and less developed as an integrated protarsal palett. Protibia modified, bisinuate, angled basally, and broadened distally. Pro- and mesotarsal claws unmodified. Penis very characteristic, rather broad and short and in lateral view with a medial expansion followed by a sharp constriction before the narrow blade-like apex (Fig. 6E) which is characteristic of several species related to *C. owas* Régimbart, 1895; apical blade curved leftwards in ventral view and serrated by transverse ridges on the convex right side. Parameres as in Figure 6E, rather long and thin with a medial emargination on the concave side.

Female: on average smaller than males (Table 3). At least some females with elytral microreticulation slightly more strongly impressed than in males and therefore appearing more matt.

**Table 3.** Measurements of body length summarised as Min, Max, Mean, and Standard Deviation (SD) for each species, separated by sex. N = number of measured individuals, F = females, M = males.

Species	Sex	N	Min	Max	Mean	SD
* C. befasicus *	F	4	4.13	4.19	4.15	0.03
	M					
* C. marginipennis *	F	26	5.16	6.32	5.75	0.30
	M	45	5.48	6.58	6.02	0.26
* C. mahajanga *	F	12	5.16	6.13	5.63	0.24
	M	16	5.42	6.19	5.88	0.20
* C. pulchellus *	F	1	5.74	5.74	5.74	
	M	3	5.48	6.06	5.83	0.30
* C. distinguendus *	F	44	5.29	6.32	5.75	0.21
	M	26	5.48	6.26	5.92	0.18
* C. peridinus *	F	7	5.74	6.58	6.05	0.27
	M	7	5.81	6.26	6.10	0.16
* C. baculiformis *	F	1	4.00	4.00	4.00	
	M					
*C. kely* sp. nov.	F	7	3.81	4.26	4.01	0.16
	M	5	3.87	4.32	4.03	0.17
* C. insuetus *	F	27	4.26	5.03	4.60	0.20
	M	24	4.26	4.90	4.61	0.17
*C. safiotra* sp. nov.	F	3	4.32	4.71	4.58	0.22
	M	13	4.52	5.16	4.86	0.19
*C. vokoka* sp. nov.	F	6	3.94	4.32	4.18	0.13
	M	8	4.06	4.45	4.29	0.12
*C. ankaratra* sp. nov.	F	19	4.39	5.03	4.70	0.17
	M	30	4.65	5.16	4.87	0.16
*C. pseudostriatus* sp. nov.	F	2	5.42	5.61	5.52	0.14
	M	1	5.29	5.29	5.29	

##### Etymology.

The species name *safiotra* is a Malagasy noun for hybrid, here referring to the unusual combination of a male genitalia type, typical of the *Copelatus owas* species complex, in a body with a 6+1 striated elytra very much resembling the *C. insuetus* complex of species. It is a non-latinised noun in apposition.

##### Distribution.

Endemic to Madagascar. This species has a rather large distribution in the eastern humid forest from Anjanaharibe Sud reserve in the NE, all along the eastern escarpment including Zahamena NP, Mantadia NP, and Ranomafana NP, and even extending to the rather isolated western patch of subhumid forest at Isalo NP (Fig. 12D).

##### Habitat and ecology.

This species seems to be strongly associated with clean streams having sandy substrate in humid forests. At these localities, individuals can be found in sidepools, at margins or sites protected from waterflow (e.g., by fallen logs) where dead leaves and debris accumulate. The species has been found at altitudes between 700 and 1300 m but most numerous at elevations above 900 m in primary humid forest. The discovery in Isalo NP, in a sandy river running through a Canyon, indicates that subhumid forests may also be part of the species’ niche.

##### Comments.

*Copelatus safiotra* sp. nov. falls in the *irinus* group, based on the number of elytral striae (six discal and one submarginal striae). However, the genitalia is of a very different type and characteristic of the complex of species close to *C. owas* in the *erichsonii* group with ten discal and one submarginal striae. We hypothesize that this species, despite the body shape and number of elytral striae, is not related to the other species treated here, but belongs to the radiation of species around *C. owas*. This would reinforce the idea that the number of elytral striae is a very homoplastic character and not reliable to create phylogenetically sound groups (Balke et al. 2004).

### Additional non-*erichsonii* group species of Madagascar

We are aware of several *Copelatus* species not of the *erichsonii* group on Madagascar but of which we have only seen females. Some of these are undoubtedly new, confirmed with DNA data (Figs 2, 3), but we refrain from formally naming these prior to the discovery of males. We discuss these below and list the examined material.

#### Copelatus

Taxon classificationAnimaliaColeopteraDytiscidae

(Bemaraha): sp. 1

ea805ca0-ac56-5ec7-aeed-92758eebdba0

Fig. 8C

##### Type material studied

[**of *C. nodieri* & C. nodieri var. somalicus for comparison with sp1**]: -LT♀ (MNHN, “coll. Guignot”): // Data in NHRS | JLKB | 000065417 // Haut Sénégal | Khayes | Dr. Nodier | 11–12. 1881 // Type [red label] // ♀ [female symbol] // *nodieri* // Coll. Guignot // -PLT♀ (MNHN, “coll. Régimbart”): // Data in NHRS | JLKB | 000065418 // Khayes | H Senegal // *Nodieri* Reg // Coll. Régimbart // -PT♀ (MNHN, “coll. Guignot”): // Data in NHRS | JLKB | 000065419 // SOMALIA IT. | Belet Amin. | (Giuba) Apr. 1923 | Patrizi // Paratype [red label] // ♀ [female symbol] // Museo Civico | di Genova // *Copelatus* | *nodieri* | var. ♀ *somalicus* | Guign. Paratype // var. somalicus | Det. J. Bergsten, 2011 //

##### Additional material studied.

**Mahajanga. Melaky: Antsalova**: -1♀ (CAS): // CASENT 8135000 // Madagascar: Mahajanga | Prov Parc National Tsingy | de Bemaraha, 10.6 km ESE | 123° Antsalova, Elev 150 m | 16–20 November 2001 // 19°42'34"S, 44°43'5"E |coll: Fisher, Griswold et al | California Acad. Of Sciences | sifted litter - tropical dry forest | on Tsingy, BLF4432 // *Copelatus* sp. nov. | *C. pulchellus* complex | Det. Ranarilalatiana | & Bergsten, 2019 //

##### Description.

This species is broad and oval in body shape and belongs to the *pulchellus* complex. Three species of the *pulchellus* complex are known from Madagascar, *C. pulchellus*, *C. marginipennis*, and *C. mahajanga*. It is most similar to *C. marginipennis* based on its very short and broad body shape. However, it differs in being almost entirely black dorsally, only slightly rufous laterally on the elytra, pronotum, and part of head. As such it is most similar in colouration to the dark form of *C. pulchellus* on Madagascar, but the body is shorter and broader. A second distinguishing characteristic is the very abbreviated first stria, present only in the posterior third, hence abbreviated even more than in *C. mahajanga*. Finally, this female specimen has fine but dense striolations mediolaterally on the elytra, and is densely punctured in the posterolateral corners of the pronotum (Fig. 8C). This undoubtedly represents a fourth species of the group present on Madagascar, which was confirmed based on the DNA data sequenced from this unique dry-preserved female specimen (Figs 2, 3). It is intriguing that it was collected through litter sifting on the tsingy (stone carst formations) of Tsingy de Bemaraha NP.

**Figure 11.**
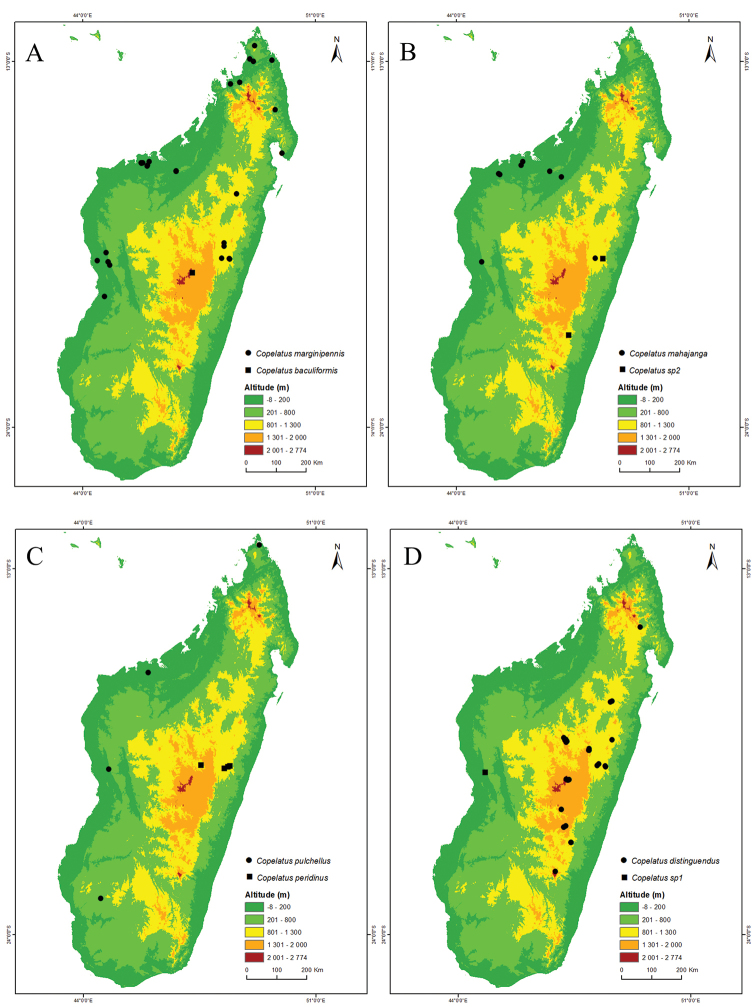
Distribution maps of *Copelatus* species. **A***C. marginipennis* (circle), *C. baculiformis* (square) **B***C. mahajanga* (circle), *C.* sp. 2 (square) **C***C. pulchellus* (circle), *C. peridinus* (square) **D***C. distinguendus* (circle), *C.* sp. 1 (square).

It is plausible that it was one or several female specimens of this species that Guignot (1960:101) referred to as *Copelatus nodieri* var. ♀ *somalicus*, a species described from Mali, continental Africa. He recorded it from “Andobo, 190 m, foret Antsingy, dct Antsalova”, the same locality as for our female (Fig. 11D). We consider Guignot’s record as based on a misidentification and remove *C. nodieri* from the list of *Copelatus* species known from Madagascar after having studied the type material of both *C. nodieri* Régimbart, 1895, described from Somalia and of C. nodieri var. somalicus Guignot, 1952. These were synonymised in the world catalogue by Nilsson (2001). We are not convinced that they should be synonyms, but we do not formally elevate var. somalicus here as we have not seen enough continental material to evaluate character variations. We have studied female type specimens of both species and compared them with the new female referred to here as *Copelatus* sp. 1: it is similar to *C. nodieri* in that the first stria is present only in the posterior third. It differs from *C. nodieri* in that the elytra are largely black and lack a basal transverse testaceous band. It also differs in having a large lateral patch of anastomosing striolations not found in *C. nodieri*. *Copelatus* sp. 1 is similar in colour to C. nodieri var. somalicus and in having anastomosing striolation on the elytra. The striolation type differs markedly in expanse on the elytra, in impression, and in density. The striolations of the elytra reach the base in C. nodieri var. somalicus but starts first after the anterior third or fourth in *Copelatus* sp. 1. The striolation is formed by shorter, shallower, and denser strioles in *Copelatus* sp. 1 so that there are approx. ten parallel strioles medially across the width of the fourth interval on elytra. The strioles are longer and more deeply impressed in C. nodieri var. somalicus and only 5–6 strioles fit across the fourth interval medially on elytra. Finally, the posterolateral corners of the pronotum are striolated in C. nodieri var. somalicus but punctated in *Copelatus* sp. 1. Therefore, we conclude that *Copelatus* sp. 1 cannot be conspecific with either *C. nodieri* Régimbart or C. nodieri var. somalicus Guignot. It is more likely a new endemic species of Madagascar, the male of which should be sought for SE of Antsalova, Tsingy de Bemaraha NP, western Madagascar. We managed to sequence partial CO1 (311 bp) of this female which differed substantially from the three other species in the complex (K2P: 8.2–13.6%). DNA matching of the male, once discovered, would be straightforward.

#### Copelatus

Taxon classificationAnimaliaColeopteraDytiscidae

(Andasibe and Ranomafana): sp. 2

0aac52e2-8b52-5955-a42c-c87201c92ee7

Fig. 7C

##### Material studied.

**Toamasina. Alaotra Mangoro: Moramanga**: -1♀ (coll. Wewalka): // Data in NHRS | JLKB | 000065698 // Madagascar, E, Andasibe | Analamazoatra Res. | 48°25'12.1"E /18°56'14.2"S | 938 m (26) 17.1.2015, | leg. Berger & Dostal // coll. Wewalka // Copelatus | longicornis group? | Det. Wewalka 2017 // *Copelatus* sp. nov.? | near *C. peridinus* | Det. Ranarilalatiana | & Bergsten, 2019 // **Fianarantsoa. Vatovavy Fitovinany: Ifanadiana**: -1♀ (NMW): // Data in NHRS | JLKB | 000065762 // RM: Namorona Bas. (PO221) | Loc. 1km de Vohiparara | Aff. de Namorona Riv. | 47°22'43"E, 21°13'53"S | Alt. 1200 m; 20.04.1994 | Leg. Elouard, J.-M., Sartori, M. // *Copelatus* sp. nov.? | near *C. peridinus* | Det. Ranarilalatiana | & Bergsten, 2019 //

##### Description.

This species has a configuration of elytral striae not found in any of the other species treated here. It has five discal striae and is lacking a submarginal stria and would fall in the *longicornis* species group together with *C. befasicus*. However, the five striae are likely not homologous as *C. befasicus* has an abbreviated first stria, whereas the first stria is entirely lacking in this specimen, therby creating an interstriae space double in width compared to the outer intervals. But despite the lack of the first stria, it has five striae on the central and lateral parts of elytra where *C. befasicus* only has four. In addition, the striae are very faintly impressed, intermediate between real striae and the puncture lines found in *C. peridinus*. The colouration is uniformly brownish black like in *C. peridinus* and the body size is also similar. This specimen is possibly a different species compared to all others presented here; however, we cannot rule out that intraspecific variation of *C. peridinus* ranges from two defined puncture lines to five weakly impressed striae. They are very similar in all other aspects. We managed to sequence a partial fragment of CO1 (447 bp) from the Andasibe specimen (NHRS-JLKB000065698) and this indeed showed that this specimen is closely related to, or possibly conspecific with, *C. peridinus*. Genetic distance between them was 3.0% (K2P). Such a level of intraspecific variation is not impossible but unlikely given they were from the same locality (Analomazaotra reserve). Together with the morphological characters we believe this may be a different and distinct species, but we await the discovery of a male before the identity can be established with confidence. The second female from Namorona River near Ranomafana NP (NHRS-JLKB000065762) is substantially larger (6.4 mm) than the female from Andasibe (5.6 mm) but otherwise shares the same characteristics (for distribution see Fig. 11B).

#### Copelatus

Taxon classificationAnimaliaColeopteraDytiscidae

(Ivohibe and North of Toamasina): sp. 3

70c03b1e-e8d2-5a8f-8f48-8ffe0eb3ceb6

Fig. 9D

##### Material studied.

**Fianarantsoa. Ihorombe: Ivohibe**: -3♀ (NHRS): // NHRS-JLKB | 000010856, 65699, 65734 // Madagascar: Fianarantsoa: Ihorombe: R.S. Pic | d’Ivohibe: Andaranovory: close to botanical | transect R.S. Pic d’Ivohibe: S22.47511667 | E046.9559, 1106 m, 10.XII.2013, GB Nets and | sieves: small lake with dead leaves and | vegetation, Leg. J.H. Randriamihaja & | T. Ranarialalatiana: Field# MAD13-61 // *Copelatus* sp. nov. | *C. insuetus* complex | Det. Ranarilalatiana | & Bergsten, 2019 // **Toamasina. Atsinanana: Toamasina, Toamasina II**: -1♀ (NHRS): // NHRS-JLKB | 000010811 // Madagascar: Toamasina II: Analalava | reserve: MAD17-12: S of nursery plants: | S17.71055; E49.45002; 39 m: Forest | stream with side pools: 09/03/2017; | Leg. T. Ranarilalatiana // *Copelatus* sp. nov. | C. insuetus complex | Det. Ranarilalatiana | & Bergsten, 2019 // -1♀ (NHRS): // NHRS-JLKB | 000010779 // Madagascar: Toamasina: | Antsinanana: RN2, 6Km N | Toamasina by bridge: S18.06493 | E049.37856, 0 m. 15.XI.2011, | GB Nets and sieves: river and | sidepool: Field# MAD11-52 // Leg. J. Bergsten, R. | Bukontaite, T | Ranarilalatiana & | J.H. Randriamihaja // *Copelatus* sp. nov. | *C. insuetus* complex | Det. Ranarilalatiana | & Bergsten, 2019 //

##### Comments.

**Figure 12.**
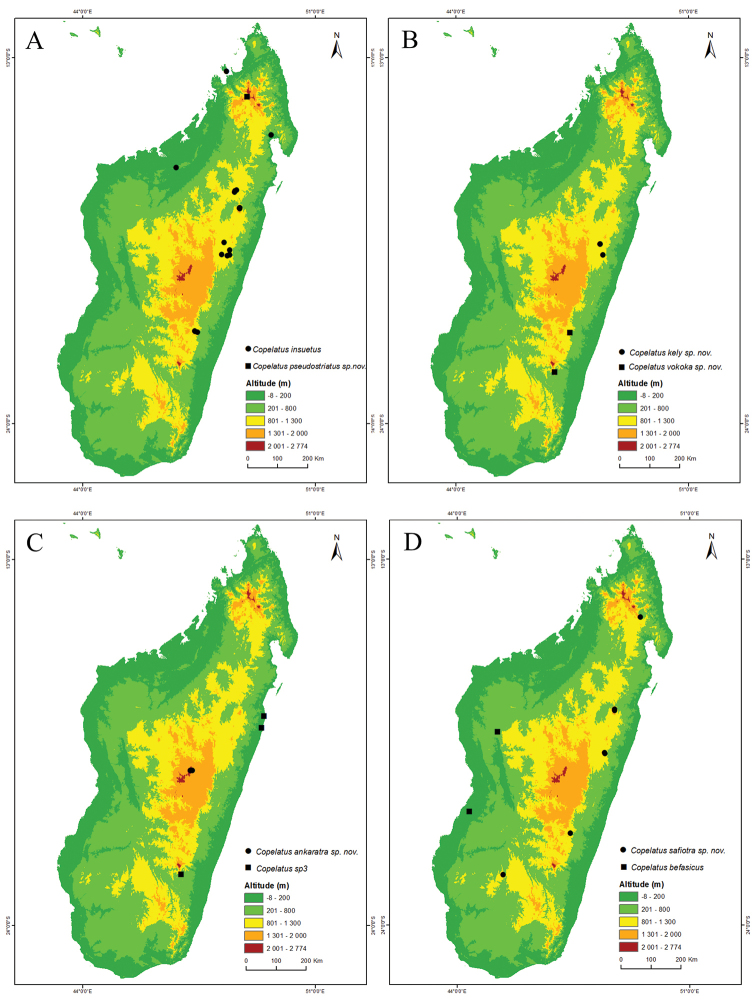
Distribution maps of *Copelatus* species. **A***C. insuetus* (circle), *C. pseudostriatus* sp. nov. (square) **B***C. kely* sp. nov. (circle), *C. vokoka* sp. nov. (square) **C***C. ankaratra* sp. nov. (circle), *C.* sp. 3 (square) **D***C. safiotra* sp. nov. (circle), *C. befasicus* (square).

The DNA data revealed that these females represent one or possibly two additional new species in the *C. insuetus* complex (Figs 2, 3). In fact, the CO1 data reveals that they are the most divergent in that group and are sister to a clade with all the other species: *C. insuetus*, *C. vokoka*, *C. kely*, and *C. ankaratra*. The genetic distance between members of these two clades ranges from a minimum of 4.5% to a maximum of 7.1%, strongly indicating a separately evolving unit. The genetic distance between the specimens from Ivohibe and those from north of Toamasina was 2.3–2.4% (K2P), a distance that does not rule out conspecificity as the geographic and altitudinal distance are substantial between these localities (for distribution see Fig. 12C). It is also on the same level as the intraspecific distance found within *C. ankaratra* between a peak population and a population at lower altitude of the Ankaratra Massif between which we do not find any morphological character differences to justify further separation. On the other hand, *C. kely* and *C. insuetus* are indistinguishable based on CO1 squences alone (Figs 2, 3). We refrain from describing these as a new species since the shape of male genitalia is very important for identification in this group. Morphologically we note the following based on the females: in body size this species is similar to *C. vokoka*, slightly larger than *C. kely* but slightly smaller than *C. insuetus* and *C. ankaratra*. Compared with females of *C. vokoka*, *Copelatus* sp. 3 has a narrower testaceous basal band, flatter elytral intervals between striae, and more limited striolation on pronotum, restricted to posterolateral corners. *Copelatus ankaratra* can be distinguished based on its dark colour and extremely elongate body shape. Small *C. insuetus* females can often be distinguished on the posteriorly extended testaceous basal medial band. It is most difficult to distinguish *Copelatus* sp. 3 females from large female specimens of *C. kely*.

## Discussion

Madagascar is known for an extremely rich endemic flora and fauna which, together with the unfortunate level of deforestation, has rewarded the island with a top spot among the world’s biodiversity hotspots (Myers et al. 2000). This high level of endemism, also among insects, is particularly manifested at species level but also at higher taxonomic level such as endemic genera (Goodman and Benstead 2003). For diving beetles (family Dytiscidae), there are currently three endemic genera. Other Dytiscidae genera like *Copelatus* are not endemic but may still contain endemic species radiations within. Here we have revised the species of *Copelatus* on Madagascar excluding the *erichsonii* species groups. We recognise 13 species with names and three additional non-named species based on females. Of the named species nine are endemic to Madagascar, two (*C. marginipennis* and *C. distinguendus*) are regional endemics to Madagascar and nearby west Indian Ocean islands, and two (*C. pulchellus* and *C. peridinus*) also occur on the African continent. This gives an endemic proportion at species level of approximately 70%, an intermediate level compared to other insect groups on Madagascar (Goodman and Benstead 2005).

The *Copelatus* diversity on Madagascar represents four of the traditional species groups in the genus based on the number of elytral striae (Sharp 1882; Balfour-Browne 1939; Guignot 1961; Guéorguiev 1968; Nilsson et al. 1997; Nilsson 2001): the *hydroporoides* (2 species), *longicornis* (1 species), *irinus* (10 species), and *erichsonii* (> 20 species) species groups. *Copelatus unguicularis* of the *consors* species group turned out to be a species of the genus *Madaglymbus*. As has been flagged before, these species groups are commonly not monophyletic (Balke et al. 2004), and in fact the number of elytral striae is a highly homoplastic character which can even vary within a single species (Bilardo and Rocchi 2008; personal observations). A better system based on phylogenetic relationships can likely be approached by using the shape of male aedeagus, and by using genetic data. We informally refer to some species complexes which we believe are groups of closely related species based on the shape of male aedeagus. Hence, the *C. insuetus* complex contains four named and one or two additional unnamed species where males are currently unknown, and we hypothesise that these constitute a hitherto unrecognised monophyletic radiation on Madagascar, to be tested with a larger sample. It is also highly likely that additional species of this complex exist on Madagascar and will be discovered in the future. This complex includes young species not yet delimitable based on the mitochondrial CO1 gene (Fig. 3). This can be due to incomplete lineage sorting or hybridisation, but they are recognisable morphologically. *Copelatus safiotra* sp. nov., in contrast, although in habitus similar to the *C. insuetus* complex, did not belong to this complex. Despite the low number of elytral striae, the male aedeagus is of the *C. owas* complex type, with a subapical expansion followed by an apical blade, and likely, phylogenetically, belongs with this group.

*Copelatus* as a genus is widespread all over Madagascar. Species can be found from the humid forest in the east to the dry forest in the west and from lowlands to the highest peaks. But the different species complexes have particular niches. Species in the *C. insuetus* complex are predominantely inhabitants of the eastern humid forests. *Copelatus distinguendus* and related species (*C. peridinus*, *C.* sp. 2, and likely *C. baculiformis*) mostly occupy open, often anthropogenically disturbed, habitats of the Central Highlands. The *C. pulchellus* complex (*C. marginipennis*, *C. mahajanga*, *C. pulchellus*, and *C.* sp. 1), and *C. befasicus* seem to be most abundant in the dry deciduous western forests, lowlands with open to semiopen landscapes. Finally, some species are specialists like the high altitude crenophile *C. ankaratra* sp. nov. and the sandy stream specialist *C. safiotra* sp. nov. Not until we have this knowledge – what are the species, how can we recognise them, where do they occur and how do they live – can we attempt to protect them and their habitats in the face of constantly increasing human pressure on the environment.

## Supplementary Material

XML Treatment for Exocelina subjecta

XML Treatment for Madaglymbus apicalis

XML Treatment for Madaglymbus unguicularis

XML Treatment for Copelatus baculiformis

XML Treatment for Copelatus peridinus

XML Treatment for Copelatus befasicus

XML Treatment for Copelatus distinguendus

XML Treatment for Copelatus pulchellus

XML Treatment for Copelatus marginipennis

XML Treatment for Copelatus mahajanga

XML Treatment for Copelatus insuetus

XML Treatment for Copelatus kely

XML Treatment for Copelatus vokoka

XML Treatment for Copelatus ankaratra

XML Treatment for Copelatus pseudostriatus

XML Treatment for Copelatus safiotra

XML Treatment for Copelatus

XML Treatment for Copelatus

XML Treatment for Copelatus

## Supplementary materials

Supplementary material 1 Examined specimens, species occurrence records Data type: species data https://binary.pensoft.net/file/322938This dataset is made available under the Open Database License (http://opendatacommons.org/licenses/odbl/1.0/). The Open Database License (ODbL) is a license agreement intended to allow users to freely share, modify, and use this Dataset while maintaining this same freedom for others, provided that the original source and author(s) are credited.Tolotra Ranarilalatiana, Lala Harivelo Raveloson Ravaomanarivo, Johannes Bergsten
